# Can fire exclusion zones enhance postfire tree regeneration? A simulation study in subalpine conifer forests

**DOI:** 10.1002/eap.70121

**Published:** 2025-10-16

**Authors:** Timon T. Keller, Diane C. Abendroth, Kristin H. Braziunas, Christina Dollinger, Paul R. Hood, Garrett J. Knowlton, Rupert Seidl, Monica G. Turner

**Affiliations:** ^1^ Department of Integrative Biology University of Wisconsin‐Madison Madison Wisconsin USA; ^2^ National Park Service Intermountain Region Denver Colorado USA; ^3^ Ecosystem Dynamics and Forest Management Group, School of Life Sciences Technical University of Munich Freising Bavaria Germany; ^4^ School of Environmental and Forest Sciences University of Washington Seattle Washington USA; ^5^ Teton Interagency Fire Grand Teton National Park Moose Wyoming USA; ^6^ Berchtesgaden National Park Berchtesgaden Bavaria Germany

**Keywords:** climate change, disturbance, ecosystem change, forest ecology, Greater Yellowstone, management, National Parks, resilience, resist‐accept‐direct, simulation modeling, wildfire

## Abstract

Climate change and novel fire regimes increasingly challenge stewardship of forests adapted to infrequent, stand‐replacing fire. Novel fire regimes may disrupt mechanisms that sustained postfire regeneration historically, and whether fire management can promote forest resilience to future fires is uncertain. We used the individual‐based forest simulation model iLand to explore how fire exclusion zones that mimic historical burn mosaics may affect postfire tree regeneration in conifer forests of Grand Teton National Park (Wyoming, USA). We asked: (1) How do the amount and configuration of potential fire exclusion zones influence postfire tree regeneration throughout the 21st century under alternative climate scenarios? (2) How do “operational” fire exclusion zones affect postfire tree regeneration within burned patches and across the landscape by the end of the 21st century? We first conducted a factorial simulation experiment with varying amounts (10%, 30%, 50% of the landscape) and configurations (dispersed vs. clumped) of fire exclusion zones. Informed by this experiment and logistical firefighting considerations, we developed an operational scenario in which we designated mature forests surrounded by defensible fuel breaks as fire exclusion zones. Simulations were conducted under four future climate scenarios (warm‐wet, hot‐wet, warm‐dry, hot‐dry), and postfire tree regeneration densities with fire exclusion zones were compared to reference scenarios without fire exclusion zones. Regeneration of fire‐avoiding conifers (subalpine fir, *Abies lasiocarpa* and Engelmann spruce, *Picea engelmannii*) was consistently greater with fire exclusion zones, especially with ≥30% of the landscape in dispersed configuration. Fire exclusion zones had minimal effects on regeneration of fire embracers (lodgepole pine, *Pinus contorta* var. *latifolia*) and fire resisters (Douglas‐fir, *Pseudotsuga menziesii* var. *glauca*). In the operational scenario, postfire regeneration of fire‐avoiding species was greater compared to the reference scenario, especially in hot climate scenarios. Although regeneration of fire avoiders declined in operational and reference scenarios throughout the 21st century, regeneration densities were up to 10 times greater in the operational relative to the reference scenario. Our results suggest that mimicking historical burn mosaics by establishing fire exclusion zones could sustain seed sources and afford more time for subalpine conifer forests to adapt to a warmer world with more fire.

## INTRODUCTION

Stewardship of forests in fire‐prone landscapes is challenged by climate‐driven increases in fire size, severity, and frequency (Abatzoglou & Williams, 2016; Overpeck et al., 1990; Parks, Holsinger, Blankenship, et al., 2023), especially where fires deviate from historical ranges of variability (HRV; Clark‐Wolf et al., 2023; Morgan et al., 1994). Management is particularly challenging in the extensive boreal and temperate conifer forests adapted to infrequent, high‐severity fires driven largely by climate (Halofsky et al., 2018; Millspaugh et al., 2000; Stephens et al., 2013) because, in contrast to dry forest types, reducing burn severity cannot sustain resilience (i.e., the ability of the ecosystem to recover to pre‐disturbance states; Holling, 1973). The dominant tree species are adapted to regenerate after stand‐replacing fires, often from seed, and resilience mechanisms depend on seed supply and environmental conditions (Stevens‐Rumann & Morgan, 2019). Changing fire regimes can erode forest resilience by limiting seed supply (Gill et al., 2021; Harvey et al., 2016; Keeley et al., 1999), and changing environmental conditions can constrain germination and establishment (Hoecker et al., 2020; Kemp et al., 2019). Although some guidelines for sustaining resilience of these forest types exist (Halofsky et al., 2018), whether management strategies can operationalize these guidelines and foster adaptation to changing fire regimes is unknown (Stephens et al., 2013).

The Resist, Accept, Direct (RAD) framework (Schuurman et al., 2020, 2022) offers three options for navigating transformational change. Managers can resist change by preventing disturbance or actively restoring an ecosystem to a reference state, accept change by not intervening, or direct change by guiding the ecosystem toward an alternative condition (Schuurman et al., 2020, 2022). Effective science–management collaborations are crucial for developing and testing plausible place‐based management strategies for each RAD option and navigating the uncertainty inherent in management decisions (Kirchhoff et al., 2013). Before implementing management strategies in real‐world landscapes, simulation experiments can explore alternative strategies under a range of future scenarios. This can help to avoid unintended future consequences (Lynch et al., 2021), as ecosystems will continue to change over time with climate (Jackson, 2021). Lastly, vulnerability assessments can further inform the choice of management strategy by identifying ecosystem components most vulnerable to changing climate (Jackson, 2021; Lecina‐Diaz et al., 2021).

Adaptations to fire vary among tree species across conifer forests of the western United States (Agee, 1993; Keeley, 2012). Thus, species differ in their vulnerability to changing fire regimes and may be more or less responsive to different management interventions. Fire avoiders (e.g., subalpine fir, *Abies lasiocarpa*; Engelmann spruce, *Picea engelmannii*; white spruce, *Picea glauca*; western hemlock, *Tsuga heterophylla*) lack adaptations to fire and rely on seed dispersal from live, mature forests. Fire avoiders may be especially vulnerable to increased fire size and frequency, as they often occur where historical fire return intervals are longer than tree lifespans. Fire resisters (e.g., Douglas‐fir, *Pseudotsuga menziesii*; ponderosa pine, *Pinus ponderosa*) have traits such as thick bark that allow individual trees to survive low‐intensity fire and retain seed sources within burned areas. However, fire resisters could also be vulnerable if greater fire intensity increases mortality and thus reduces seed supply. In contrast, fire embracers do not survive fire but are adapted to regenerate after fire from aerial seedbanks of serotinous cones (e.g., lodgepole pine, *Pinus contorta* var. *latifolia*; black spruce, *Picea mariana*) or by resprouting from surviving roots (e.g., quaking aspen, *Populus tremuloides*). Fire embracers may be less vulnerable to increased fire, except where immaturity risk eliminates the canopy seed bank (Keeley et al., 1999). Effective management interventions may be needed to enhance natural regeneration of vulnerable fire avoiders or resisters as novel fire regimes emerge.

Historical postfire mosaics offer a natural analog for a “Resist” option that could enhance postfire resilience of fire avoiders and resisters in subalpine forests. Islands of unburned forest (i.e., patches of live forest within a fire perimeter) and fire refugia (i.e., live forests that persist through successive fires) enhance postfire tree regeneration by retaining seed sources, regulating microclimate, and buffering climatic changes (Blomdahl et al., 2019; Coop et al., 2019; Downing et al., 2019). Yet, whether and where such stands may persist after a fire is difficult to predict under future climate (Mackey et al., 2021; Meigs et al., 2020; Rodman et al., 2023). As climate and fire regimes change, managers could mimic the effects of unburned stands and fire refugia by establishing fire exclusion (Fx) zones through strategic and targeted suppression of fire in forests adapted to infrequent, stand‐replacing fire (i.e., “reasoned fire exclusion” sensu Halofsky et al., 2018). Delineating Fx zones along fuel breaks before fires occur and then preventing fire from spreading into such zones could enhance postfire tree regeneration in surrounding burned areas, mimicking effects of historical burn mosaics. Whether such a strategy is feasible and effective is uncertain, and many questions remain unanswered: What proportion of a landscape would need to be managed as Fx zones? How should such zones be arranged spatially to maximize seed delivery to burned areas? If seeds disperse into burned areas, do warming temperatures push tree species beyond their physiological thresholds for germination and establishment (Davis et al., 2019; Stevens‐Rumann et al., 2022)? Assessing whether delineating Fx zones in contemporary landscapes could allow forests to adapt to changing climate and whether ecological benefits would warrant the investment could help guide management choices.

Simulation models are ideal for exploring alternative scenarios, especially when field experiments are not feasible, management goals depend on long‐term outcomes, and future climate and fire regimes are uncertain. Our team of managers and scientists used the individual‐based forest landscape model iLand (Rammer et al., 2024; Seidl et al., 2012) to explore the potential for Fx zones to enhance natural postfire tree regeneration in Grand Teton National Park (GRTE; Wyoming, USA). We first asked (1) how do the amount and configuration of potential Fx zones influence postfire tree regeneration throughout the 21st century under alternative climate scenarios? To disentangle the effects of extent and configuration, we used neutral landscape models (NLMs; Gardner et al., 1987; With & King, 1997) to simulate spatial patterns of Fx zones. Informed by the spatial arrangement that enhanced postfire tree regeneration the most, we developed an “operational” scenario. We delineated operational Fx zones in areas dominated by mature stands of vulnerable species (fire‐avoiding subalpine fir, Engelmann spruce, and fire‐resisting Douglas‐fir) and where fuel breaks and current methods of fire suppression could prevent fire spread into Fx zones. We then asked (2) relative to reference simulations without Fx zones, how do operational Fx zones affect postfire tree regeneration within burned patches and across the landscape by the end of the 21st century? We expected that greater amounts of dispersed Fx zones would increase postfire regeneration of tree species that rely on ex situ seed sources (Table 1). We also expected that these effects would be greatest in hot and dry climate conditions likely to limit establishment because a greater seed supply would be needed to sustain at least some regeneration. We further expected that the effects of operational Fx zones would be limited to areas within species‐specific dispersal distances.

**TABLE 1:** Expectations for the effects of fire exclusion (Fx) zone amount and configuration on postfire tree regeneration in different climate scenarios (Q1) and for the effects of the operational Fx zone scenario on tree regeneration in burn patches and across the landscape (Q2).

Variable	Expectation	Rationale
Question 1—Potential Fx zones		
Fire exclusion zone	Postfire tree regeneration will be greater in Fx scenarios, but species responses will differ	Species that depend on ex situ propagules and are more likely to lose their climatic niches for establishment will benefit most
Amount	Postfire tree regeneration will increase with greater amounts	More seed sources are maintained on the landscape
Configuration	Dispersed configurations will result in more postfire tree regeneration compared to clumped configurations	Greater edge‐to‐area ratios and reduced distances to seed sources will maximize propagule supply to burned areas
Warm climate (RCP 4.5)	Postfire tree regeneration will increase weakly to moderately in response to Fx zones in scenarios with warm climate	Climate and fire activity will not change enough to compromise seed supply or tree establishment
Hot climate (RCP 8.5)	Postfire tree regeneration will increase strongly in response to Fx zones in scenarios with hot climate	Elevated fire activity will compromise seed supply outside Fx zones, and drought will further compromise establishment
Time	Effects of Fx zones will become more apparent by late century	Deviations from HRV in climate and fire activity will be greater
Question 2—Operational versus reference scenario		
Regeneration in burned patches	Postfire tree regeneration will be greater in the operational scenario, but species responses will differ	More seed sources are maintained on the landscape in the operational scenario
Regeneration across landscape	Postfire tree regeneration will be greater in the operational scenario, particularly near Fx zones	More seed sources are maintained on the landscape in the operational scenario
Climate	Hot, dry climate scenarios will result in greatest differences between operational and reference scenarios	Increased fire activity will compromise seed supply and drought will reduce germination and establishment

*Note*: For Q1, we compare Fx scenarios to each other and to a reference scenario without Fx zones, and for Q2 we compare operational and reference scenarios.

## METHODS

### Study area

**FIGURE 1:**
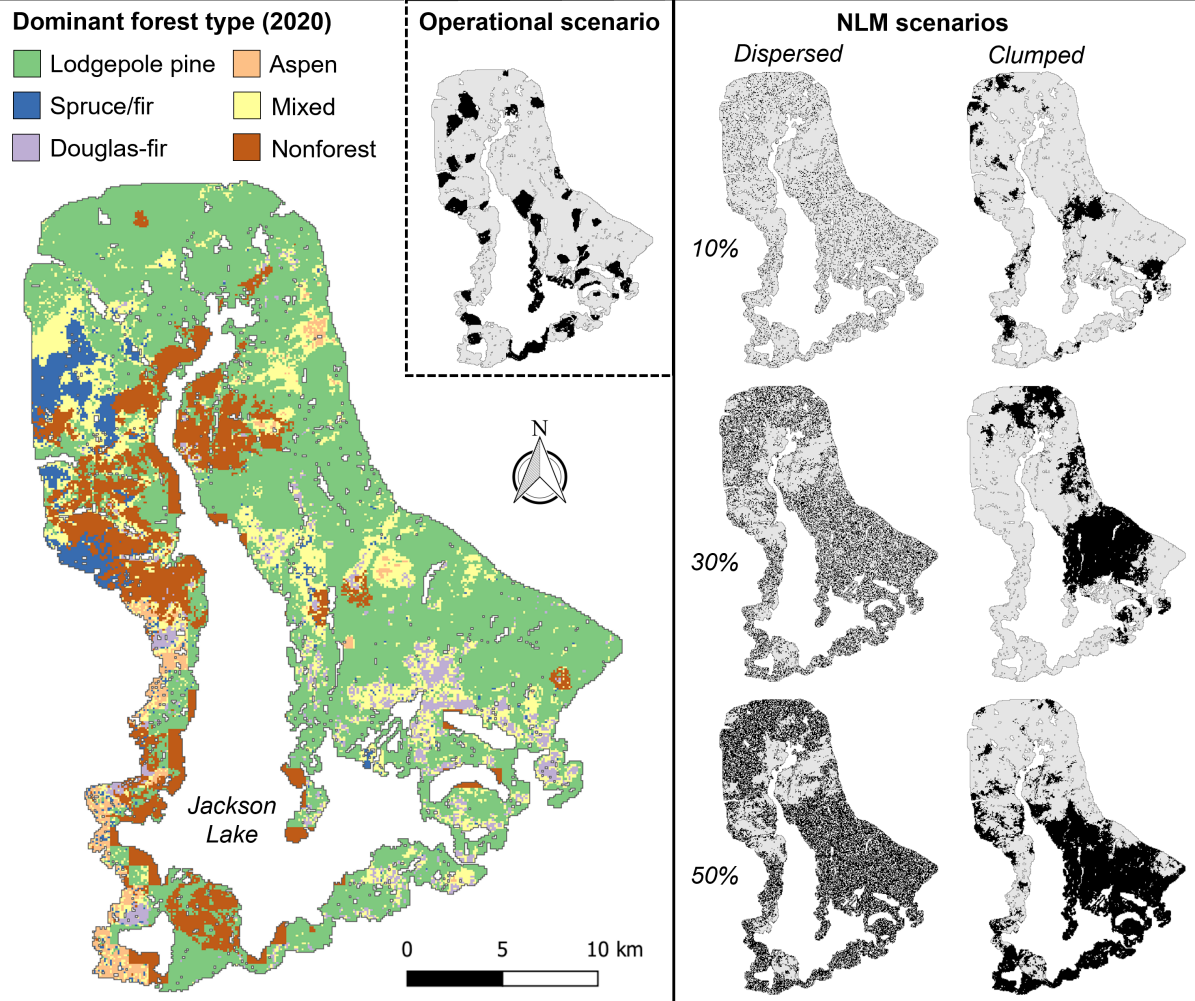
Dominant forest types in the iLand simulation landscape centered on Grand Teton National Park at the end of the model spin‐up (representing the year 2020; left) and the fire exclusion zone scenarios based on neutral landscape models (NLMs) with varying amounts and configurations (right). Upper inset panel shows the operational fire exclusion zone scenario delineated based on the presence of species vulnerable to novel fire regimes (subalpine fir, Engelmann spruce, and Douglas‐fir) and the potential for fire control through reasoned fire exclusion. Spatial patterns of the fire exclusion zones were imposed on the model landscape and simulated until 2100 under four climate scenarios.

Grand Teton National Park is located within the Greater Yellowstone Ecosystem (GYE) in northwestern Wyoming, USA. About 42% of the 130,000 ha encompassed by GRTE is forested (Hansen et al., 2020), with lower tree line around 1600 m above sea level (asl) and upper tree line above 2900 m asl (Clark, 1981; Iglesias et al., 2018). Our simulation landscape (Figure 1) follows the same boundaries as prior studies (e.g., Hansen et al., 2020; Turner et al., 2022) and encompasses 57,189 ha, of which 53,665 ha are potentially forested (i.e., stockable). The simulation landscape includes most forested areas of GRTE as well as portions of southern Yellowstone National Park, the Teton Wilderness on the Bridger‐Teton National Forest, and the Caribou‐Targhee National Forest. The average January temperature in GRTE is −10.8°C with an average July temperature of 15.3°C (30‐year climate normals, 1991–2020, for Moran Junction; NOAA, 2024). Precipitation occurs mainly in the winter as snowfall. Regional climate has shifted considerably since 1950, with warmer temperatures, drier summers and winters, and wetter springs and falls (Hostetler et al., 2021).

Forest composition in GRTE is shaped by climate, topography, and fire. Fires typically occur in summer and fall under hot, dry, and windy conditions (Loope & Gruell, 1973; Romme & Despain, 1989; Whitlock, 1993). Since 1972, fire in GRTE has been managed for resource benefit, and some naturally ignited fires are allowed to burn if they do not threaten lives or critical infrastructure. Mixed‐severity fire at intervals from 50 to 100 years (Loope & Gruell, 1973) is common in relatively warm and dry low‐elevation forests (Jacobs & Whitlock, 2008), favoring interior Douglas‐fir and quaking aspen. High‐severity, stand‐replacing fires at intervals from 100 to 300 years (Whitlock, 1993) are typical in mid‐elevation forests where lodgepole pine is dominant, especially on relatively flat terrain (Loope & Gruell, 1973). Fire intervals can exceed 300 years at higher elevations where mixed stands of Engelmann spruce, subalpine fir, and whitebark pine (*Pinus albicaulis*) occur near upper tree line (Loope & Gruell, 1973). Some spruce‐fir stands are also present at lower elevations in toe‐slope forests of the Teton Range.

### Model overview

We used iLand, an individual‐based forest landscape and disturbance model (Rammer et al., 2024; Seidl et al., 2012) that has been parameterized, well‐tested, and applied in the GYE (e.g., Braziunas et al., 2018; Hansen et al., 2020; Turner et al., 2022). Abiotic environmental conditions, including soil type, nutrients, and water availability, are defined at 100 m × 100 m resolution while light availability is calculated at 2 m × 2 m resolution in the simulations. iLand simulates growth, competition, and mortality of individual trees (>4 m in height) in response to abiotic drivers and based on first principles of ecology. Ecosystem processes are simulated hierarchically at multiple scales across spatially explicit landscapes (Rammer et al., 2024; Seidl et al., 2012). All regionally dominant conifer species are modeled using species‐specific parameters (Thom et al., 2024), with serotinous and non‐serotinous variants of lodgepole pine represented separately. In this study, we analyzed regeneration of wind‐dispersed conifers (subalpine fir, Engelmann spruce, Douglas‐fir, and lodgepole pine variants), but quaking aspen and whitebark pine were also simulated. For further details on iLand, see the model website (iLand, 2025).

Seed production by mature trees is modeled per m^2^ of crown area and modified based on species‐specific fecundity parameters, such as age of maturity and masting frequency. Seed dispersal is represented at 20 m × 20 m resolution using species‐specific probabilistic dispersal kernels around mature trees such that seed availability declines as distance from seed source increases. Focal cells receive seeds aggregated from surrounding kernels, and tree establishment then depends on environmental factors such as temperature, water, and light availability. Once established, regeneration cohorts are modeled at 2 m × 2 m resolution until a cohort reaches 4 m in height and graduates into the individual‐based model. If cohorts of multiple species establish within a cell, the species that first reaches the 4‐m height threshold wins the cell. We aggregated all model outputs to the scale of 100 m × 100 m grid cells for our analysis.

#### Climate scenarios

We selected two general circulation models (GCMs) with daily climate projections that differ in their expected future summer precipitation. While mean annual precipitation increases throughout the century in both GCMs, they project a wetter (CanESM2, Chylek et al., 2011) and a drier (HadGEM2‐ES, Collins et al., 2011) growing and fire season climate for our region. By the end of the 21st century, summer precipitation would increase by 1.5–3.3 cm in the wet GCM while it would decrease by 1.8–2.4 cm in the dry GCM. For each GCM, we included two relative concentration pathways (RCPs) that illustrate moderate warming (+4.1 to 4.2°C) with a stabilization in greenhouse gas concentrations by midcentury and radiative forcing increasing to 4.5 W m^−2^ (RCP 4.5, warm), or hotter temperatures (+6.6 to 6.8°C) with unabated emissions and radiative forcing increasing to 8.5 W m^−2^ (RCP 8.5, hot). We obtained downscaled climate projections at 4 km × 4 km resolution from Multivariate Adapted Constructed Analogs (MACA) datasets (Abatzoglou & Brown, 2012) and further downscaled these data to 100 m × 100 m resolution using relationships between climatic variables and lapse rates along elevational gradients (Dollinger et al., 2024).

#### Fire modeling

We imposed ignitions and maximum sizes of large fires (≥400 ha) based on statistical models of large fires in the GYE developed for the selected GCMs and adapted for iLand (Turner et al., 2022). Small fires (<400 ha) were simulated based on random ignitions. Fire spread was simulated dynamically at 20 m × 20 m resolution based on a cellular automaton approach with spread probabilities dependent on wind speed and direction, slope, as well as fuel amount and type (Hansen et al., 2020; Seidl et al., 2014). Fire effects are modeled for individual trees based on fuel amounts, average tree size, and bark thickness.

#### Model initialization

Forest composition and stand structure were initialized with a 310‐year spin‐up. The spin‐up was initialized with seedling cohorts matching contemporary forest composition and simulated for 310 years using climate years randomly drawn with replacement from CanESM2 RCP 4.5 for the 1950–2005 period. Starting at 1950 (spin‐up year 240), the actual climate year was used, and historical fire perimeters from the Monitoring Trends in Burn Severity (MTBS, Eidenshink et al., 2007) record (1984–2020) were imposed to reflect current stand ages in the model initialization. At the end of the spin‐up process (2020), the simulated landscape represented forest composition and structure in GRTE well (Dollinger et al., 2024; Hansen et al., 2020).

### Simulation experiments

We conducted two simulation experiments. To determine the effect of the amount and configuration of Fx zones on postfire tree regeneration (Q1), we varied the spatial configurations of forest delineated as Fx zones in a factorial simulation experiment using a NLM approach (Braziunas et al., 2021; Gardner et al., 1987; Gardner & Urban, 2007; Turner et al., 1989). To answer how Fx zones that prioritize operational firefighting considerations may affect tree regeneration within burned patches and across the landscape (Q2), we used insights on the spatial configuration of potential Fx zones from the NLM approach to delineate operational Fx zones along fuel breaks. We compared the results of each simulation experiment to a reference scenario without Fx zones under the respective climate scenario. For data processing and analyses, we used R Statistical Software (v4.3.2, R Core Team, 2023) with the NLMR and landscapetools (Sciaini et al., 2018), terra (Hijmans, 2023a), raster (Hijmans, 2023b), plotrix (Lemon, 2006), RSQLite (Müller et al., 2024), and Tidyverse (Wickham et al., 2019) packages.

#### Experiment 1: Amount and configuration of potential Fx zones

The factorial experiment varied the amount (10%, 30%, 50% of forested area) and configuration (dispersed or clumped) of potential Fx zones. This 3 × 2 factorial plus a reference scenario without Fx zones (seven scenarios) was simulated for 80 years (2021–2100) under all four climate scenarios and replicated within each combination to account for variation in fire histories (*n* = 20 replicates, for a total of 560 simulations). We generated NLMs of dispersed configurations by randomly selecting 1‐ha grid cells with probabilities corresponding to the three amounts (Figure 1). Clumped configurations were generated from fractional Brownian motion NLMs with a fractal dimension of 0.9 (i.e., strong spatial autocorrelation) that were then converted to a binary map corresponding to 10%, 30%, or 50% of potential Fx zones. The resulting six NLMs were clipped to the extent of the simulation landscape, and forest that overlapped with the NLMs was classified as Fx zones in each scenario (Figure 1).

The three amounts were selected because 10% aligns with the mean proportion of unburned patches within fire perimeters in the Northern Rockies from 1984 to 2014 (Figure 4 in Meddens et al., 2018) while 30% and 50% approximate connectivity (percolation) thresholds associated with 8‐neighbor (i.e., adjacent and diagonal neighbors of the same habitat are connected to a focal cell) and 4‐neighbor (i.e., only adjacent neighbors are connected to a focal cell) rules on random maps. As the occupied percent of the landscape increases, patch size increases, perimeter‐to‐area ratios decline, and cells become highly connected above the percolation threshold. Further, the 30% threshold also aligns with the proportion of unburned forests within perimeters of the 1988 Yellowstone fires (Turner et al., 1994).

In all potential Fx zone scenarios, we placed Fx zones in mature forests and excluded areas that had burned since 1984 (> 50% canopy mortality) to emphasize protection of live tree seed sources from burning. To model Fx zones in iLand, we adjusted fire spread probability so that fire could spread around but not in 100‐m × 100‐m grid cells designated as Fx zones. This 1‐ha minimum size of Fx zones corresponds with the mean size of unburned forest patches in fires of the Northern Rockies between 1984 and 2014 (Meddens et al., 2018).

To confirm that using a single NLM per amount × configuration scenario did not bias the results, we conducted a separate set of simulations with 10 unique NLMs of 30% Fx zones for both configurations and 10 unique fire histories in the hot‐dry climate scenario for a total of 200 additional simulations. These simulations showed minimal differences among unique NLM scenarios but some differences among unique fire histories (Appendix S1: Table S1, Figures S3 and S4).

We analyzed fire patterns and postfire tree regeneration across Fx scenarios for simulated fires that occurred early (2021–2045) and late (2071–2095) in the century. These time periods were analyzed separately because climate projections differ most from historical climate by late century. To verify the modeling of Fx zones and ensure Fx scenarios reduced fire, we computed mean area burned per year for both time periods per replicate as well as the mean and standard error (SE) across replicates for each climate scenario and time period (*n* = 20).

To answer our questions, we first recorded postfire tree regeneration by species within fire patches 5 years after a fire (including all burn severities). We then calculated the mean and SE of postfire tree regeneration per hectare early (2026–2050) and late (2076–2100) in the century. We chose the 5‐year postfire mark to account for interannual variation in weather and seed production and because pathways of postfire stand development in forests of the GYE lock in shortly after fire (Kashian et al., 2005; Turner et al., 1999). To confirm this choice did not bias our results, we separately analyzed regeneration at 15 years postfire, and trends were consistent at both times postfire (Appendix S1: Figures S5–S9). Our analysis included all burn severities because high‐severity fire, as defined by percent canopy mortality, becomes uncommon in some simulations during the late 21st century due to feedbacks of fires on forest structure. For more details on fire patterns in iLand simulations of 21st‐century forest and fire dynamics in the GYE, see Turner et al. (2022).

We used two statistical approaches to disentangle the effects of Fx zone amount and configuration and climate on postfire tree regeneration. First, we fit two‐way ANOVAs to mean postfire tree regeneration densities by species early (2026–2050) and late (2076–2100) in the century to assess the effects of the amount and configuration of Fx zones, excluding the reference scenario. Because future climate is uncertain, we conducted this analysis across all climate scenarios to detect the overall effect of amount and configuration. Second, we fit linear regression models (LR) with Fx scenario (amount + configuration) as a predictor of postfire tree regeneration by species and for each climate scenario early and late in the century to assess the effects of Fx zones in different climate scenarios. Postfire regeneration densities were transformed (log_10_ (density + 1)) prior to analysis to meet statistical model assumptions of linearity, normality of residual variance, and equal variance (Appendix S2: Figures S1–S6). Rather than relying on *p* values for the statistical significance of simulation results (White et al., 2014), we used model fit (adjusted *R* ^2^), *F* values (ANOVA), and *t* values (LR) to interpret the relative importance of Fx zones in explaining the variance of postfire tree regeneration at the two time periods.

#### Experiment 2: Operational Fx zones

Whereas experiment 1 explored the effects of the amount and configuration of Fx zones on postfire tree regeneration under future climate, many of those scenarios are not operationalizable. For example, it would not be possible for firefighters to defend a checkerboard of 50% dispersed Fx zones. Thus, we developed an “operational” Fx scenario that prioritized operational firefighting considerations (i.e., locations where reasoned fire exclusion may be possible) but incorporated ideal spatial configurations for postfire tree regeneration from experiment 1 wherever possible. We delineated operational Fx zones following fuel breaks around mature stands of vulnerable tree species in “as dispersed as possible” configurations targeting up to 30% of the landscape. Using the iLand spin‐up for 2020, we mapped areas with ≥50 trees ha^−1^ of species vulnerable to future loss of seed sources (subalpine fir, Engelmann spruce, and Douglas‐fir). We then delineated polygons of mature forest with at least a 20% likelihood of fire containment based on potential control locations (O'Connor et al., 2017) that could reasonably be protected from fire using fuel breaks and fire suppression tactics. The 20% threshold for potential control locations was selected because higher values (and thus a higher likelihood of successful fire exclusion by firefighters during a fire) are scarce in our landscape. We also included three developed areas designated for protection from wildfire by the park. These Fire Management Units are managed to limit 90% of unwanted fires to less than 4 ha in size (Grand Teton National Park, 2021). The resulting operational scenario consisted of 35 forest patches ranging from 55 to 972 ha (mean 275 ha) that accounted for 18% of the landscape (Figure 1).

We assessed the effects of the operational scenario on 21st‐century tree regeneration of subalpine fir, Engelmann spruce, and Douglas‐fir by two response variables. First, we calculated the mean and SE of postfire tree regeneration per hectare by species 5 years postfire within fire patches early and late in the century, as in question 1, and compared these densities to the reference scenario. Second, we calculated the mean regeneration density per hectare by species across the landscape, but outside of operational Fx zones, at the end of the simulation (2100) to assess landscape patterns of tree regeneration. We then computed the difference in mean tree regeneration by species in 2100 between the operational and reference scenarios for every 1‐ha cell in each climate scenario and mapped the log_10_ transformed difference across the landscape. We considered ±100 seedlings ha^−1^ as an ecologically meaningful difference because this corresponds to the mean postfire tree regeneration of subalpine fir, Engelmann spruce, and Douglas‐fir 5 years postfire across the GYE (Harvey et al., 2016). For cells with a negative difference (i.e., more regeneration in the reference scenario), the absolute value of the log_10_ difference was multiplied by −1 for mapping. Importantly, these maps depict the difference in regeneration between operational and reference scenarios by 2100 and not change over time. We then calculated the distance to protected seed sources (i.e., mean Euclidean distance to the operational Fx zones) of cells that had more, less, or no difference in regeneration in the operational relative to the reference scenario.

## RESULTS

**FIGURE 2:**
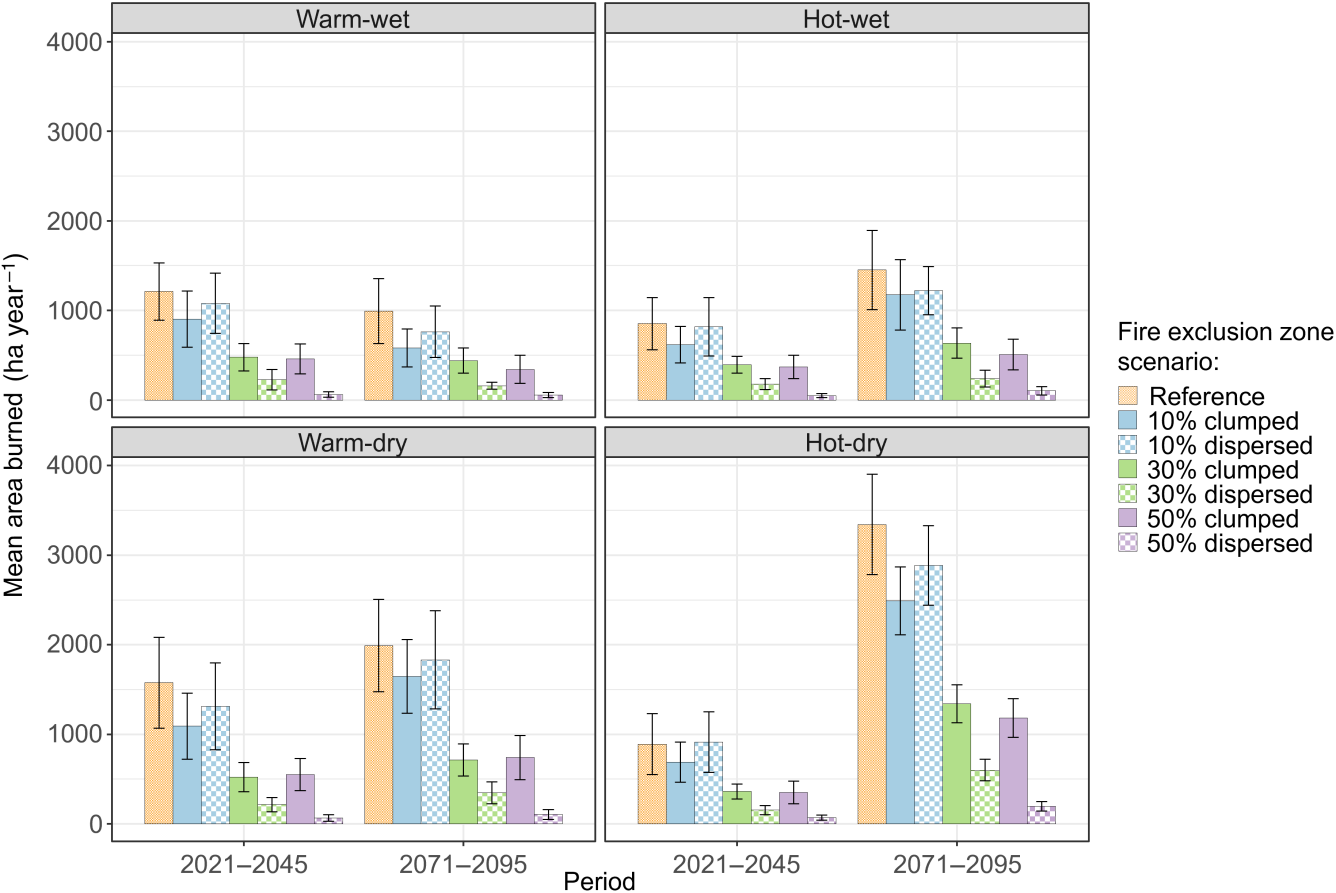
Mean annual area burned early and late in the 21st century for four climate scenarios in the reference and fire exclusion zone scenarios that vary in amount and configuration. Error bars represent ±2 SEs.

The 560 simulations under four climate scenarios and different amounts and configurations of Fx zones generated a wide range of area burned (48–3343 ha year^−1^) and postfire tree regeneration densities (21–7274 seedlings ha^−1^). Introduction of Fx zones in the GRTE landscape reduced area burned. In the reference scenario, mean area burned per year increased substantially late in the century with both hot (RCP 8.5) climate scenarios, peaking at an average of 3343 ha year^−1^ (± SE 280 ha year^−1^) in the hot‐dry scenario (Figure 2). In contrast, area burned was stable or even slightly declined through the century with both warm (RCP 4.5) climate scenarios, averaging 991 ha year^−1^ (±181 ha year^−1^) in wet (CanESM2) and 1990 ha year^−1^ (±257 ha year^−1^) in dry (HadGEM2‐ES) scenarios (Figure 2). Across climate scenarios, area burned declined with increasing amounts of Fx zones, and differences between configurations emerged with amounts ≥30%. For example, area burned with 50% Fx zones was six times lower in dispersed (195 ± 26 ha year^−1^) versus clumped (1182 ± 109 ha year^−1^) configuration in hot‐dry climate by late century (Figure 2).

### Question 1. Neutral landscape models

#### Amount and configuration of potential Fx zones

**FIGURE 3:**
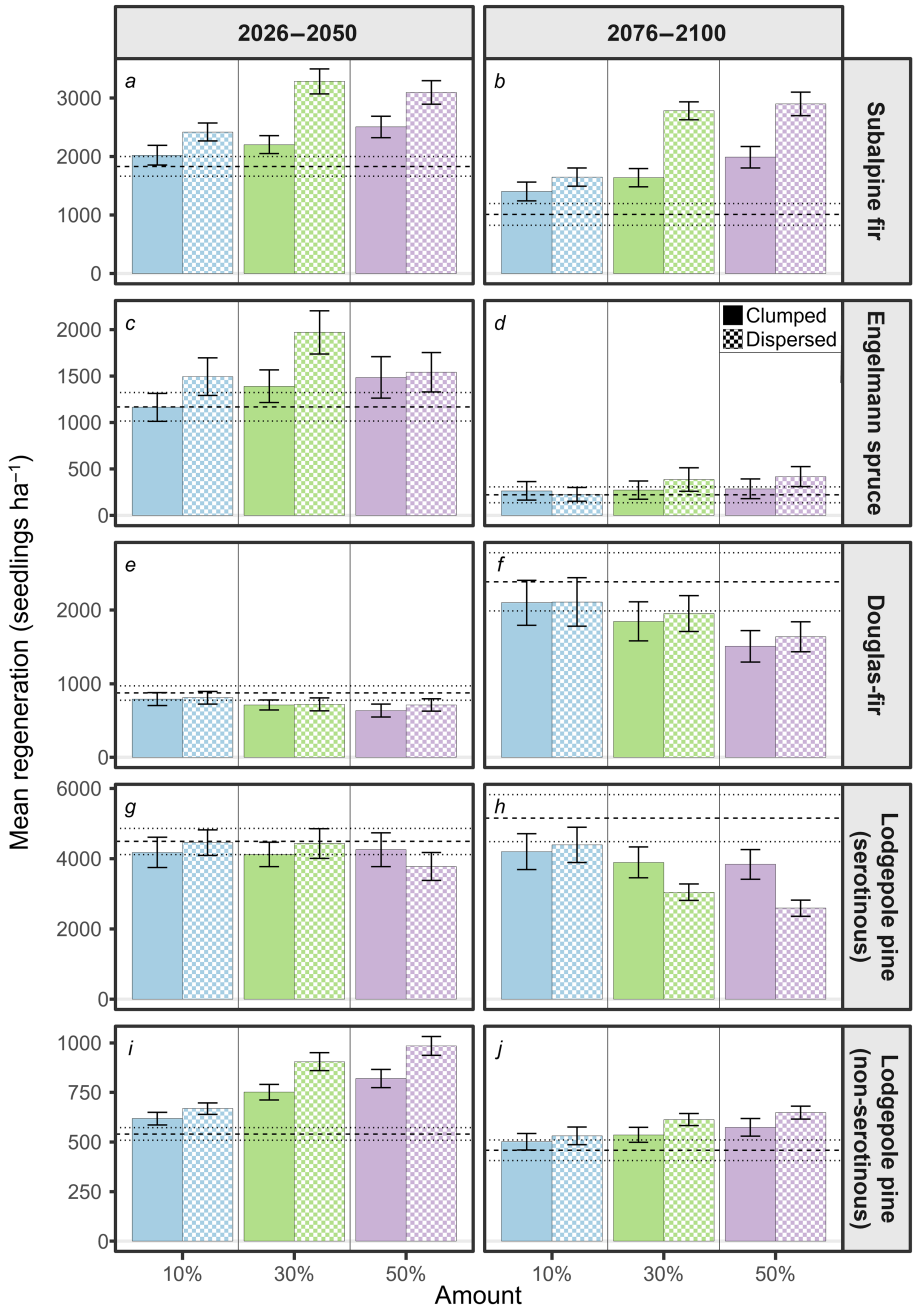
Mean postfire regeneration density in fire patches in response to different amounts and configuration of fire exclusion zones across all climate scenarios early (2026–2050) and late (2076–2100) in the century. Hashed bars represent dispersed configurations, and solid bars represent clumped configurations. Error bars represent ±2 SEs. Dashed line represents mean postfire regeneration of the reference scenario without fire exclusion zones, and dotted lines are ±2 SEs. Note that the range of *Y*‐axis differs for each species.

Across all climate scenarios, potential Fx zones increased postfire tree regeneration relative to reference scenarios for fire avoiders (subalpine fir, Engelmann spruce) and non‐serotinous lodgepole pine but decreased postfire tree regeneration for fire resisters (Douglas‐fir) and fire embracers (serotinous lodgepole pine; Figure 3). Postfire tree regeneration in Fx scenarios increased with amounts up to 30% of the landscape, with minimal differences between 30% and 50% of the landscape (Figure 3). When regeneration density increased in scenarios with Fx zones, regeneration was greater with dispersed versus clumped configurations (Figure 3, Appendix S2: Table S1). On average, regeneration densities of subalpine fir and Engelmann spruce were 46% and 25% higher (respectively) in dispersed relative to clumped configurations late in the century. Conversely, the amount of Fx zones mattered more than configuration for Douglas‐fir and serotinous lodgepole pine (Appendix S2: Table S1). Although the regeneration of both species increased over time, each 20% increase in the amount of Fx zones reduced the regeneration of Douglas‐fir and serotinous lodgepole pine by an average of 13% and 14%, respectively.

#### Postfire tree regeneration in Fx zone scenarios under alternative climate scenarios

**FIGURE 4:**
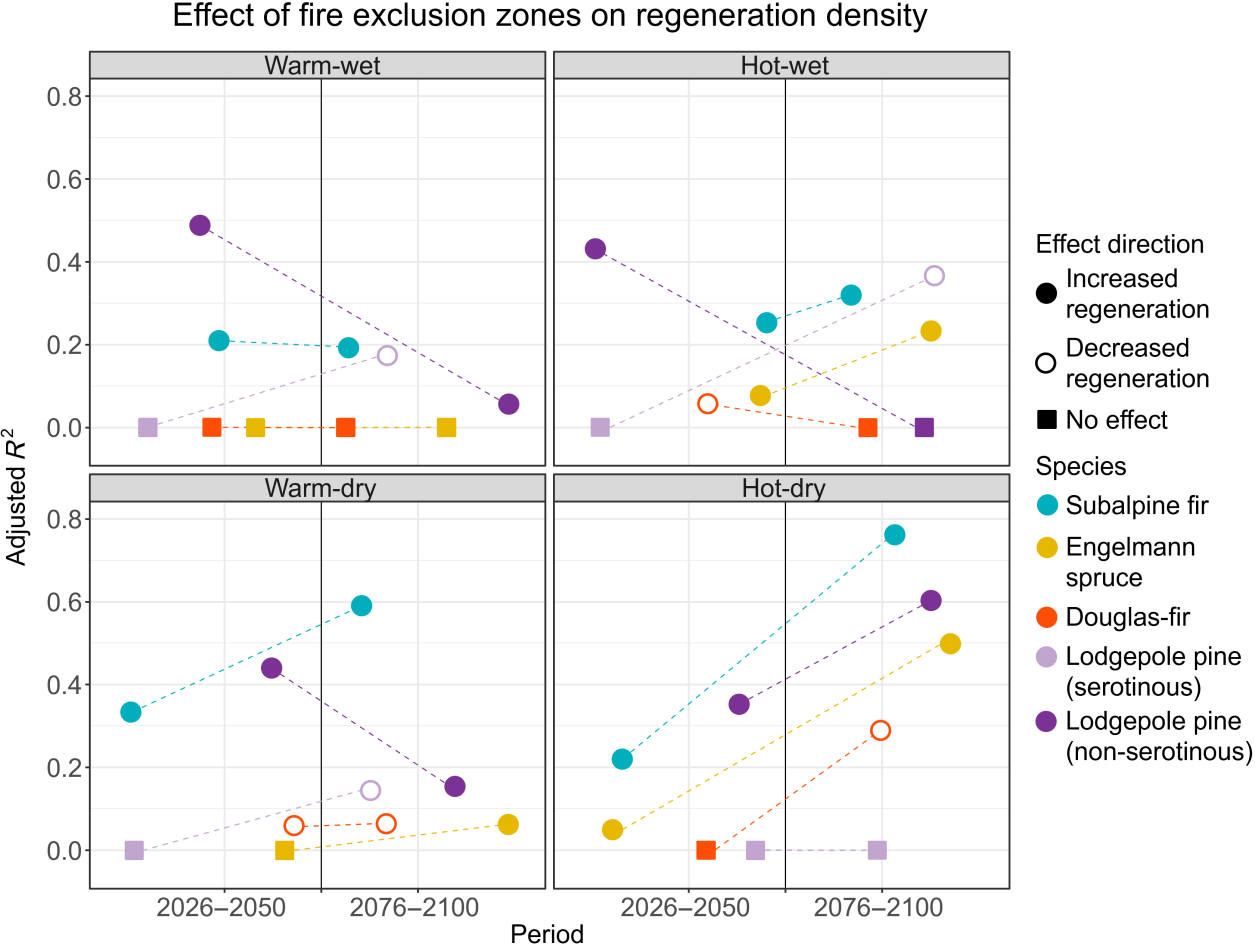
Variance (adjusted *R* ^2^) in postfire tree regeneration density explained by fire exclusion zones in the four climate scenarios early (2026–2050) and late (2076–2100) in the century. Filled dots represent an increase in postfire tree regeneration due to fire exclusion zones, and open dots represent a decrease (model *p* < 0.05).

The effects of Fx zones on postfire tree regeneration varied among climate scenarios and by species (Figure 4; Appendix S2: Tables S2 and S3). Among fire avoiders, subalpine fir regeneration was always greater in Fx relative to reference scenarios (Figure 3), but Fx zones were most important (i.e., explained most variation in regeneration between scenarios) with dry climates late in the century (warm‐dry adj. *R* ^2^ 0.59; hot‐dry adj. *R* ^2^ 0.76; Figure 4). Similarly, the importance of Fx zones for Engelmann spruce regeneration was strongest with hot climates late in the century (hot‐wet adj. *R* ^2^ 0.24; hot‐dry adj. *R* ^2^ 0.48) but minimal under warm climates and early in the century (Figure 4). Conversely, Fx zones explained more variation in non‐serotinous lodgepole pine regeneration early in the century in all climate scenarios except the hot‐dry scenario, when Fx zones were more important late in the century (early adj. *R* ^2^ 0.35, late adj. *R* ^2^ 0.60; Figure 4). Regeneration of serotinous variants was always unaffected early in the century but responded negatively to Fx zones by late century. Fx zones were less important for regeneration of serotinous lodgepole pine and Douglas‐fir, relative to the other conifers (all adj. *R* ^2^ < 0.40 and most <0.20; Figure 4).

### Experiment 2: Operational Fx zones

#### Postfire tree regeneration within fire patches

**FIGURE 5:**
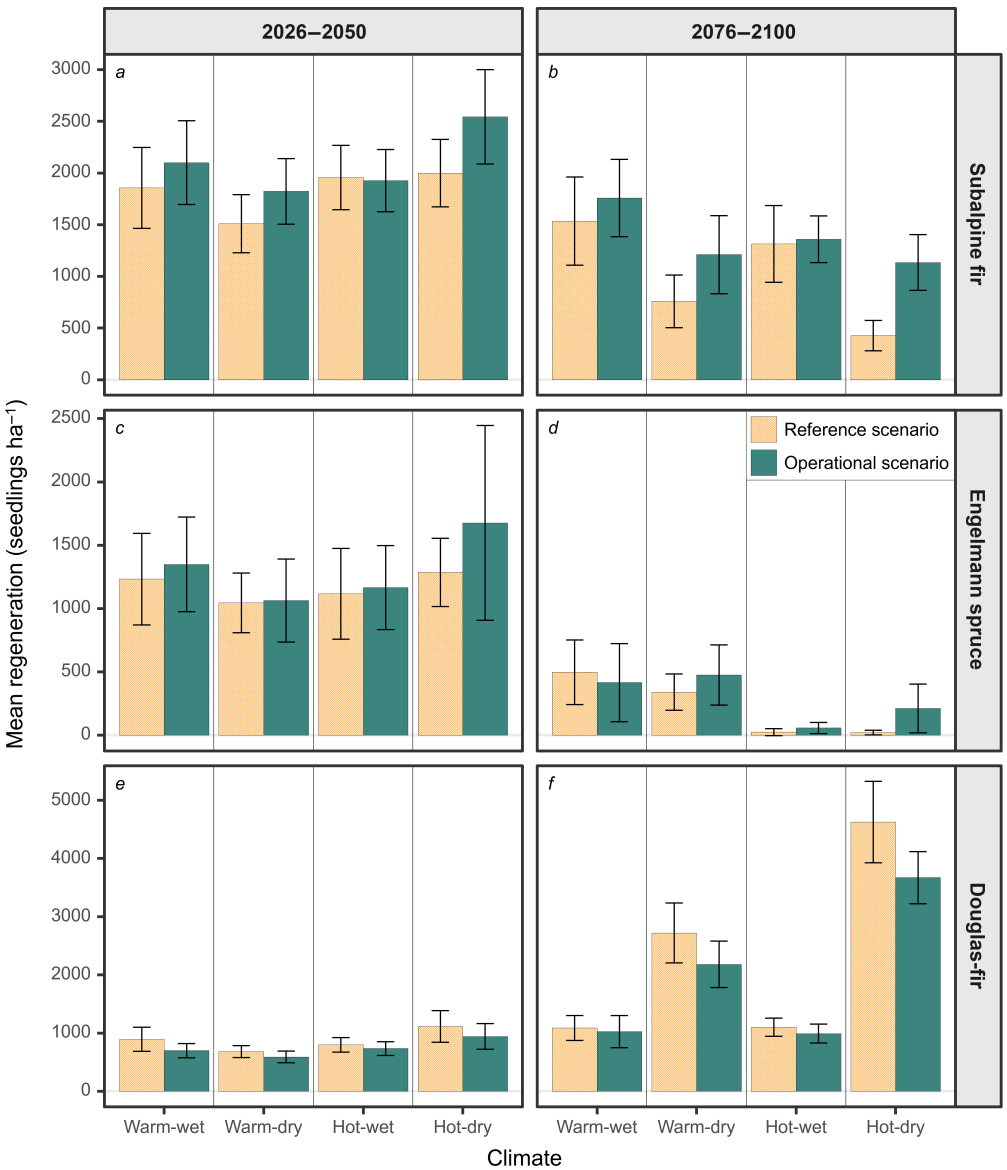
Subalpine fir, Engelmann spruce, and Douglas‐fir regeneration within fire patches in reference and operational fire exclusion zones scenarios early (2026–2050) and late (2076–2100) in the century. Error bars represent ±2 SEs. Note that the *Y*‐axis range differs between species.

Within fire patches, differences between operational and reference scenarios only emerged late in the century and with a hot‐dry climate (Figure 5). Subalpine fir declined over time in both scenarios, but late‐century regeneration was nearly three times greater in the operational versus reference scenario (1135 vs. 428 seedlings ha^−1^). Engelmann spruce regeneration also declined over time but was nearly 10 times greater by late century in the operational versus reference scenario (211 vs. 21 seedlings ha^−1^; Figure 5). In contrast, postfire regeneration of Douglas‐fir increased over time and did not differ between operational and reference scenarios (Figure 5).

#### Landscape effects of operational Fx zones on postfire tree regeneration by 2100

**FIGURE 6:**
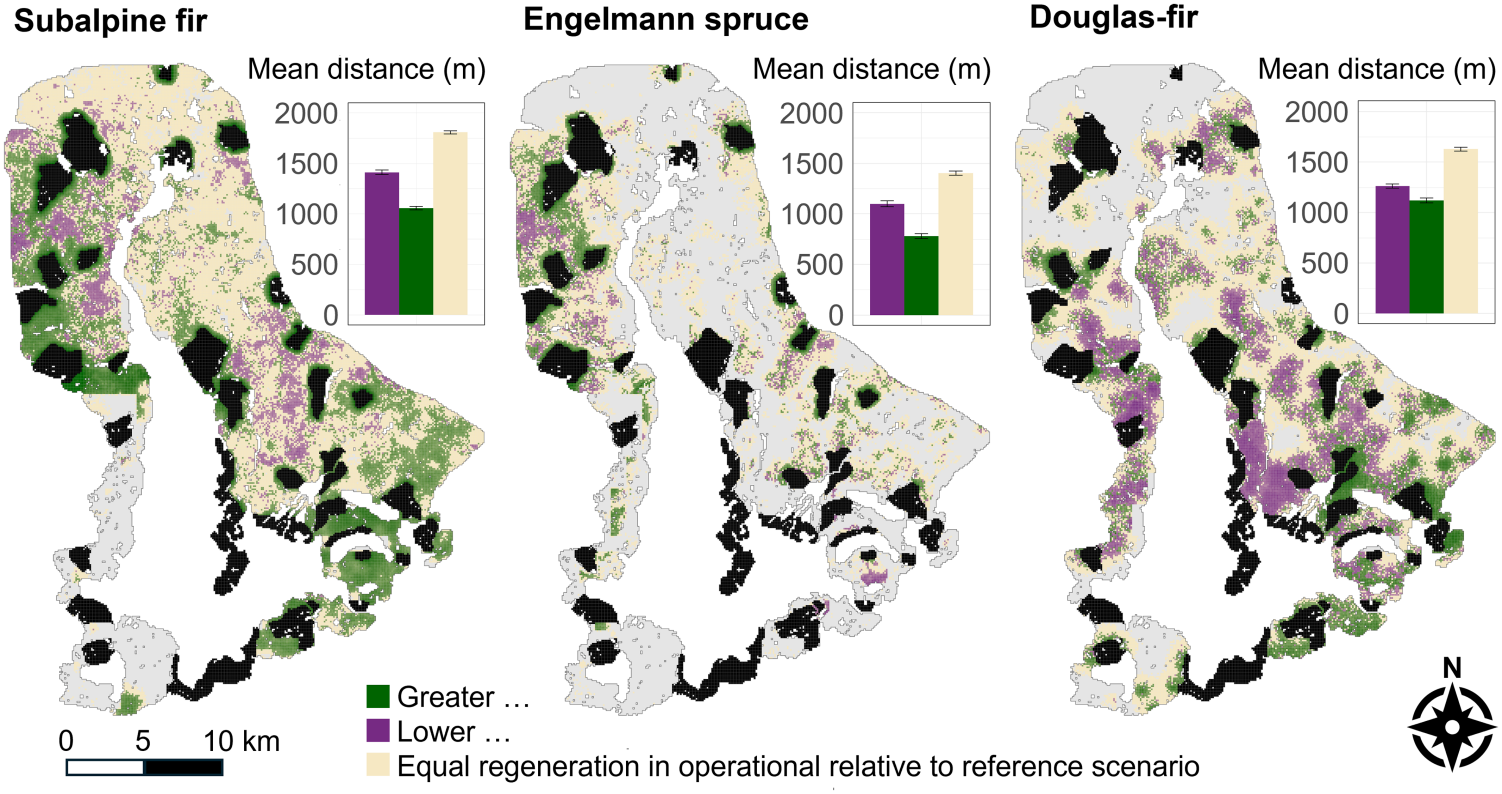
Spatial consequences of the operational fire exclusion zones scenario with hot‐dry climate by 2100. Purple areas contain fewer tree seedlings in the operational relative to the reference scenario in that cell across 20 replicates, while green areas contain more seedlings in the operational relative to the reference scenario. Beige shows no difference between the two scenarios (defined here as ±100 seedlings/ha), and gray is where the species was absent from both operational and reference scenarios. Black areas are operational fire exclusion zones. Absolute values of difference in regeneration densities were log_10_ transformed before mapping. Bar graph insets show mean distance to operational fire exclusion zones for cells with more, less, and no difference in regeneration densities.

The end‐of‐century difference between operational and reference scenarios identified areas where operational Fx zones enhanced or diminished tree seedling densities. Differences between scenarios were most pronounced in hot‐dry climate (Figure 6; Appendix S2: Figures S7 and S8), and increased tree seedling densities were clustered within ~1000 m from the edges of operational Fx zones (Figure 6). Subalpine fir and Engelmann spruce seedling densities generally increased near operational Fx zones in areas where mature trees were abundant initially (e.g., the northwest portion of the landscape; Figure 6). For example, Engelmann spruce regeneration was greater in the operational scenario in dry climate scenarios where spruce is currently more common (e.g., western portions of the landscape). There was little difference in regeneration densities between operational and reference scenarios as the distance from Fx zones increased (Figure 6).

## DISCUSSION

Our simulation experiments demonstrated that Fx zones influence postfire tree regeneration, especially later in the century and under dry, hot climate conditions that increasingly diverged from the past (Figures 3 and 5). Fx zones did not alter directional trends in postfire regeneration over time, but they lessened the declines of fire avoiders and the advances of fire resisters. Further, Fx scenarios reduced fire size and distance from burned areas to live tree seed sources, and these factors likely interacted to enhance regeneration of wind‐dispersed obligate seeders (Harvey et al., 2016; Niziolek et al., 2024). Our first experiment found that postfire tree regeneration was greatest with 30% of the landscape in dispersed rather than clumped configurations of Fx zones, consistent with findings from theoretical models of species dispersal in random and structured landscapes (King & With, 2002; Lavorel et al., 1994; With et al., 1997). In our second experiment, operational Fx zones enhanced regeneration of fire avoiders relative to the reference scenario in hot‐dry climate, and these benefits extended up to 1000 m from the edges of Fx zones. Thus, our study suggests that Fx zones have the potential to promote natural regeneration of vulnerable tree species (subalpine fir, Engelmann spruce) as climate and fire change throughout the 21st century.

### Amount and configuration of Fx zones

We expected greater postfire regeneration in scenarios with versus without Fx zones (Table 1), and this was supported for subalpine fir and Engelmann spruce. Both species are fire avoiders and particularly sensitive to loss of seed sources. Potential Fx zones had limited effects on lodgepole pine, which is fire sensitive but has capacity for both in situ and ex situ seed dispersal (Tinker et al., 1994). Douglas‐fir regeneration was largely unaffected by Fx zones but increased less during the 21st century in Fx relative to reference scenarios. Differential responses among species highlight the benefits of Fx zones for species more vulnerable to decline or loss. This finding is of management importance because it could guide interventions aimed at sustaining natural regeneration of at‐risk, fire‐avoiding species.

Our expectation that postfire tree regeneration would increase with greater amounts of Fx zones (Table 1) was partially supported, as benefits did not accrue beyond 30% of the landscape. In iLand, seeds disperse in all directions from a source cell, and the 30% reported here aligns closely with the percolation threshold associated with an 8‐neighbor rule on random maps (i.e., when spread is allowed to adjacent and diagonal neighbors of a focal cell; Gardner et al., 1987). Notably, this value is also close to the percentage of unburned forests (~28%) that remained within perimeters of the 1988 Yellowstone fires (Turner et al., 1994). Greater postfire regeneration associated with dispersed versus clumped configurations was consistent with our expectations (Table 1), because the greater perimeter‐to‐area ratios of small patches provide more opportunities for seeds to disperse from Fx zones into burned areas (Gustafson & Parker, 1992). Field studies have shown that numerous small live forest patches or individual trees enhance tree establishment in surrounding burned areas if seed source area is sufficient (Blomdahl et al., 2019; Coop et al., 2019). Larger high‐severity fire patches are projected with ongoing climate change (Parks & Abatzoglou, 2020), and establishing Fx zones could retain some of the heterogeneous mosaics of burn severity typical of crown‐fire systems that historically sustained postfire tree regeneration (Chappell & Agee, 1996; Turner et al., 1997).

The effects of Fx zones were stronger in hot climates (RCP 8.5) and later in the century for the two fire‐avoiding species, which followed our expectations (Table 1). Engelmann spruce historically regenerated well following fire (Doyle et al., 1998), but spruce regeneration is highly sensitive to temperature, particularly if moisture is also limiting (Hill et al., 2019; Knapp & Smith, 1982; Kueppers et al., 2017). The greater regeneration of spruce in Fx scenarios relative to reference scenarios suggests that Fx zones could maintain at least some spruce presence throughout the century. Declines in subalpine fir regeneration over time were less pronounced, and Fx zones further reduced those declines. Subalpine fir is less sensitive to warming temperatures than Engelmann spruce (Hart & Laroque, 2013; Peterson & Peterson, 1994) but disperses over shorter distances; thus, regeneration benefits from seed sources in close proximity to burned areas (Coop et al., 2010; McCaughey et al., 1986). The effects of Fx zones on subalpine fir regeneration were strongest in the dry climate scenarios, likely because Fx zones offset the loss of seed sources associated with greater area burned. This suggests that subalpine fir regeneration is more sensitive to changing disturbance than to changing climate (Perret et al., 2025). If spatial patterns of live forests that resemble historical postfire mosaics are retained on the landscape, regeneration of subalpine fir is likely to remain viable in future climates.

Lower Douglas‐fir regeneration in Fx scenarios was surprising, as other studies have demonstrated a clear influence of proximity to mature live seed sources (Donato et al., 2009; Harvey et al., 2016; Kemp et al., 2016). Although postfire Douglas‐fir regeneration is sensitive to high temperatures and drought (Hankin et al., 2019; Hoecker et al., 2020), it increased through time in our simulations. Cold temperatures historically limited the range of Douglas‐fir in GRTE, and our results suggest that future climate may be more suitable for Douglas‐fir (Turner et al., 2022). The range of Douglas‐fir in the GYE contracted over the past 500 years, likely due to decreases in precipitation (Iglesias et al., 2018; Whitlock et al., 2012). The increase in mean annual precipitation projected by both GCMs through the end of the century, despite differences in projected growing season precipitation, may favor Douglas‐fir expansion. Additional factors may have contributed to the slowing of Douglas‐fir expansion in our simulations with Fx zones. The reduction in area burned in Fx scenarios may have provided fewer opportunities for range expansions facilitated by fire (Hill & Field, 2021), and the current localized distribution of Douglas‐fir on the study landscape (Figure 1) could have further limited its ability to expand. Overall, we consider effects of Fx zones to be neutral for Douglas‐fir in this particular landscape, given the net increase in simulated regeneration of the species throughout the century.

### Operational Fx zones

The operational scenario did not halt or reverse trends in postfire regeneration densities by the end of the 21st century relative to the reference scenario, but it buffered the magnitude of ecological change in hot‐dry climate beyond species‐specific seed dispersal distances. Operational Fx zones had no substantial negative effects on regeneration of fire resisters and embracers, but ecologically meaningful benefits for fire avoiders. Although postfire regeneration densities of subalpine fir and Engelmann spruce always declined throughout the century, densities were three to tenfold greater by late century in operational scenarios with dry climate. This suggests the potential for a Fx management strategy to mitigate some climate‐driven losses in regeneration of fire‐avoiding species. On the landscape overall, operational Fx zones had pronounced effects on tree regeneration within a ~1000 m “zone of influence” around Fx zones. Notably, and counter to our expectations, the zone of influence extended beyond species‐specific seed dispersal distances (Braziunas et al., 2018; Thom et al., 2024). This could be due to Fx zones altering fire patterns in the surrounding landscape, or it may suggest that nucleation effects are at play over longer time periods.

Overall, our expectations for the operational scenario (Table 1) were largely supported. Variation in effects of operational Fx zones across the landscape suggests not only that current species distributions influence future distributions but also that the relative dominance of tree species will likely shift in the future, indicating forest reorganization (Seidl & Turner, 2022). Where to establish operational Fx zones should therefore be guided by current species composition and the likelihood that surrounding areas will remain suitable for regeneration (Larson et al., 2022). For example, locating Fx zones along elevational gradients could enhance upslope range expansions (Stueve et al., 2009) and help compensate for forest losses at lower elevations (Conlisk et al., 2017; Parks et al., 2019). Regeneration near tree line is particularly sensitive to climatic changes (Kueppers et al., 2017; Lazarus et al., 2018), and mature forests likely supported elevational shifts of tree species in GRTE throughout the Holocene (Iglesias et al., 2018). Although we did not account for microclimatic effects, climatic buffering of mature trees (Braziunas et al., 2025; Frey et al., 2016; Lutz et al., 2018) could facilitate upslope range expansions. Strategic delineation of Fx zones can retain vulnerable tree species (e.g., subalpine fir, Engelmann spruce) on the landscape without compromising regeneration of species better adapted to future climate and fire regimes (e.g., Douglas‐fir).

We also note that retaining mature forest patches in the landscape would support resource objectives beyond postfire tree regeneration (Downing et al., 2019; Sommers & Flannigan, 2022), including ecosystem services such as carbon storage (Kashian et al., 2013; Smithwick et al., 2009), recreation (Tanner et al., 2022), and wildlife habitat (Berry et al., 2015; Durkin et al., 2024; Steenvoorden et al., 2019). Fx zones may enhance future forest connectivity and facilitate species' ability to track suitable climatic niches (Balantic et al., 2021; Littlefield et al., 2019), which may be especially important given the limited ability of protected areas to facilitate species movement in response to climate change (Parks, Holsinger, Abatzoglou, et al., 2023). Further, Fx zones could also preserve niches for numerous shade‐tolerant, temperature‐sensitive understory plants and lichens associated with older forests (Hylander & Johnson, 2010; Kiel et al., 2023; Tucker & Kashian, 2018). If emissions are reduced and climate change significantly decreases during the 21st century, mature forest stands that persisted could initiate forest recovery as seeds disperse, new trees establish, and colonize over longer periods of time (i.e., nucleation, Corbin & Holl, 2012). Substantial management intervention will be required to retain mature subalpine forest stands on GYE landscapes throughout the 21st century, and managing high‐priority stands that sustain a variety of functions as Fx zones could maximize benefits.

### Management implications

Our study shows that *resisting* *some* change can affect pathways of forests adapted to infrequent, stand‐replacing fire relative to *accepting* *all* change associated with a warmer world with more fire. Our NLM approach (Q1 and experiment 1) showed that the most effective strategy was the one that most closely resembled historical postfire mosaics (30% live forest with an average patch size of 1 ha). This highlights that *resist* strategies can be effective if they are tailored to a system, address its unique vulnerabilities, and aim to replicate mechanisms that sustain resilience. Notably, establishing Fx zones through reasoned fire exclusion is distinct from a blanket resist approach, such as full fire suppression (Halofsky et al., 2018). Our approach accepts that fire regimes outside of Fx zones will change, but it may help avoid some state shifts to non‐forest.

Simultaneously, our operational scenario illustrates challenges with implementing management approaches that would be most beneficial to the ecosystem. Constraints in delineating defensible forest patches based on fuel breaks and topography resulted in an operational scenario that deviated from the optimal spatial pattern determined in experiment 1. Furthermore, establishing and maintaining operational Fx zones may be resource prohibitive given the cost and logistical challenges of wildland firefighting, and may not be a priority in a future with more fire and more people living in the wildland–urban interface (Radeloff et al., 2018). Whether intensive management is appropriate in protected areas, like national parks, is also subject to debate. Here, landscapes with multiple jurisdictions, like the GYE, may be advantageous because novel management strategies could be first implemented and experimentally tested on National Forest lands (Jackson, 2021).

Management plans that incorporate Fx zones could provide critical decision support to firefighting operations. Land management agencies prioritize critical values to protect during a fire, which can include valuable forest stands, and the Operations section of the Incident Management Team then develops strategies to protect those assets. For example, during the 2024 Pack Trail fire on the Bridger‐Teton National Forest adjacent to GRTE, fire was excluded from stands of whitebark pine (a threatened species under the Endangered Species Act) through point protection. Identification of these stands as high‐value assets before fire occurred enabled firefighters to reduce fuels ahead of the fire. Here, managers used reasoned fire exclusion to achieve management objectives, which suggests that implementation of Fx zones is possible.

### Caveats

Our study assessed postfire regeneration at five years postfire, and although this window of time is critical for recovery following high‐severity fire (Turner et al., 1997, 2004), it may not fully project the pathway of postfire cohorts as stands develop. We also assumed complete fire exclusion in Fx zones, but this could be difficult to achieve under extreme fire conditions; thus, managing static Fx zones may not be sustainable over the long term. Other management strategies, such as monitoring versus suppressing lightning‐caused fires when climatic conditions are not extreme (e.g., North et al., 2024), may also promote heterogeneity and complement a strategy of reasoned fire exclusion. Further, we only considered forests that are currently mature as Fx zones, yet protecting some young postfire forests that have the potential to mature and become a refuge could be valuable. And of course, any real‐world implementation of Fx zones must consider trade‐offs between the optimal spatial configuration to enhance postfire tree regeneration and the feasibility and costs of protecting these areas from fire. Before incorporating Fx zones in forest and fire management plans, managers must ask whether the ecological benefits warrant the investment in their system.

## CONCLUSIONS

Stewardship of national parks facing novel climatic conditions and fire regimes will not be easy (Gonzalez et al., 2018). Management already occurs in national parks (e.g., some fire suppression, hazard tree removal, invasive species control), but it remains unclear whether management to slow rates or patterns of forest change would be appropriate or effective. Yet, understanding the implications of 21st‐century climate change and the potential efficacy of management options is a critical first step toward decision‐making in an uncertain future (National Park Service, 2023). The National Cohesive Wildland Fire Management Strategy calls for managing wildfire for resilient landscapes (US Department of Interior and US Department of Agriculture, 2014), which requires clearly defined resource objectives prior to intervention. Here, we defined the resource objective as sustained tree regeneration and tested whether a *resist* management strategy in parts of the landscape could enhance forest resilience to fire using simulation modeling. Our approach of combining managerial and scientific expertise to first conduct a factorial experiment that laid out the cornerstones for an operational scenario could be a useful strategy for scenario planning in other systems. Fx zones preserved seed sources, suggesting that they could enhance natural regeneration of the fire‐sensitive obligate seeders most at risk of decline during the 21st century. Establishing and maintaining Fx zones early in the 21st century would maximize ecological benefits later in the century. Although Fx zones did not reverse directional trends in postfire tree regeneration over time, they demonstrated clear potential for gradual transitions to future forest states.

## CONFLICT OF INTEREST STATEMENT

The authors declare no conflicts of interest.

## Supporting information


Appendix S1.

Appendix S2.

## Data Availability

Data and code (Keller et al., 2025) are available in the Environmental Data Initiative Data Portal at https://doi.org/10.6073/pasta/57638b63563313ea39dbc2cd68b42add.

## References

[eap70121-bib-0002] Abatzoglou, J. T. , and A. P. Williams . 2016. “Impact of Anthropogenic Climate Change on Wildfire across Western US Forests.” Proceedings of the National Academy of Sciences of the United States of America 113(42): 11770–11775. 10.1073/pnas.1607171113. 27791053 PMC5081637

[eap70121-bib-0001] Abatzoglou, J. T. , and T. J. Brown . 2012. “A Comparison of Statistical Downscaling Methods Suited for Wildfire Applications.” International Journal of Climatology 32(5): 772–780. 10.1002/joc.2312.

[eap70121-bib-0003] Agee, J. K. 1993. “Fire Effects on Vegetation.” In Fire Ecology of Pacific Northwest Forests, Vol. 505. Washington, DC: Island Press.

[eap70121-bib-0004] Balantic, C. , A. Adams , S. Gross , R. Mazur , S. Sawyer , J. Tucker , M. Vernon , et al. 2021. “Toward Climate Change Refugia Conservation at an Ecoregion Scale.” Conservation Science and Practice 3(9): e497. 10.1111/csp2.497.

[eap70121-bib-0005] Berry, L. E. , D. B. Lindenmayer , and D. A. Driscoll . 2015. “Large Unburnt Areas, Not Small Unburnt Patches, Are Needed to Conserve Avian Diversity in Fire‐Prone Landscapes.” Journal of Applied Ecology 52(2): 486–495. 10.1111/1365-2664.12387.

[eap70121-bib-0006] Blomdahl, E. M. , C. A. Kolden , A. J. H. Meddens , and J. A. Lutz . 2019. “The Importance of Small Fire Refugia in the Central Sierra Nevada, California, USA.” Forest Ecology and Management 432(January): 1041–1052. 10.1016/j.foreco.2018.10.038.

[eap70121-bib-0009] Braziunas, K. H. , R. Seidl , W. Rammer , and M. G. Turner . 2021. “Can We Manage a Future with More Fire? Effectiveness of Defensible Space Treatment Depends on Housing Amount and Configuration.” Landscape Ecology 36(2): 309–330. 10.1007/s10980-020-01162-x.

[eap70121-bib-0007] Braziunas, K. H. , W. D. Hansen , R. Seidl , W. Rammer , and M. G. Turner . 2018. “Looking beyond the Mean: Drivers of Variability in Postfire Stand Development of Conifers in Greater Yellowstone.” Forest Ecology and Management 430(December): 460–471. 10.1016/j.foreco.2018.08.034. 35645456 PMC7612775

[eap70121-bib-0008] Braziunas, K. H. , W. Rammer , P. De Frenne , J. Díaz‐Calafat , P.‐O. Hedwall , C. Senf , D. Thom , F. Zellweger , and R. Seidl . 2025. “Microclimate Temperature Effects Propagate across Scales in Forest Ecosystems.” Landscape Ecology 40(2): 1–17. 10.1007/s10980-025-02054-8. 39912094 PMC11790809

[eap70121-bib-0010] Chappell, C. B. , and J. K. Agee . 1996. “Fire Severity and Tree Seedling Establishment in Abies Magnifica Forests, Southern Cascades, Oregon.” Ecological Applications 6(2): 628–640. 10.2307/2269397.

[eap70121-bib-0011] Chylek, P. , J. Li , M. K. Dubey , M. Wang , and G. Lesins . 2011. “Observed and Model Simulated 20th Century Arctic Temperature Variability: Canadian Earth System Model CanESM2.” Preprint. Aerosols/Atmospheric Modelling/Troposphere/Physics (Physical Properties and Processes). 10.5194/acpd-11-22893-2011.

[eap70121-bib-0013] Clark‐Wolf, K. , P. E. Higuera , B. N. Shuman , and K. K. McLauchlan . 2023. “Wildfire Activity in Northern Rocky Mountain Subalpine Forests Still within Millennial‐Scale Range of Variability.” Environmental Research Letters 18(9): 094029. 10.1088/1748-9326/acee16.

[eap70121-bib-0012] Clark, T. W. 1981. Ecology of Jackson Hole, Wyoming: A Primer, First ed. Salt Lake City, UT: Paragon Press.

[eap70121-bib-0014] Collins, W. J. , N. Bellouin , M. Doutriaux‐Boucher , N. Gedney , P. Halloran , T. Hinton , J. Hughes , et al. 2011. “Development and Evaluation of an Earth‐System Model – HadGEM2.” Geoscientific Model Development 4(4): 1051–1075. 10.5194/gmd-4-1051-2011.

[eap70121-bib-0015] Conlisk, E. , C. Castanha , M. J. Germino , T. T. Veblen , J. M. Smith , and L. M. Kueppers . 2017. “Declines in Low‐Elevation Subalpine Tree Populations Outpace Growth in High‐Elevation Populations with Warming.” Journal of Ecology 105(5): 1347–1357. 10.1111/1365-2745.12750.

[eap70121-bib-0017] Coop, J. D. , R. T. Massatti , and A. W. Schoettle . 2010. “Subalpine Vegetation Pattern Three Decades after Stand‐Replacing Fire: Effects of Landscape Context and Topography on Plant Community Composition, Tree Regeneration, and Diversity.” Journal of Vegetation Science 21(3): 472–487. 10.1111/j.1654-1103.2009.01154.x.

[eap70121-bib-0016] Coop, J. D. , T. J. DeLory , W. M. Downing , S. L. Haire , M. A. Krawchuk , C. Miller , M.‐A. Parisien , and R. B. Walker . 2019. “Contributions of Fire Refugia to Resilient Ponderosa Pine and Dry Mixed‐Conifer Forest Landscapes.” Ecosphere 10(7): e02809. 10.1002/ecs2.2809.

[eap70121-bib-0018] Corbin, J. D. , and K. D. Holl . 2012. “Applied Nucleation as a Forest Restoration Strategy.” Forest Ecology and Management 265: 37–46.

[eap70121-bib-0019] Davis, K. T. , S. Z. Dobrowski , P. E. Higuera , Z. A. Holden , T. T. Veblen , M. T. Rother , S. A. Parks , A. Sala , and M. P. Maneta . 2019. “Wildfires and Climate Change Push Low‐Elevation Forests across a Critical Climate Threshold for Tree Regeneration.” Proceedings of the National Academy of Sciences 116(13): 6193–6198. 10.1073/pnas.1815107116. PMC6442553 30858310

[eap70121-bib-0020] Dollinger, C. , W. Rammer , K. F. Suzuki , K. H. Braziunas , T. T. Keller , Y. Kobayashi , J. Mohr , A. S. Mori , M. G. Turner , and R. Seidl . 2024. “Beyond Resilience: Responses to Changing Climate and Disturbance Regimes in Temperate Forest Landscapes across the Northern Hemisphere.” Global Change Biology 30(8): e17468. 10.1111/gcb.17468. 39161313

[eap70121-bib-0021] Donato, D. C. , J. B. Fontaine , J. L. Campbell , W. Douglas Robinson , J. Boone Kauffman , and B. E. Law . 2009. “Conifer Regeneration in Stand‐Replacement Portions of a Large Mixed‐Severity Wildfire in the Klamath–Siskiyou Mountains.” Canadian Journal of Forest Research 39(4): 823–838. 10.1139/X09-016.

[eap70121-bib-0022] Downing, W. M. , M. A. Krawchuk , G. W. Meigs , S. L. Haire , J. D. Coop , R. B. Walker , E. Whitman , G. Chong , and C. Miller . 2019. “Influence of Fire Refugia Spatial Pattern on Post‐Fire Forest Recovery in Oregon's Blue Mountains.” Landscape Ecology 34(4): 771–792. 10.1007/s10980-019-00802-1.

[eap70121-bib-0023] Doyle, K. M. , D. H. Knight , D. L. Taylor , W. J. Barmore , and J. M. Benedict . 1998. “Seventeen Years of Forest Succession Following the Waterfalls Canyon Fire in Grand Teton National Park, Wyoming.” International Journal of Wildland Fire 8(1): 45. 10.1071/WF9980045.

[eap70121-bib-0024] Durkin, L. K. , P. D. Moloney , J. K. Cripps , J. L. Nelson , P. V. Macak , M. P. Scroggie , L. Collins , L. D. Emerson , J. Molloy , and L. F. Lumsden . 2024. “Unburnt Refugia Support Post‐Fire Population Recovery of a Threatened Arboreal Marsupial, Leadbeater's Possum.” Forest Ecology and Management 551(January): 121487. 10.1016/j.foreco.2023.121487.

[eap70121-bib-0025] Eidenshink, J. , B. Schwind , K. Brewer , Z.‐L. Zhu , B. Quayle , and S. Howard . 2007. “A Project for Monitoring Trends in Burn Severity.” Fire Ecology 3(1): 3–21. 10.4996/fireecology.0301003.

[eap70121-bib-0026] Frey, S. J. K. , A. S. Hadley , S. L. Johnson , M. Schulze , J. A. Jones , and M. G. Betts . 2016. “Spatial Models Reveal the Microclimatic Buffering Capacity of Old‐Growth Forests.” Science Advances 2(4): e1501392. 10.1126/sciadv.1501392. 27152339 PMC4846426

[eap70121-bib-0028] Gardner, R. H. , and D. L. Urban . 2007. “Neutral Models for Testing Landscape Hypotheses.” Landscape Ecology 22(1): 15–29. 10.1007/s10980-006-9011-4.

[eap70121-bib-0027] Gardner, R. H. , B. T. Milne , M. G. Turnei , and R. V. O'Neill . 1987. “Neutral Models for the Analysis of Broad‐Scale Landscape Pattern.” Landscape Ecology 1(1): 19–28. 10.1007/BF02275262.

[eap70121-bib-0029] Gill, N. S. , T. J. Hoecker , and M. G. Turner . 2021. “The Propagule Doesn't Fall Far from the Tree, Especially after Short‐Interval, High‐Severity Fire.” Ecology 102(1): e03194. 10.1002/ecy.3194. 32910502

[eap70121-bib-0030] Gonzalez, P. , F. Wang , M. Notaro , D. J. Vimont , and J. W. Williams . 2018. “Disproportionate Magnitude of Climate Change in United States National Parks.” Environmental Research Letters 13(10): 104001. 10.1088/1748-9326/aade09.

[eap70121-bib-0031] Grand Teton National Park . 2021. “Fire Management Plan ‐ Grand Teton National Park and the John D. Rockefeller, Jr. Memorial Parkway.” US Department of the Interior. https://gacc.nifc.gov/gbcc/dispatch/wy-tdc/home/sites/default/files/site-files/GTNP_Fire_Management_Plan_2021_signed_508.pdf.

[eap70121-bib-0032] Gustafson, E. J. , and G. R. Parker . 1992. “Relationships between Landcover Proportion and Indices of Landscape Spatial Pattern.” Landscape Ecology 7(2): 101–110. 10.1007/BF02418941.

[eap70121-bib-0033] Halofsky, J. S. , D. C. Donato , J. F. Franklin , J. E. Halofsky , D. L. Peterson , and B. J. Harvey . 2018. “The Nature of the Beast: Examining Climate Adaptation Options in Forests with Stand‐Replacing Fire Regimes.” Ecosphere 9(3): e02140. 10.1002/ecs2.2140.

[eap70121-bib-0034] Hankin, L. E. , P. E. Higuera , K. T. Davis , and S. Z. Dobrowski . 2019. “Impacts of Growing‐Season Climate on Tree Growth and Post‐Fire Regeneration in Ponderosa Pine and Douglas‐Fir Forests.” Ecosphere 10(4): e02679. 10.1002/ecs2.2679.

[eap70121-bib-0035] Hansen, W. D. , D. Abendroth , W. Rammer , R. Seidl , and M. G. Turner . 2020. “Can Wildland Fire Management Alter 21st‐Century Subalpine Fire and Forests in Grand Teton National Park, Wyoming, USA?” Ecological Applications 30(2): e02030. 10.1002/eap.2030. 31674698 PMC7612770

[eap70121-bib-0036] Hart, S. J. , and C. P. Laroque . 2013. “Searching for Thresholds in Climate–Radial Growth Relationships of Engelmann Spruce and Subalpine Fir, Jasper National Park, Alberta, Canada.” Dendrochronologia 31(1): 9–15. 10.1016/j.dendro.2012.04.005.

[eap70121-bib-0037] Harvey, B. J. , D. C. Donato , and M. G. Turner . 2016. “High and Dry: Post‐Fire Tree Seedling Establishment in Subalpine Forests Decreases with Post‐Fire Drought and Large Stand‐Replacing Burn Patches.” Global Ecology and Biogeography 25(6): 655–669. 10.1111/geb.12443.

[eap70121-bib-0123] Hijmans, R. 2023a. “Terra: Spatial Data Analysis.” R package version 1: 7–23. https://CRAN.R-project.org/package=terra.

[eap70121-bib-0124] Hijmans, R. 2023b. “Raster: Geographic Data Analysis and Modeling.” R package version 3: 6–20. https://CRAN.R-project.org/package=raster.

[eap70121-bib-0038] Hill, A. P. , and C. B. Field . 2021. “Forest Fires and Climate‐Induced Tree Range Shifts in the Western US.” Nature Communications 12(1): 6583. 10.1038/s41467-021-26838-z. PMC8594433 34782624

[eap70121-bib-0039] Hill, E. , S. Ex , C. Aldridge , and C. Prolic . 2019. “Overstory Density, Short Growing Seasons, and Moisture Limit Engelmann Spruce Establishment over Time in High‐Elevation Managed Stands.” Canadian Journal of Forest Research 49(1): 64–75. 10.1139/cjfr-2018-0268.

[eap70121-bib-0040] Hoecker, T. J. , W. D. Hansen , and M. G. Turner . 2020. “Topographic Position Amplifies Consequences of Short‐Interval Stand‐Replacing Fires on Postfire Tree Establishment in Subalpine Conifer Forests.” Forest Ecology and Management 478(December): 118523. 10.1016/j.foreco.2020.118523.

[eap70121-bib-0041] Holling, C. S. 1973. “Resilience and Stability of Ecological Systems.” Annual Review of Ecology and Systematics 4: 1–23.

[eap70121-bib-0042] Hostetler, S. , C. Whitlock , B. Shuman , D. Liefert , C. W. Drimal , and S. Bischke . 2021. “Greater Yellowstone Climate Assessment: Past, Present, and Future Climate Change in Greater Yellowstone Watersheds.” Technical Report. Bozeman, MT: Montana State University, Institute on Ecosystems. 10.15788/GYCA2021.

[eap70121-bib-0043] Hylander, K. , and S. Johnson . 2010. “In Situ Survival of Forest Bryophytes in Small‐Scale Refugia after an Intense Forest Fire: In Situ Survival of Forest Bryophytes after Intense Fire.” Journal of Vegetation Science 21(6): 1099–1109. 10.1111/j.1654-1103.2010.01220.x.

[eap70121-bib-0044] Iglesias, V. , C. Whitlock , T. R. Krause , and R. G. Baker . 2018. “Past Vegetation Dynamics in the Yellowstone Region Highlight the Vulnerability of Mountain Systems to Climate Change.” Journal of Biogeography 45(8): 1768–1780. 10.1111/jbi.13364.

[eap70121-bib-0121] iLand . 2025. “iLand ‐ The Individual‐Based Forest Landscape and Disturbance Model.” https://iland-model.org/.

[eap70121-bib-0046] Jackson, S. T. 2021. “Transformational Ecology and Climate Change.” Science 373(6559): 1085–1086. 10.1126/science.abj6777. 34516851

[eap70121-bib-0047] Jacobs, K. , and C. Whitlock . 2008. “A 2000‐Year Environmental History of Jackson Hole, Wyoming, Inferred from Lake‐Sediment Records.” Western North American Naturalist 68(3): 350–364. 10.3398/1527-0904(2008)68[350:AYEHOJ]2.0.CO;2.

[eap70121-bib-0049] Kashian, D. M. , M. G. Turner , W. H. Romme , and C. G. Lorimer . 2005. “Variability and Convergence in Stand Structural Development on a Fire‐Dominated Subalpine Landscape.” Ecology 86(3): 643–654. 10.1890/03-0828.

[eap70121-bib-0048] Kashian, D. M. , W. H. Romme , D. B. Tinker , M. G. Turner , and M. G. Ryan . 2013. “Postfire Changes in Forest Carbon Storage over a 300‐Year Chronosequence of Pinus Contorta‐Dominated Forests.” Ecological Monographs 83(1): 49–66. 10.1890/11-1454.1.

[eap70121-bib-0050] Keeley, J. E. 2012. “Ecology and Evolution of Pine Life Histories.” Annals of Forest Science 69(4): 445–453. 10.1007/s13595-012-0201-8.

[eap70121-bib-0051] Keeley, J. E. , G. Ne'eman , and C. J. Fotheringham . 1999. “Immaturity Risk in a Fire‐Dependent Pine.” Journal of Mediterranean Ecology 1: 41–48.

[eap70121-bib-0052] Keller, T. T. , D. C. Abendroth , P. R. Hood , K. H. Braziunas , C. Dollinger , R. Seidl , G. J. Knowlton , and M. G. Turner . 2025. “Data for: Can Fire Exclusion Zones Enhance Postfire Tree Regeneration? A Simulation Study in Subalpine Conifer Forests ver 1.” Environmental Data Initiative. 10.6073/pasta/57638b63563313ea39dbc2cd68b42add. PMC12529467 41099191

[eap70121-bib-0053] Kemp, K. B. , P. E. Higuera , and P. Morgan . 2016. “Fire Legacies Impact Conifer Regeneration across Environmental Gradients in the U.S. Northern Rockies.” Landscape Ecology 31(3): 619–636. 10.1007/s10980-015-0268-3.

[eap70121-bib-0054] Kemp, K. B. , P. E. Higuera , P. Morgan , and J. T. Abatzoglou . 2019. “Climate Will Increasingly Determine Post‐Fire Tree Regeneration Success in Low‐Elevation Forests, Northern Rockies, USA.” Ecosphere 10(1): e02568. 10.1002/ecs2.2568.

[eap70121-bib-0055] Kiel, N. G. , K. H. Braziunas , and M. G. Turner . 2023. “Peeking under the Canopy: Anomalously Short Fire‐Return Intervals Alter Subalpine Forest Understory Plant Communities.” New Phytologist 239(4): 1225–1238. 10.1111/nph.19009. 37259635

[eap70121-bib-0056] King, A. W. , and K. A. With . 2002. “Dispersal Success on Spatially Structured Landscapes: When Do Spatial Pattern and Dispersal Behavior Really Matter?” Ecological Modelling 147(1): 23–39.

[eap70121-bib-0057] Kirchhoff, C. J. , M. C. Lemos , and S. Dessai . 2013. “Actionable Knowledge for Environmental Decision Making: Broadening the Usability of Climate Science.” Annual Review of Environment and Resources 38: 393–414. 10.1146/annurev-environ-022112-112828.

[eap70121-bib-0058] Knapp, A. K. , and W. K. Smith . 1982. “Factors Influencing Understory Seedling Establishment of Engelmann Spruce (*Picea engelmannii*) and Subalpine Fir (*Abies lasiocarpa*) in Southeast Wyoming.” Canadian Journal of Botany 60(12): 2753–2761. 10.1139/b82-337.

[eap70121-bib-0059] Kueppers, L. M. , E. Conlisk , C. Castanha , A. B. Moyes , M. J. Germino , P. de Valpine , M. S. Torn , and J. B. Mitton . 2017. “Warming and Provenance Limit Tree Recruitment across and beyond the Elevation Range of Subalpine Forest.” Global Change Biology 23(6): 2383–2395. 10.1111/gcb.13561. 27976819

[eap70121-bib-0060] Larson, A. J. , S. M. A. Jeronimo , P. F. Hessburg , J. A. Lutz , N. A. Povak , C. A. Cansler , V. R. Kane , and D. J. Churchill . 2022. “Tamm Review: Ecological Principles to Guide Post‐Fire Forest Landscape Management in the Inland Pacific and Northern Rocky Mountain Regions.” Forest Ecology and Management 504(January): 119680. 10.1016/j.foreco.2021.119680.

[eap70121-bib-0061] Lavorel, S. , R. V. O'Neill , and R. H. Gardner . 1994. “Spatio‐Temporal Dispersal Strategies and Annual Plant Species Coexistence in a Structured Landscape.” Oikos 71(1): 75–88. 10.2307/3546174.

[eap70121-bib-0062] Lazarus, B. E. , C. Castanha , M. J. Germino , L. M. Kueppers , and A. B. Moyes . 2018. “Growth Strategies and Threshold Responses to Water Deficit Modulate Effects of Warming on Tree Seedlings from Forest to Alpine.” Journal of Ecology 106(2): 571–585. 10.1111/1365-2745.12837.

[eap70121-bib-0063] Lecina‐Diaz, J. , J. Martínez‐Vilalta , A. Alvarez , M. Banqué , J. Birkmann , D. Feldmeyer , J. Vayreda , and J. Retana . 2021. “Characterizing Forest Vulnerability and Risk to Climate‐Change Hazards.” Frontiers in Ecology and the Environment 19(2): 126–133. 10.1002/fee.2278.

[eap70121-bib-0125] Lemon, J. 2006. “Plotrix: A Package in the Red Light District of R.” R‐News 6(4): 8–12.

[eap70121-bib-0064] Littlefield, C. E. , M. Krosby , J. L. Michalak , and J. J. Lawler . 2019. “Connectivity for Species on the Move: Supporting Climate‐Driven Range Shifts.” Frontiers in Ecology and the Environment 17(5): 270–278. 10.1002/fee.2043.

[eap70121-bib-0065] Loope, L. L. , and G. E. Gruell . 1973. “The Ecological Role of Fire in the Jackson Hole Area, Northwestern Wyoming.” Quaternary Research 3(3): 425–443. 10.1016/0033-5894(73)90007-0.

[eap70121-bib-0066] Lutz, J. A. , T. J. Furniss , D. J. Johnson , S. J. Davies , D. Allen , A. Alonso , K. J. Anderson‐Teixeira , et al. 2018. “Global Importance of Large‐Diameter Trees.” Global Ecology and Biogeography 27(7): 849–864. 10.1111/geb.12747.

[eap70121-bib-0067] Lynch, A. J. , L. M. Thompson , E. A. Beever , D. N. Cole , A. C. Engman , C. H. Hoffman , S. T. Jackson , et al. 2021. “Managing for RADical Ecosystem Change: Applying the Resist‐Accept‐Direct (RAD) Framework.” Frontiers in Ecology and the Environment 19(8): 461–469. 10.1002/fee.2377.

[eap70121-bib-0068] Mackey, B. , D. Lindenmayer , P. Norman , C. Taylor , and S. Gould . 2021. “Are Fire Refugia less Predictable Due to Climate Change?” Environmental Research Letters 16(11): 114028. 10.1088/1748-9326/ac2e88.

[eap70121-bib-0069] McCaughey, W. W. , W. C. Schmidt , and R. C. Shearer . 1986. “Seed‐Dispersal Characteristics of Conifers in the Inland Mountain West.” USDA Forest Service General Technical Report INT‐Intermountain Forest and Range Experiment Station (USA), no. 203.

[eap70121-bib-0070] Meddens, A. J. H. , C. A. Kolden , J. A. Lutz , J. T. Abatzoglou , and A. T. Hudak . 2018. “Spatiotemporal Patterns of Unburned Areas within Fire Perimeters in the Northwestern United States from 1984 to 2014.” Ecosphere 9(2): e02029. 10.1002/ecs2.2029.

[eap70121-bib-0071] Meigs, G. W. , C. J. Dunn , S. A. Parks , and M. A. Krawchuk . 2020. “Influence of Topography and Fuels on Fire Refugia Probability under Varying Fire Weather Conditions in Forests of the Pacific Northwest, USA.” Canadian Journal of Forest Research 50(7): 636–647. 10.1139/cjfr-2019-0406.

[eap70121-bib-0072] Millspaugh, S. H. , C. Whitlock , and P. J. Bartlein . 2000. “Variations in Fire Frequency and Climate over the Past 17 000 Yr in Central Yellowstone National Park.” Geology 28(3): 211–214.

[eap70121-bib-0073] Morgan, P. , G. H. Aplet , J. B. Haufler , H. C. Humphries , M. M. Moore , and W. D. Wilson . 1994. “Historical Range of Variability: A Useful Tool for Evaluating Ecosystem Change.” Journal of Sustainable Forestry 2(1–2): 87–111. 10.1300/J091v02n01_04.

[eap70121-bib-0126] Müller, K. , H. Wickham , D. A. James , and S. Falcon . 2024. “RSQLite: SQLite Interface for R.” R package version 2(3): 7 https://github.com/r-dbi/rsqlite.

[eap70121-bib-0074] National Park Service . 2023. National Park Service Climate Change Response Strategy 2023 Update. Washington, DC: National Park Service.

[eap70121-bib-0075] Niziolek, D. , L. B. Harris , and A. H. Taylor . 2024. “Forest Resilience and Post‐Fire Conifer Regeneration in the Southern Cascades, Lassen Volcanic National Park California, USA.” Forest Ecology and Management 561(June): 121848. 10.1016/j.foreco.2024.121848.

[eap70121-bib-0076] NOAA . 2024. “NOAA NCEI U.S. Climate Normals Quick Access.” U.S. Climate Normals. https://www.ncei.noaa.gov/access/us-climate-normals/#dataset=normals-monthly&timeframe=30&location=WY&station=USC00486440

[eap70121-bib-0077] North, M. P. , S. M. Bisbing , D. L. Hankins , P. F. Hessburg , M. D. Hurteau , L. N. Kobziar , M. D. Meyer , A. E. Rhea , S. L. Stephens , and C. S. Stevens‐Rumann . 2024. “Strategic Fire Zones Are Essential to Wildfire Risk Reduction in the Western United States.” Fire Ecology 20(1): 50. 10.1186/s42408-024-00282-y.

[eap70121-bib-0078] O'Connor, C. D. , D. E. Calkin , M. P. Thompson , C. D. O' Connor , D. E. Calkin , and M. P. Thompson . 2017. “An Empirical Machine Learning Method for Predicting Potential Fire Control Locations for Pre‐Fire Planning and Operational Fire Management.” International Journal of Wildland Fire 26(7): 587–597. 10.1071/WF16135.

[eap70121-bib-0079] Overpeck, J. T. , D. Rind , and R. Goldberg . 1990. “Climate‐Induced Changes in Forest Disturbance and Vegetation.” Nature 343(6253): 51–53. 10.1038/343051a0.

[eap70121-bib-0080] Parks, S. A. , and J. T. Abatzoglou . 2020. “Warmer and Drier Fire Seasons Contribute to Increases in Area Burned at High Severity in Western US Forests from 1985 to 2017.” Geophysical Research Letters 47(22): e2020GL089858. 10.1029/2020GL089858.

[eap70121-bib-0082] Parks, S. A. , L. M. Holsinger , J. T. Abatzoglou , C. E. Littlefield , and K. A. Zeller . 2023. “Protected Areas Not Likely to Serve as Steppingstones for Species Undergoing Climate‐Induced Range Shifts.” Global Change Biology 29(10): 2681–2696. 10.1111/gcb.16629. 36880282

[eap70121-bib-0083] Parks, S. A. , L. M. Holsinger , K. Blankenship , G. K. Dillon , S. A. Goeking , and R. Swaty . 2023. “Contemporary Wildfires Are More Severe Compared to the Historical Reference Period in Western US Dry Conifer Forests.” Forest Ecology and Management 544(September): 121232. 10.1016/j.foreco.2023.121232.

[eap70121-bib-0081] Parks, S. A. , S. Z. Dobrowski , J. D. Shaw , and C. Miller . 2019. “Living on the Edge: Trailing Edge Forests at Risk of Fire‐Facilitated Conversion to Non‐Forest.” Ecosphere 10(3): e02651. 10.1002/ecs2.2651.

[eap70121-bib-0084] Perret, D. L. , D. M. Bell , and H. S. J. Zald . 2025. “Reducing Fire Severity and Extent Bolsters Subalpine Forest Resilience to Global Change Through Key Demographic Pathways.” Global Change Biology 31(2): e70052. 10.1111/gcb.70052. 39907028

[eap70121-bib-0085] Peterson, D. W. , and D. L. Peterson . 1994. “Effects of Climate on Radial Growth of Subalpine Conifers in the North Cascade Mountains.” Canadian Journal of Forest Research 24(9): 1921–1932. 10.1139/x94-247.

[eap70121-bib-0122] R Core Team . 2023. R: A Language and Environment for Statistical Computing. Vienna, Austria: R Foundation for Statistical Computing https://www.R-project.org.

[eap70121-bib-0086] Radeloff, V. C. , D. P. Helmers , H. A. Kramer , M. H. Mockrin , P. M. Alexandre , A. Bar‐Massada , V. Butsic , et al. 2018. “Rapid Growth of the US Wildland‐Urban Interface Raises Wildfire Risk.” Proceedings of the National Academy of Sciences of the United States of America 115(13): 3314–3319. 10.1073/pnas.1718850115. 29531054 PMC5879688

[eap70121-bib-0087] Rammer, W. , D. Thom , M. Baumann , K. Braziunas , C. Dollinger , J. Kerber , J. Mohr , and R. Seidl . 2024. “The Individual‐Based Forest Landscape and Disturbance Model iLand: Overview, Progress, and Outlook.” Ecological Modelling 495(September): 110785. 10.1016/j.ecolmodel.2024.110785.

[eap70121-bib-0088] Rodman, K. C. , K. T. Davis , S. A. Parks , T. B. Chapman , J. D. Coop , J. M. Iniguez , J. P. Roccaforte , et al. 2023. “Refuge‐Yeah or Refuge‐Nah? Predicting Locations of Forest Resistance and Recruitment in a Fiery World.” Global Change Biology 29(24): 7029–7050. 10.1111/gcb.16939. 37706328

[eap70121-bib-0089] Romme, W. H. , and D. G. Despain . 1989. “Historical Perspective on the Yellowstone Fires of 1988.” Bioscience 39(10): 695–699.

[eap70121-bib-0091] Schuurman, G. W. , D. N. Cole , A. E. Cravens , S. Covington , S. D. Crausbay , C. H. Hoffman , D. J. Lawrence , et al. 2022. “Navigating Ecological Transformation: Resist–Accept–Direct as a Path to a New Resource Management Paradigm.” Bioscience 72(1): 16–29. 10.1093/biosci/biab067.

[eap70121-bib-0090] Schuurman, G. W. , H.‐H. Cat , D. Cole , D. Lawrence , J. Morton , D. Magness , A. Cravens , S. Covington , R. O'Malley , and N. Fisichelli . 2020. “Resist‐Accept‐Direct (RAD)—A Framework for the 21st‐Century Natural Resource Manager.” National Park Service. 10.36967/nrr-2283597.

[eap70121-bib-0092] Sciaini, M. , M. Fritsch , C. Scherer , and C. E. Simpkins . 2018. “NLMR and Landscapetools: An Integrated Environment for Simulating and Modifying Neutral Landscape Models in R.” Methods in Ecology and Evolution 9(11): 2240–2248. 10.1111/2041-210X.13076.

[eap70121-bib-0095] Seidl, R. , and M. G. Turner . 2022. “Post‐Disturbance Reorganization of Forest Ecosystems in a Changing World.” Proceedings of the National Academy of Sciences of the United States of America 119(28): e2202190119. 10.1073/pnas.2202190119. 35787053 PMC9282434

[eap70121-bib-0094] Seidl, R. , W. Rammer , and T. A. Spies . 2014. “Disturbance Legacies Increase the Resilience of Forest Ecosystem Structure, Composition, and Functioning.” Ecological Applications 24(8): 2063–2077. 10.1890/14-0255.1. 27053913 PMC4820056

[eap70121-bib-0093] Seidl, R. , W. Rammer , R. M. Scheller , and T. A. Spies . 2012. “An Individual‐Based Process Model to Simulate Landscape‐Scale Forest Ecosystem Dynamics.” Ecological Modelling 231: 87–100.

[eap70121-bib-0096] Smithwick, E. a. H. , M. G. Ryan , D. M. Kashian , W. H. Romme , D. B. Tinker , and M. G. Turner . 2009. “Modeling the Effects of Fire and Climate Change on Carbon and Nitrogen Storage in Lodgepole Pine (Pinus Contorta) Stands.” Global Change Biology 15(3): 535–548. 10.1111/j.1365-2486.2008.01659.x.

[eap70121-bib-0097] Sommers, M. , and M. D. Flannigan . 2022. “Green Islands in a Sea of Fire: The Role of Fire Refugia in the Forests of Alberta.” Environmental Reviews 30(3): 402–417. 10.1139/er-2021-0115.

[eap70121-bib-0098] Steenvoorden, J. , A. J. H. Meddens , A. J. Martinez , L. J. Foster , and W. D. Kissling . 2019. “The Potential Importance of Unburned Islands as Refugia for the Persistence of Wildlife Species in Fire‐Prone Ecosystems.” Ecology and Evolution 9(15): 8800–8812. 10.1002/ece3.5432. 31410281 PMC6686341

[eap70121-bib-0099] Stephens, S. L. , J. K. Agee , P. Z. Fulé , M. P. North , W. H. Romme , T. W. Swetnam , and M. G. Turner . 2013. “Managing Forests and Fire in Changing Climates.” Science 342(6154): 41–42. 10.1126/science.1240294. 24092714

[eap70121-bib-0100] Stevens‐Rumann, C. S. , and P. Morgan . 2019. “Tree Regeneration Following Wildfires in the Western US: A Review.” Fire Ecology 15(1): 15. 10.1186/s42408-019-0032-1.

[eap70121-bib-0101] Stevens‐Rumann, C. S. , S. J. Prichard , E. Whitman , M.‐A. Parisien , and A. J. H. Meddens . 2022. “Considering Regeneration Failure in the Context of Changing Climate and Disturbance Regimes in Western North America.” Canadian Journal of Forest Research 52(10): 1281–1302. 10.1139/cjfr-2022-0054.

[eap70121-bib-0102] Stueve, K. M. , D. L. Cerney , R. M. Rochefort , and L. L. Kurth . 2009. “Post‐Fire Tree Establishment Patterns at the Alpine Treeline Ecotone: Mount Rainier National Park, Washington, USA.” Journal of Vegetation Science 20(1): 107–120. 10.1111/j.1654-1103.2009.05437.x.

[eap70121-bib-0103] Tanner, S. , F. Lupi , and C. Garnache . 2022. “Estimating Visitor Preferences for Recreation Sites in Wildfire Prone Areas.” International Journal of Wildland Fire 31(9): 871–885. 10.1071/WF21133.

[eap70121-bib-0104] Thom, D. , W. Rammer , K. Albrich , K. H. Braziunas , L. Dobor , C. Dollinger , W. D. Hansen , et al. 2024. “Parameters of 150 Temperate and Boreal Tree Species and Provenances for an Individual‐Based Forest Landscape and Disturbance Model.” Data in Brief 55(August): 110662. 10.1016/j.dib.2024.110662. 39234067 PMC11372383

[eap70121-bib-0105] Tinker, D. B. , W. H. Romme , W. W. Hargrove , R. H. Gardner , and M. G. Turner . 1994. “Landscape‐Scale Heterogeneity in Lodgepole Pine Serotiny.” Canadian Journal of Forest Research 24(5): 897–903. 10.1139/x94-118.

[eap70121-bib-0106] Tucker, M. M. , and D. M. Kashian . 2018. “Pre‐Fire Forest Remnants Affect Post‐Fire Plant Community Structure and Composition.” Forest Ecology and Management 408(January): 103–111. 10.1016/j.foreco.2017.10.038.

[eap70121-bib-0112] Turner, M. G. , D. B. Tinker , W. H. Romme , D. M. Kashian , and C. M. Litton . 2004. “Landscape Patterns of Sapling Density, Leaf Area, and Aboveground Net Primary Production in Postfire Lodgepole Pine Forests, Yellowstone National Park (USA).” Ecosystems 7(7): 751–775. 10.1007/s10021-004-0011-4.

[eap70121-bib-0107] Turner, M. G. , K. H. Braziunas , W. D. Hansen , T. J. Hoecker , W. Rammer , Z. Ratajczak , A. L. Westerling , and R. Seidl . 2022. “The Magnitude, Direction, and Tempo of Forest Change in Greater Yellowstone in a Warmer World with More Fire.” Ecological Monographs 92(1): e01485. 10.1002/ecm.1485.

[eap70121-bib-0109] Turner, M. G. , R. V. O'Neill , R. H. Gardner , and B. T. Milne . 1989. “Effects of Changing Spatial Scale on the Analysis of Landscape Pattern.” Landscape Ecology 3(3): 153–162. 10.1007/BF00131534.

[eap70121-bib-0110] Turner, M. G. , W. H. Romme , and R. H. Gardner . 1999. “Prefire Heterogeneity, Fire Severity, and Early Postfire Plant Reestablishment in Subalpine Forests of Yellowstone National Park, Wyoming.” International Journal of Wildland Fire 9(1): 21. 10.1071/WF99003.

[eap70121-bib-0111] Turner, M. G. , W. H. Romme , R. H. Gardner , and W. W. Hargrove . 1997. “Effects of Fire Size and Pattern on Early Succession in Yellowstone National Park.” Ecological Monographs 67(4): 411–433. 10.1890/0012-9615(1997)067[0411:EOFSAP]2.0.CO;2.

[eap70121-bib-0108] Turner, M. G. , W. W. Hargrove , R. H. Gardner , and W. H. Romme . 1994. “Effects of Fire on Landscape Heterogeneity in Yellowstone National Park, Wyoming.” Journal of Vegetation Science 5(5): 731–742. 10.2307/3235886.

[eap70121-bib-0113] US Department of Interior and US Department of Agriculture . 2014. National Cohesive Wildland Fire Management Strategy. Washington, DC: The U.S. Departments of the Interior and Agriculture. https://www.forestsandrangelands.gov/strategy.

[eap70121-bib-0115] White, J. W. , A. Rassweiler , J. F. Samhouri , A. C. Stier , and C. White . 2014. “Ecologists Should Not Use Statistical Significance Tests to Interpret Simulation Model Results.” Oikos 123(4): 385–388. 10.1111/j.1600-0706.2013.01073.x.

[eap70121-bib-0116] Whitlock, C. 1993. “Postglacial Vegetation and Climate of Grand Teton and Southern Yellowstone National Parks.” Ecological Monographs 63(2): 173–198. 10.2307/2937179.

[eap70121-bib-0117] Whitlock, C. , W. E. Dean , S. C. Fritz , L. R. Stevens , J. R. Stone , M. J. Power , J. R. Rosenbaum , K. L. Pierce , and B. B. Bracht‐Flyr . 2012. “Holocene Seasonal Variability Inferred from Multiple Proxy Records from Crevice Lake, Yellowstone National Park, USA.” Palaeogeography, Palaeoclimatology, Palaeoecology 331–332(May): 90–103. 10.1016/j.palaeo.2012.03.001.

[eap70121-bib-0118] Wickham, H. , M. Averick , J. Bryan , W. Chang , L. McGowan , R. François , G. Grolemund , et al. 2019. “Welcome to the Tidyverse.” Journal of Open Source Software 4(43): 1686. 10.21105/joss.01686.

[eap70121-bib-0120] With, K. A. , and A. W. King . 1997. “The Use and Misuse of Neutral Landscape Models in Ecology.” Oikos 79(2): 219–229. 10.2307/3546007.

[eap70121-bib-0119] With, K. A. , R. H. Gardner , and M. G. Turner . 1997. “Landscape Connectivity and Population Distributions in Heterogeneous Environments.” Oikos 78(1): 151. 10.2307/3545811.

