# Supplementary material for: Can fire exclusion zones enhance postfire tree regeneration? A simulation study in subalpine conifer forests
Source: Ecol Appl. 2025 Oct 16;35(7):e70121. doi: 10.1002/eap.70121 (PMC12529467; doi:10.1002/eap.70121)
Supplement: Supplementary file 1 — Appendix S1. [file EAP-35-e70121-s001.pdf]

Can fire exclusion zones enhance postfire tree regeneration?

A simulation study in subalpine conifer forests

Timon T. Keller, Diane C. Abendroth, Kristin H. Braziunas, Christina Dollinger,

Paul R. Hood, Garrett J. Knowlton, Rupert Seidl, and Monica G. Turner

Journal: Ecological Applications

Appendix S1: Supplemental analyses

Table S1: Summary of two-way ANOVAs fit to log_{10} transformed seedling density five years postfire early (2026 – 2050) and late (2076 – 2100) in the century to assess whether variation in spatial patterns of fire exclusion zones (NLM) or variation in fire history (Fire) has a greater effect on postfire tree regeneration. All models follow the formula log_{10}(mean seedlings ha^{-1} $iteration^{-1}$ + 1) ~ NLM × Fire. We used F-Values to assess predictor importance, rather than relying on p-values. We only used 30% fire exclusion zones in hot-dry climate for this supplemental analysis. Species codes are as follows: Abla = subalpine fir, Pien = Engelmann spruce, Psme = Douglas-fir, PicS = serotinous lodgepole pine, Pico = non-serotinous lodgepole pine.

**Model**	**Species**	**Fire exclusion zone**	**Period**	**Term**	**DF**	**F-Value**	***p***
1	Abla	30% clumped	2026-2050	NLM	9	1.53	1.52E-01
				Fire	9	2.86	5.53E-03
				Residuals	81	NA	NA
2	Abla	30% dispersed	2026-2050	NLM	9	0.67	7.37E-01
				Fire	9	1.54	1.49E-01
				Residuals	81	NA	NA
3	Abla	30% clumped	2076-2100	NLM	9	6.70	4.12E-07
				Fire	9	9.78	5.62E-10
				Residuals	81	NA	NA
4	Abla	30% dispersed	2076-2100	NLM	9	0.66	7.44E-01
				Fire	9	8.65	5.59E-09
				Residuals	81	NA	NA
5	Pien	30% clumped	2026-2050	NLM	9	1.89	6.45E-02
				Fire	9	4.47	8.99E-05
				Residuals	81	NA	NA
6	Pien	30% dispersed	2026-2050	NLM	9	0.93	5.08E-01
				Fire	9	3.53	9.81E-04
				Residuals	81	NA	NA
7	Pien	30% clumped	2076-2100	NLM	9	5.41	8.65E-06
				Fire	9	6.22	1.25E-06
				Residuals	81	NA	NA
8	Pien	30% dispersed	2076-2100	NLM	9	0.38	9.40E-01
				Fire	9	8.10	1.80E-08
				Residuals	81	NA	NA
9	Psme	30% clumped	2026-2050	NLM	9	1.39	2.08E-01
				Fire	9	4.12	2.14E-04
				Residuals	81	NA	NA
10	Psme	30% dispersed	2026-2050	NLM	9	1.29	2.54E-01
				Fire	9	4.07	2.47E-04
				Residuals	81	NA	NA
11	Psme	30% clumped	2076-2100	NLM	9	1.34	2.32E-01
				Fire	9	3.77	5.26E-04
				Residuals	81	NA	NA

Model	Species	Fire exclusion zones	Period	Term	DF	F-Value	*p*
12	Psme	30% dispersed	2076-2100	NLM	9	1.53	1.52E-01
				Fire	9	5.25	1.26E-05
				Residuals	81	NA	NA
13	Pics	30% clumped	2026-2050	NLM	9	1.48	1.69E-01
				Fire	9	2.33	2.15E-02
				Residuals	81	NA	NA
14	Pics	30% dispersed	2026-2050	NLM	9	0.79	6.22E-01
				Fire	9	4.19	1.79E-04
				Residuals	81	NA	NA
15	Pics	30% clumped	2076-2100	NLM	9	2.25	2.68E-02
				Fire	9	6.78	3.43E-07
				Residuals	81	NA	NA
16	Pics	30% dispersed	2076-2100	NLM	9	1.53	1.52E-01
				Fire	9	9.51	9.69E-10
				Residuals	81	NA	NA
17	Pico	30% clumped	2026-2050	NLM	9	1.51	1.57E-01
				Fire	9	1.93	5.90E-02
				Residuals	81	NA	NA
18	Pico	30% dispersed	2026-2050	NLM	9	1.01	4.39E-01
				Fire	9	1.49	1.65E-01
				Residuals	81	NA	NA
19	Pico	30% clumped	2076-2100	NLM	9	6.84	2.99E-07
				Fire	9	3.96	3.25E-04
				Residuals	81	NA	NA
20	Pico	30% dispersed	2076-2100	NLM	9	1.33	2.34E-01
				Fire	9	4.18	1.84E-04
				Residuals	81	NA	NA

Residuals vs Fitted

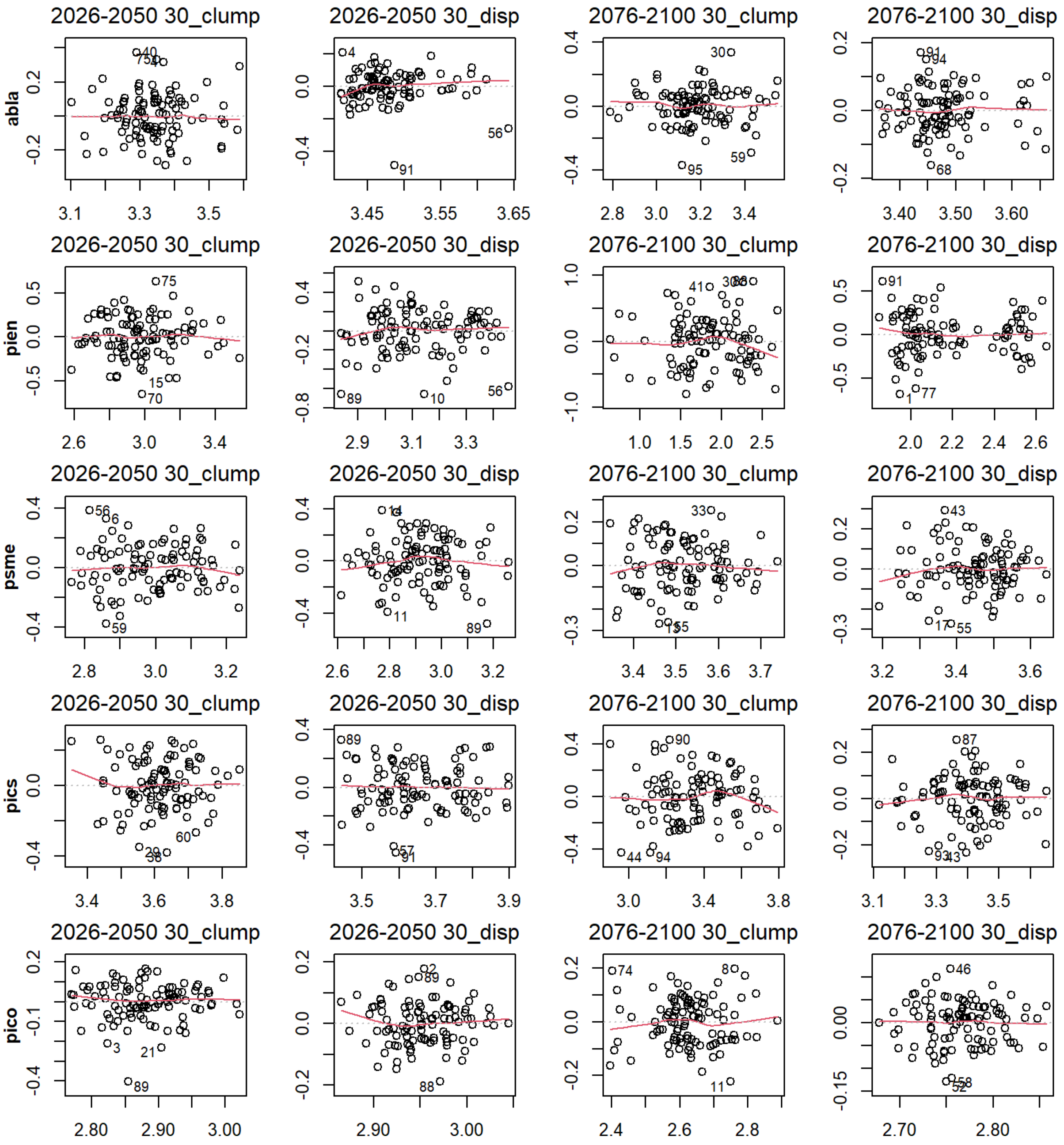

Figure S1: Residuals vs. fitted values diagnostic plots of two-way ANOVAs fit to log_{10} transformed seedling density five years postfire early (2026 – 2050) and late (2076 – 2100) in the century. We only used 30% fire exclusion zones in hot-dry climate for this supplemental analysis. Here, “30_clump” refers to 30% clumped configurations and “30_disp” refers to 30% dispersed configurations. Species codes are as follows: Abla = subalpine fir, Pien = Engelmann spruce, Psme = Douglas-fir, PicS = serotinous lodgepole pine, Pico = non-serotinous lodgepole pine.

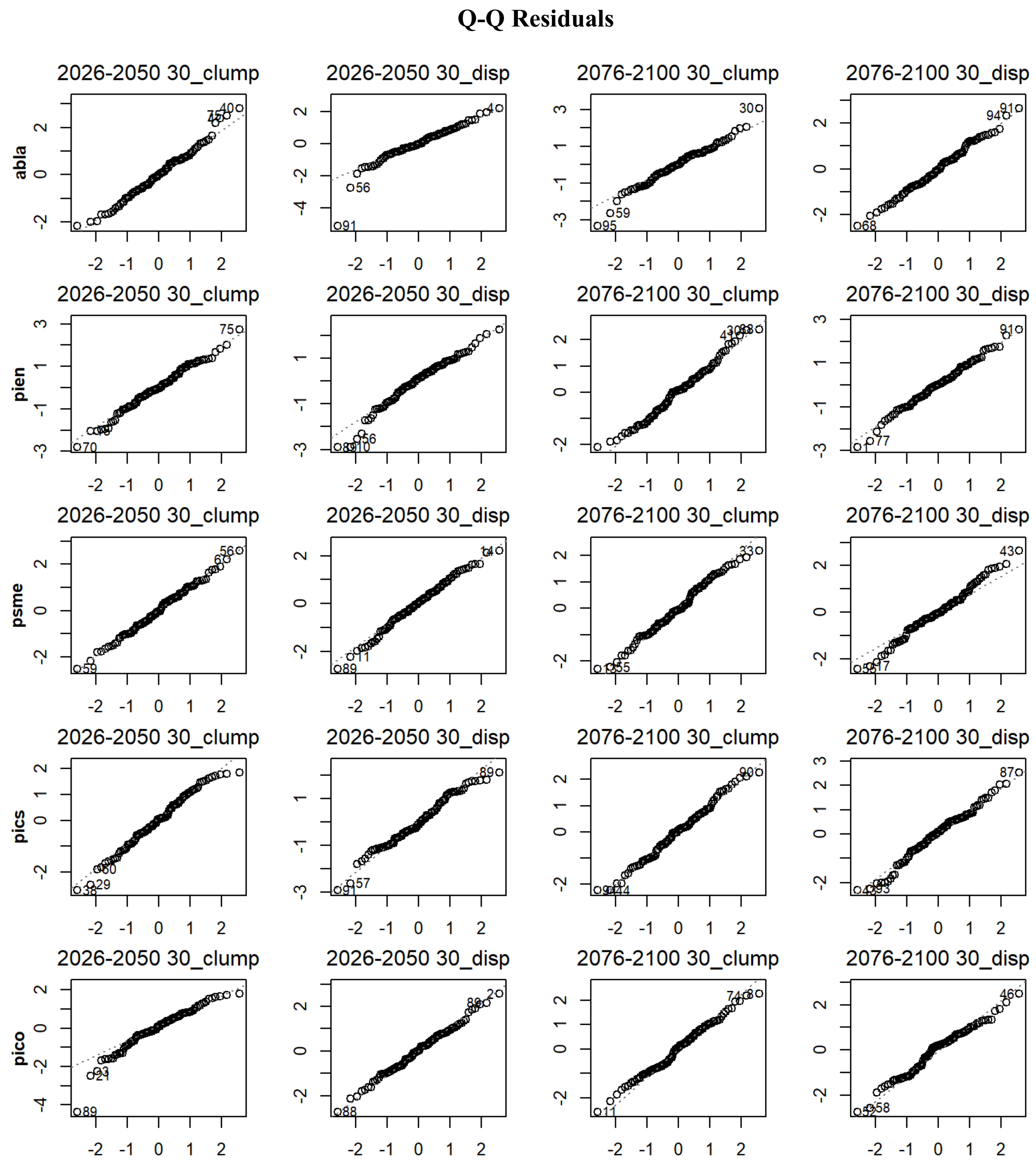

Figure S2: Q-Q Residuals diagnostic plots of two-way ANOVAs fit to $\log_{10}$ transformed seedling density five years postfire early (2026 – 2050) and late (2076 – 2100) in the century. We only used 30% fire exclusion zones in hot-dry climate for this supplemental analysis. Here, “30_clump” refers to 30% clumped configurations and “30_disp” refers to 30% dispersed configurations. Species codes are as follows: Abla = subalpine fir, Pien = Engelmann spruce, Psme = Douglas-fir, PicS = serotinous lodgepole pine, Pico = non-serotinous lodgepole pine.

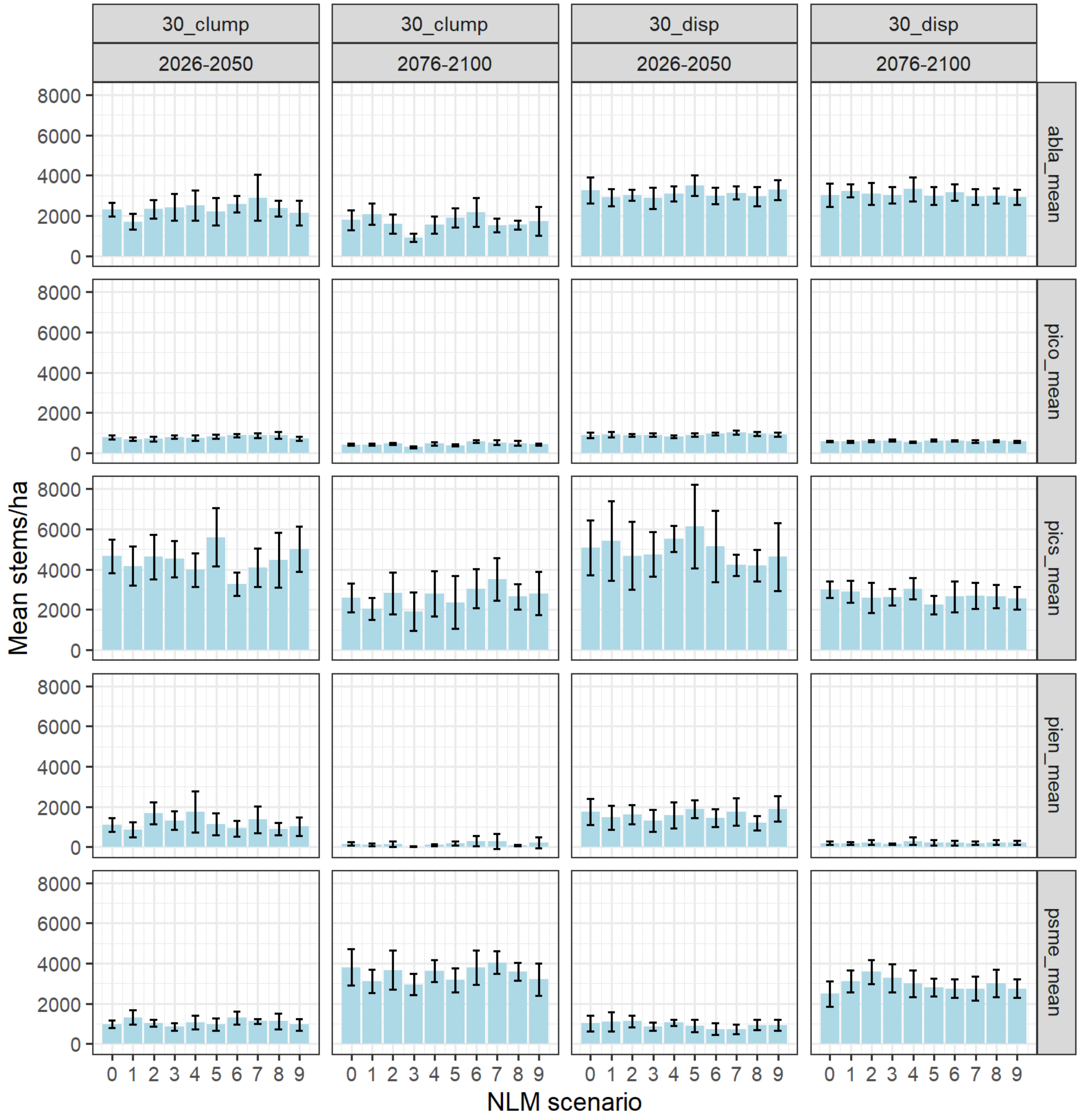

Figure S3: Mean (± 2 SE) stems ha^{-1} within fire perimeters in simulations with unique NLM scenarios of 30% clumped and 30% dispersed fire exclusion zones simulated in hot-dry climate (HadGEM2 ES365). Each NLM scenario was replicated with ten unique fire histories (10 NLMs x 10 fire histories x 2 configurations = 200 simulations). Here, "30_clump" refers to 30% clumped configurations and "30_disp" refers to 30% dispersed configurations. Species codes are as follows: Abla = subalpine fir, Pien = Engelmann spruce, Psme = Douglas-fir, PicS = serotinous lodgepole pine, Pico = non-serotinous lodgepole pine.

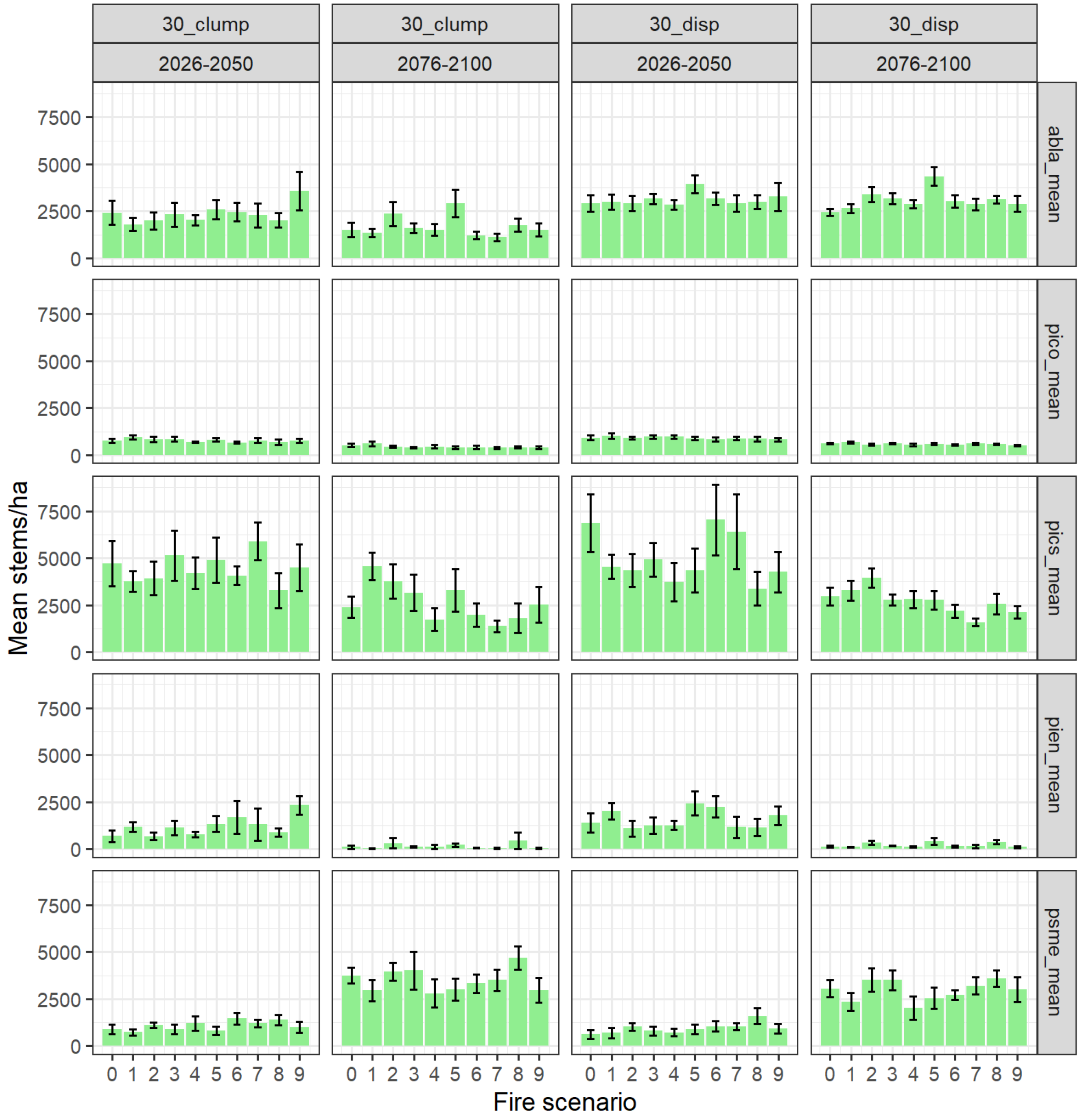

Figure S4: Mean (± 2 SE) stems ha^{-1} within fire perimeters in simulations with unique fire scenarios simulated in hot-dry climate (HadGEM2 ES365). Each fire scenario was replicated with ten unique NLMs of 30% clumped and 30% dispersed fire exclusion zones (10 fire histories x 10 NLMs x 2 configurations = 200 simulations). Here, “30_clump” refers to 30% clumped configurations and “30_disp” refers to 30% dispersed configurations. Species codes are as follows: Abla = subalpine fir, Pien = Engelmann spruce, Psme = Douglas-fir, PicS = serotinous lodgepole pine, Pico = non-serotinous lodgepole pine.

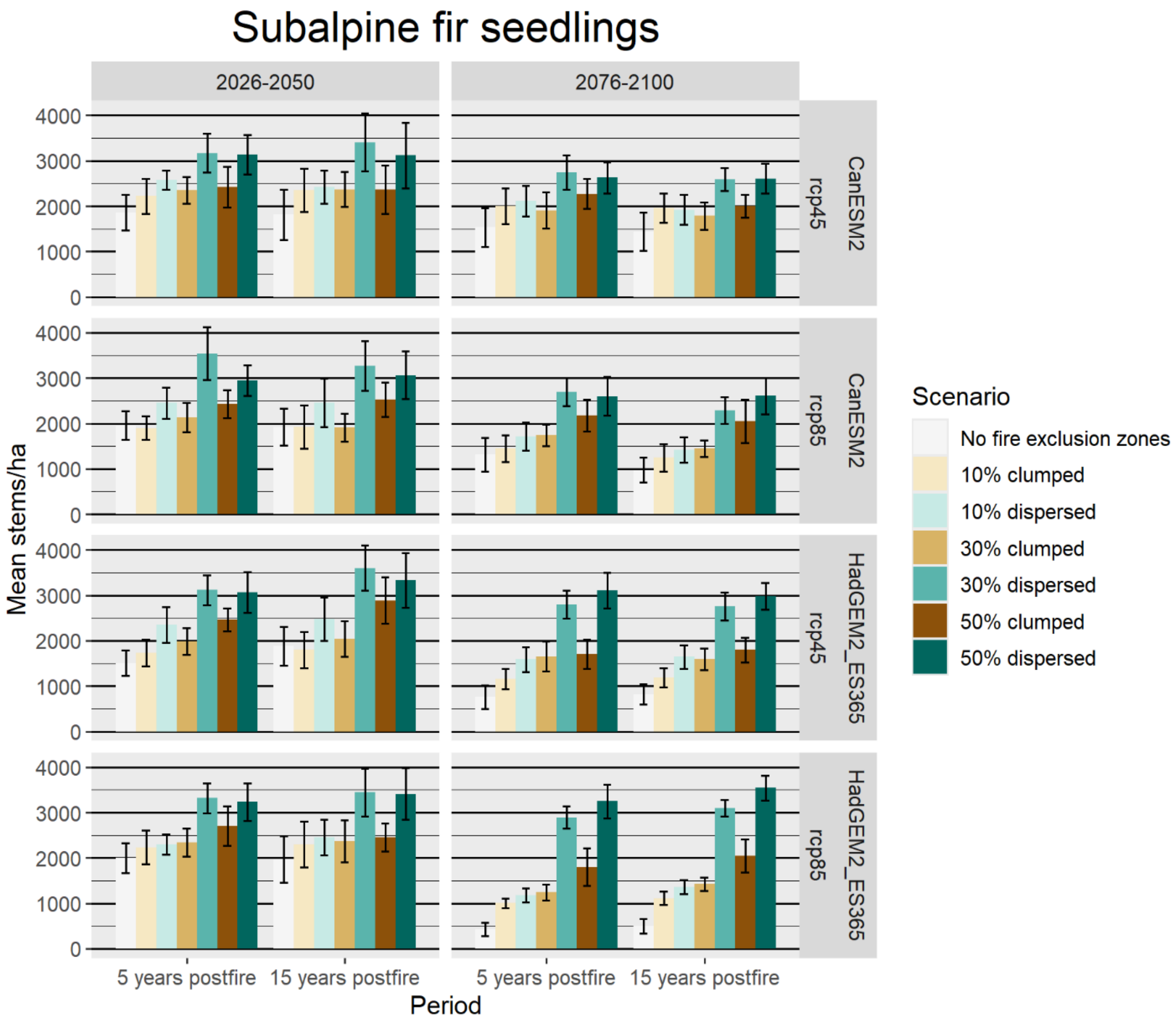

Figure S5: Mean (± 2 SE) postfire tree regeneration of subalpine fir (abla) at different time windows postfire (5 vs 15 years) early and late in the century for all climate scenarios. Trends in regeneration are consistent in both time windows, and differences are minimal with some infilling at 15-years postfire.

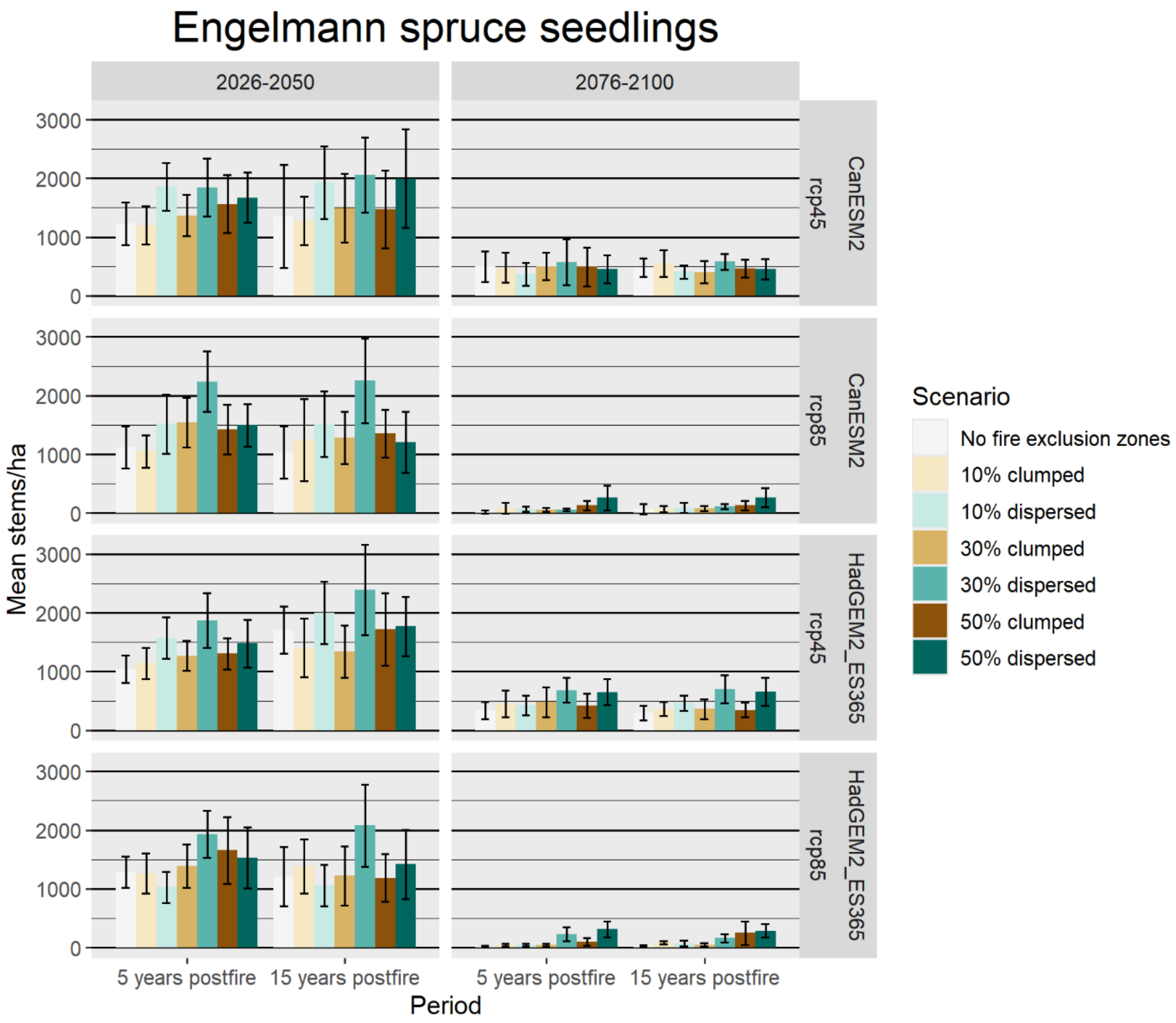

Figure S6: Mean (± 2 SE) postfire tree regeneration of Engelmann spruce at different time windows postfire (5 vs 15 years) early and late in the century for all climate scenarios. Trends in regeneration are consistent in both time windows, and differences are minimal with some infilling and strengthening effects of fire exclusion zones at 15-years postfire.

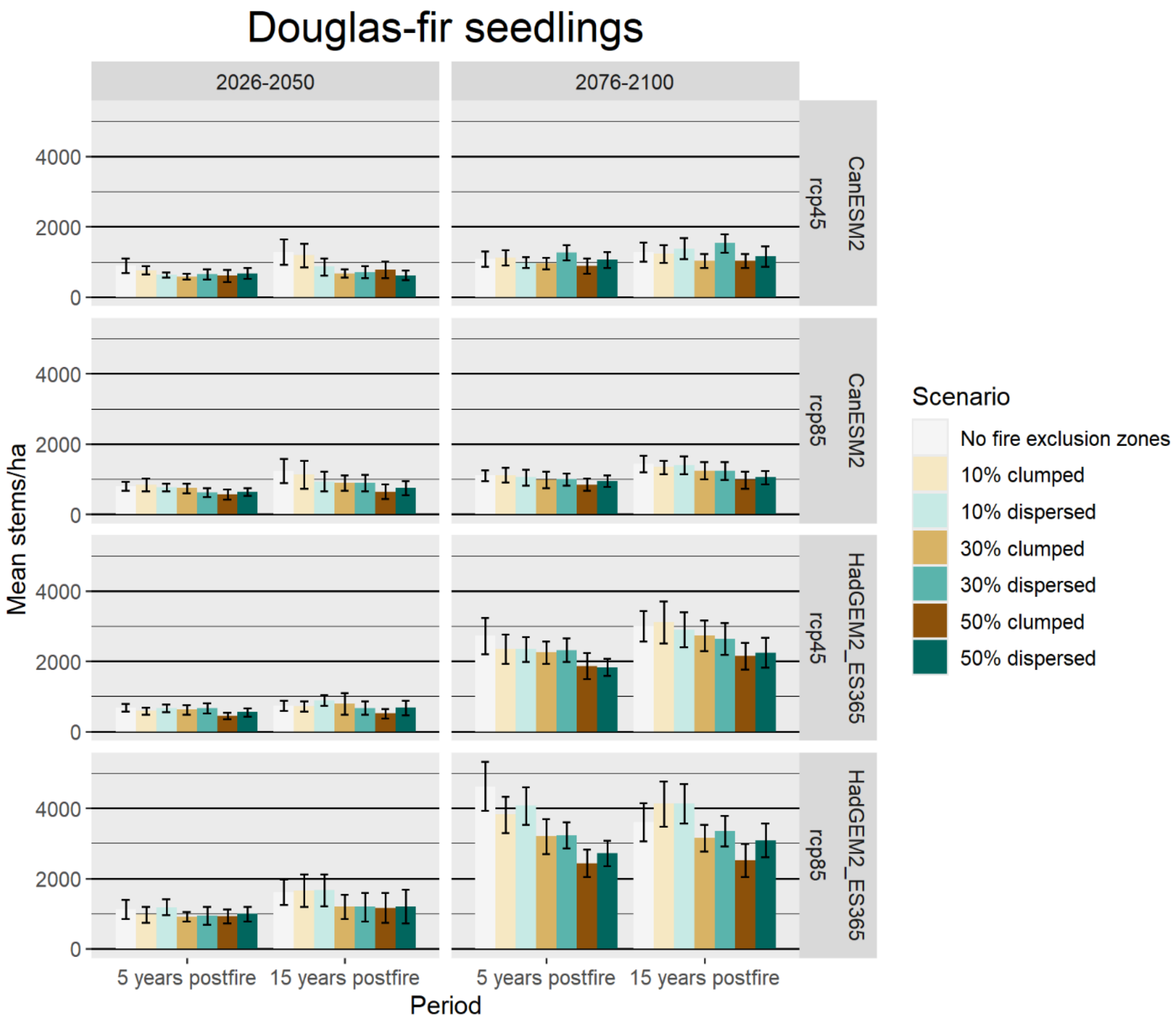

Figure S7: Mean (± 2 SE) postfire tree regeneration of Douglas-fir at different time windows postfire (5 vs 15 years) early and late in the century for all climate scenarios. Trends in regeneration are consistent in both time windows, and differences are minimal with some infilling at 15-years postfire.

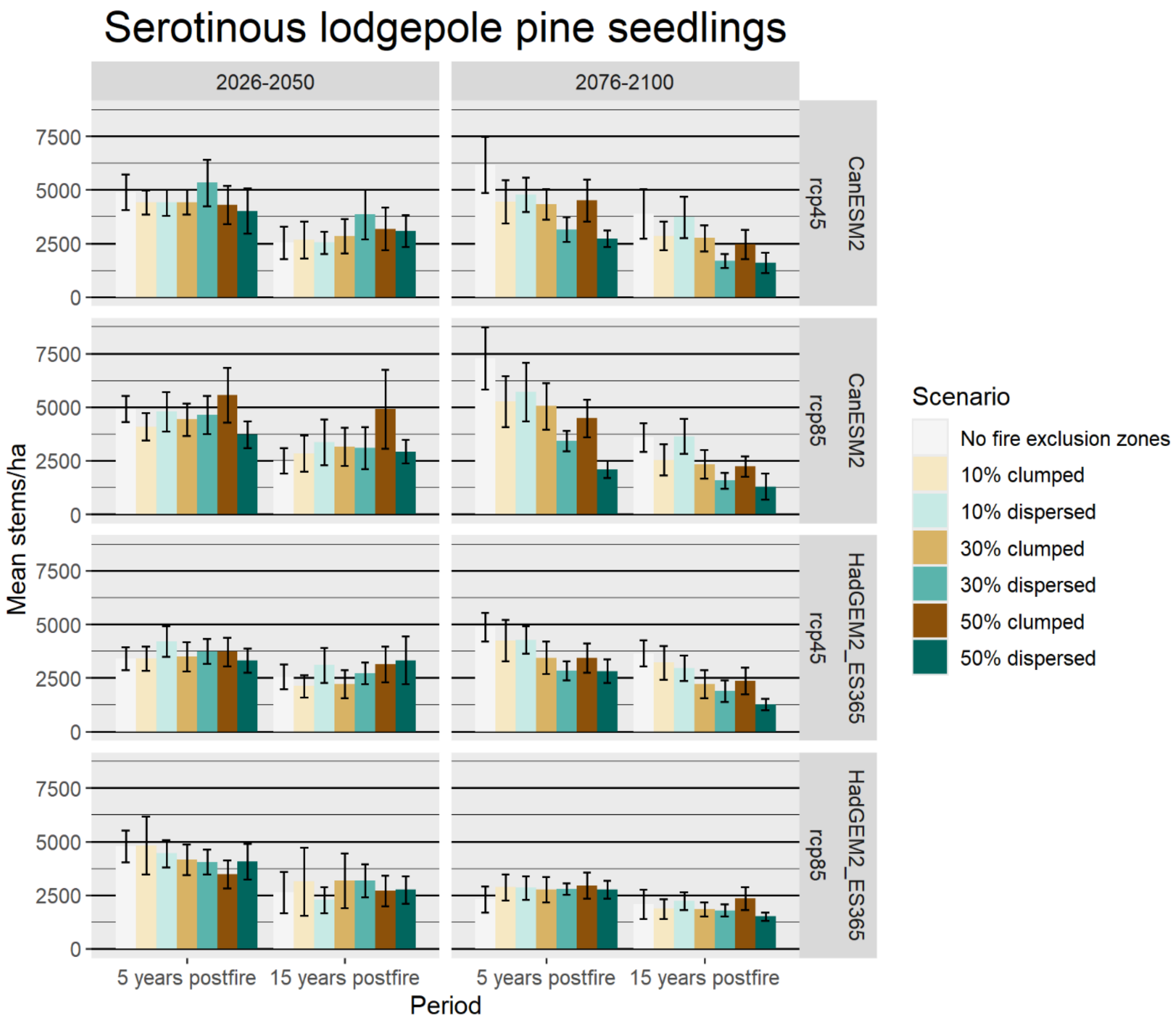

Figure S8: Mean (± 2 SE) postfire tree regeneration of serotinous lodgepole pine at different time windows postfire (5 vs 15 years) early and late in the century for all climate scenarios. Trends in regeneration are consistent in both time windows, and differences are minimal with some thinning at 15-years postfire which is consistent with patterns of postfire lodgepole pine regeneration.

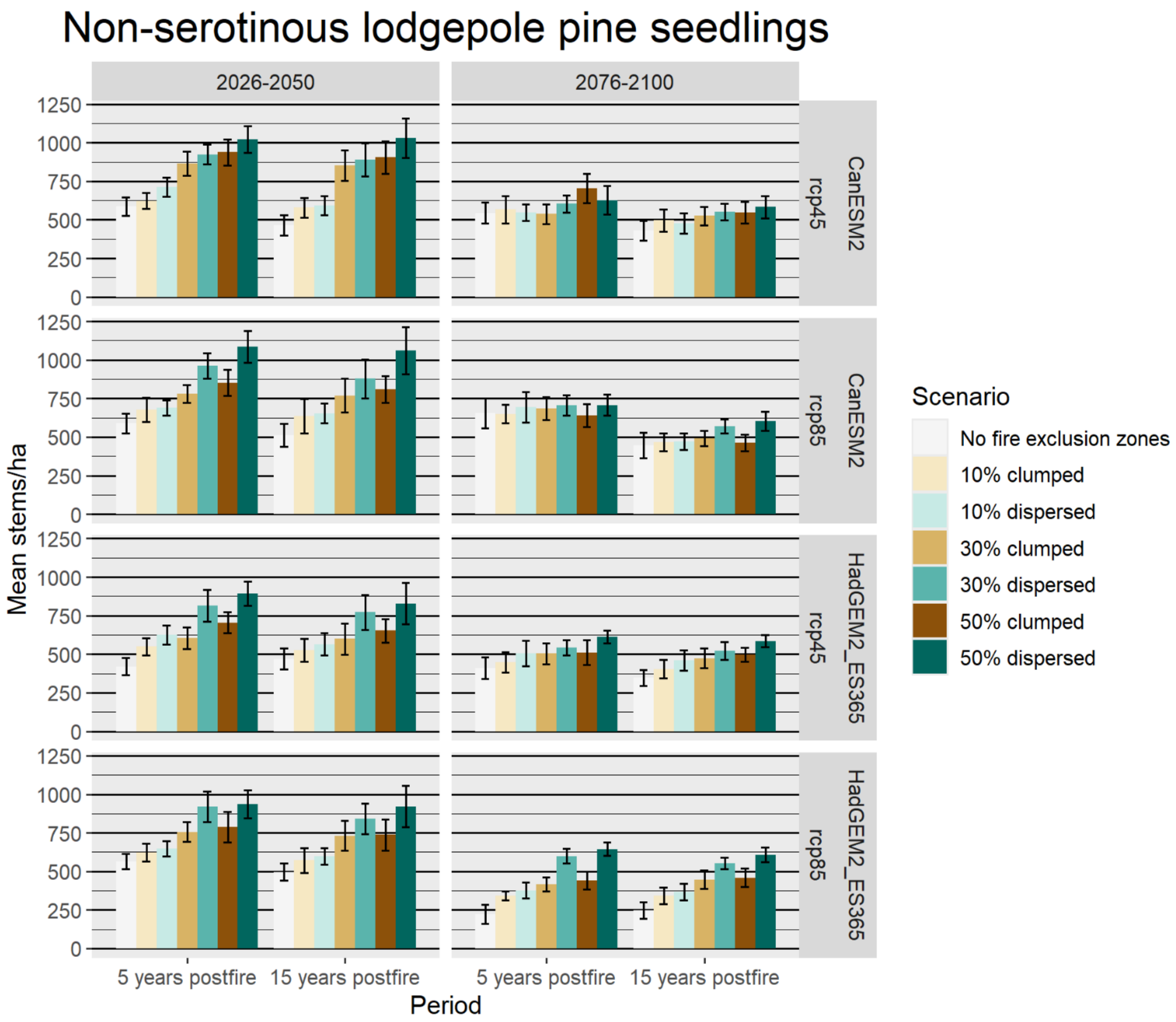

Figure S9: Mean (± 2 SE) postfire tree regeneration of non-serotinous lodgepole pine at different time windows postfire (5 vs 15 years) early and late in the century for all climate scenarios. Trends in regeneration are consistent in both time windows, and differences are minimal with some thinning at 15-years postfire which is consistent with patterns of postfire lodgepole pine regeneration.
