# Supplementary material for: Can fire exclusion zones enhance postfire tree regeneration? A simulation study in subalpine conifer forests
Source: Ecol Appl. 2025 Oct 16;35(7):e70121. doi: 10.1002/eap.70121 (PMC12529467; doi:10.1002/eap.70121)
Supplement: Supplementary file 2 — Appendix S2. [file EAP-35-e70121-s002.pdf]

Can fire exclusion zones enhance postfire tree regeneration?

A simulation study in subalpine conifer forests

Timon T. Keller, Diane C. Abendroth, Kristin H. Braziunas, Christina Dollinger,

Paul R. Hood, Garrett J. Knowlton, Rupert Seidl, and Monica G. Turner

Journal: Ecological Applications

Appendix S2: Supplemental tables and figures

Table S1: Summary of two-way ANOVAs fit to $\log_{10}$(seedling density + 1) five years postfire early (2026 – 2050) and late (2076 – 2100) in the century. All models follow the formula $\log_{10}$(mean seedlings ha^{-1} $iteration^{-1}$ + 1) ~ amount × configuration (of fire exclusion zones). We used F-Values to assess predictor importance, rather than relying on p-values. Scenarios without fire exclusion zones were excluded from this analysis. Species codes are as follows: Abla = subalpine fir, Pien = Engelmann spruce, Psme = Douglas-fir, PicS = serotinous lodgepole pine, Pico = non-serotinous lodgepole pine.

Model	Species	Period	Term	DF	F-Value	*p*
1	Abla	Early	Amount	2	25.67	2.6E-11
			Config.	1	89.65	1.3E-19
			Amount:Config.	2	4.75	9.0E-03
			Residuals	474		
2	Abla	Late	Amount	2	74.97	5.1E-29
			Config.	1	130.18	8.3E-27
			Amount:Config.	2	10.51	3.4E-05
			Residuals	474		
3	Pien	Early	Amount	2	5.35	5.0E-03
			Config.	1	8.56	3.6E-03
			Amount:Config.	2	3.35	3.6E-02
			Residuals	474		
4	Pien	Late	Amount	2	6.74	1.3E-03
			Config.	1	14.93	1.3E-04
			Amount:Config.	2	2.00	1.4E-01
			Residuals	474		
5	Psme	Early	Amount	2	8.13	3.4E-04
			Config.	1	1.35	2.5E-01
			Amount:Config.	2	1.76	1.7E-01
			Residuals	474		
6	Psme	Late	Amount	2	6.39	1.8E-03
			Config.	1	1.30	2.5E-01
			Amount:Config.	2	0.64	5.3E-01
			Residuals	474		
7	PicS	Early	Amount	2	2.64	7.2E-02
			Config.	1	0.05	8.2E-01
			Amount:Config.	2	2.25	1.1E-01
			Residuals	474		
8	PicS	Late	Amount	2	12.92	3.5E-06
			Config.	1	11.13	9.1E-04
			Amount:Config.	2	6.13	2.3E-03
			Residuals	474		
9	Pico	Early	Amount	2	81.84	3.0E-31
			Config.	1	50.29	4.9E-12
			Amount:Config.	2	2.33	9.9E-02
			Residuals	474		

10	Pico	Late	Amount	2	16.08	1.8E-07
			Config.	1	18.73	1.8E-05
			Amount:Config.	2	1.44	2.4E-01
			Residuals	474		

Table S2. Summary of linear regressions fit to $\log_{10}$(seedling density + 1) five years postfire early (2026 – 2050) in the century with fire exclusion zone scenario as predictor. We used t-values and adjusted R^2 to assess predictor importance, rather than relying on p-values. The effect of each fire exclusion zone scenario is relative to a reference scenario without fire exclusion zones. Species codes are as follows: Abla = subalpine fir, Pien = Engelmann spruce, Psme = Douglas-fir, PicS = serotinous lodgepole pine, Pico = nonserotinous lodgepole pine.

Model	Species	GCM	RCP	Period	Adj. R^2	Overall *p*	Predictor	Estimate	SE	*t*	*p*
1	Abla	HadGEM2-ES365	4.5	Early	0.33	3.2E-11	Intercept	3.1E+00	3.7E-02	8.5E+01	1.4E-117
							10% disp.	2.1E-01	5.2E-02	4.1E+00	7.6E-05
							10% clump.	7.1E-02	5.2E-02	1.4E+00	1.8E-01
							30% disp.	3.5E-01	5.2E-02	6.7E+00	4.7E-10
							30% clump.	1.4E-01	5.2E-02	2.7E+00	8.4E-03
							50% disp.	3.3E-01	5.2E-02	6.4E+00	2.7E-09
							50% clump.	2.5E-01	5.2E-02	4.7E+00	5.5E-06
2	Abla	HadGEM2-ES365	8.5	Early	0.22	5.7E-07	Intercept	3.3E+00	3.4E-02	9.7E+01	4.6E-125
							10% disp.	9.4E-02	4.8E-02	2.0E+00	5.0E-02
							10% clump.	6.1E-02	4.8E-02	1.3E+00	2.0E-01
							30% disp.	2.5E-01	4.8E-02	5.3E+00	4.8E-07
							30% clump.	9.2E-02	4.8E-02	1.9E+00	5.6E-02
							50% disp.	2.4E-01	4.8E-02	5.0E+00	1.9E-06
							50% clump.	1.5E-01	4.8E-02	3.2E+00	1.6E-03
3	Abla	CanESM2	4.5	Early	0.21	1.2E-06	Intercept	3.2E+00	3.4E-02	9.5E+01	2.9E-124
							10% disp.	1.8E-01	4.8E-02	3.7E+00	2.8E-04
							10% clump.	8.9E-02	4.8E-02	1.9E+00	6.4E-02
							30% disp.	2.6E-01	4.8E-02	5.4E+00	3.2E-07
							30% clump.	1.3E-01	4.8E-02	2.7E+00	7.1E-03
							50% disp.	2.5E-01	4.8E-02	5.2E+00	7.2E-07
							50% clump.	1.3E-01	4.8E-02	2.7E+00	8.5E-03
4	Abla	CanESM2	8.5	Early	0.25	4.1E-08	Intercept	3.3E+00	3.4E-02	9.7E+01	2.9E-125
							10% disp.	1.1E-01	4.7E-02	2.4E+00	2.0E-02
							10% clump.	2.3E-03	4.7E-02	4.8E-02	9.6E-01
							30% disp.	2.7E-01	4.7E-02	5.6E+00	1.0E-07
							30% clump.	5.1E-02	4.7E-02	1.1E+00	2.8E-01
							50% disp.	2.0E-01	4.7E-02	4.2E+00	5.0E-05
							50% clump.	1.1E-01	4.7E-02	2.4E+00	2.0E-02

Model	Species	GCM	RCP	Period	Adj. R^2	Overall *p*	Predictor	Estimate	SE	*t*	*p*
9	Pien	HadGEM2-ES365	4.5	Early	0.03	1.4E-01	Intercept	3.0E+00	6.3E-02	4.7E+01	6.2E-85
							10% disp.	1.8E-01	8.9E-02	2.1E+00	4.0E-02
							10% clump.	2.3E-02	8.9E-02	2.6E-01	8.0E-01
							30% disp.	2.2E-01	8.9E-02	2.5E+00	1.3E-02
							30% clump.	8.7E-02	8.9E-02	9.8E-01	3.3E-01
							50% disp.	8.8E-02	8.9E-02	9.9E-01	3.2E-01
							50% clump.	9.9E-02	8.9E-02	1.1E+00	2.7E-01
10	Pien	HadGEM2-ES365	8.5	Early	0.05	4.6E-02	Intercept	3.1E+00	6.5E-02	4.7E+01	4.3E-85
							10% disp.	-1.2E-01	9.1E-02	-1.3E+00	1.9E-01
							10% clump.	-4.9E-02	9.1E-02	-5.4E-01	5.9E-01
							30% disp.	1.9E-01	9.1E-02	2.0E+00	4.4E-02
							30% clump.	2.3E-03	9.1E-02	2.5E-02	9.8E-01
							50% disp.	-2.0E-03	9.1E-02	-2.2E-02	9.8E-01
							50% clump.	7.2E-02	9.1E-02	7.9E-01	4.3E-01
11	Pien	CanESM2	4.5	Early	0.01	3.2E-01	Intercept	3.0E+00	8.0E-02	3.8E+01	1.0E-72
							10% disp.	2.1E-01	1.1E-01	1.9E+00	6.0E-02
							10% clump.	4.5E-03	1.1E-01	4.0E-02	9.7E-01
							30% disp.	1.9E-01	1.1E-01	1.7E+00	8.9E-02
							30% clump.	5.2E-02	1.1E-01	4.6E-01	6.4E-01
							50% disp.	4.4E-02	1.1E-01	3.9E-01	7.0E-01
							50% clump.	9.0E-02	1.1E-01	8.0E-01	4.3E-01
12	Pien	CanESM2	8.5	Early	0.08	1.0E-02	Intercept	2.9E+00	7.7E-02	3.8E+01	5.0E-73
							10% disp.	1.5E-01	1.1E-01	1.4E+00	1.8E-01
							10% clump.	1.6E-02	1.1E-01	1.5E-01	8.8E-01
							30% disp.	3.9E-01	1.1E-01	3.6E+00	4.2E-04
							30% clump.	2.0E-01	1.1E-01	1.8E+00	7.2E-02
							50% disp.	1.9E-01	1.1E-01	1.8E+00	8.1E-02
							50% clump.	1.3E-01	1.1E-01	1.2E+00	2.5E-01

Model	Species	GCM	RCP	Period	Adj. R^2	Overall *p*	Predictor	Estimate	SE	*t*	*p*
17	Psme	HadGEM2-ES365	4.5	Early	0.06	2.7E-02	Intercept	2.8E+00	4.4E-02	6.3E+01	3.0E-101
							10% disp.	-2.4E-02	6.3E-02	-3.9E-01	7.0E-01
							10% clump.	-7.1E-02	6.3E-02	-1.1E+00	2.6E-01
							30% disp.	-5.2E-02	6.3E-02	-8.3E-01	4.1E-01
							30% clump.	-6.9E-02	6.3E-02	-1.1E+00	2.7E-01
							50% disp.	-1.1E-01	6.3E-02	-1.8E+00	7.0E-02
							50% clump.	-2.1E-01	6.3E-02	-3.4E+00	9.3E-04
18	Psme	HadGEM2-ES365	8.5	Early	0.00	4.4E-01	Intercept	3.0E+00	4.6E-02	6.6E+01	4.2E-103
							10% disp.	4.1E-02	6.5E-02	6.3E-01	5.3E-01
							10% clump.	-4.9E-02	6.5E-02	-7.6E-01	4.5E-01
							30% disp.	-8.0E-02	6.5E-02	-1.2E+00	2.2E-01
							30% clump.	-6.5E-02	6.5E-02	-1.0E+00	3.2E-01
							50% disp.	-4.1E-02	6.5E-02	-6.4E-01	5.2E-01
							50% clump.	-8.3E-02	6.5E-02	-1.3E+00	2.0E-01
19	Psme	CanESM2	4.5	Early	0.04	7.7E-02	Intercept	2.9E+00	5.1E-02	5.7E+01	3.0E-95
							10% disp.	-1.1E-01	7.2E-02	-1.6E+00	1.1E-01
							10% clump.	-4.1E-02	7.2E-02	-5.6E-01	5.7E-01
							30% disp.	-1.3E-01	7.2E-02	-1.8E+00	7.3E-02
							30% clump.	-1.5E-01	7.2E-02	-2.1E+00	3.7E-02
							50% disp.	-1.4E-01	7.2E-02	-1.9E+00	6.1E-02
							50% clump.	-2.1E-01	7.2E-02	-3.0E+00	3.7E-03
20	Psme	CanESM2	8.5	Early	0.06	3.1E-02	Intercept	2.9E+00	4.9E-02	5.9E+01	4.0E-97
							10% disp.	-1.5E-02	6.9E-02	-2.2E-01	8.2E-01
							10% clump.	1.7E-04	6.9E-02	2.5E-03	1.0E+00
							30% disp.	-1.3E-01	6.9E-02	-1.8E+00	6.7E-02
							30% clump.	-3.9E-02	6.9E-02	-5.7E-01	5.7E-01
							50% disp.	-1.0E-01	6.9E-02	-1.5E+00	1.4E-01
							50% clump.	-2.0E-01	6.9E-02	-2.9E+00	5.0E-03

Model	Species	GCM	RCP	Period	Adj. R^2	Overall *p*	Predictor	Estimate	SE	*t*	*p*
25	PicS	HadGEM2-ES365	4.5	Early	-0.02	6.8E-01	Intercept	3.5E+00	4.4E-02	7.8E+01	2.9E-113
							10% disp.	9.3E-02	6.3E-02	1.5E+00	1.4E-01
							10% clump.	7.3E-03	6.3E-02	1.2E-01	9.1E-01
							30% disp.	5.5E-02	6.3E-02	8.7E-01	3.9E-01
							30% clump.	1.1E-02	6.3E-02	1.8E-01	8.6E-01
							50% disp.	-3.0E-03	6.3E-02	-4.7E-02	9.6E-01
							50% clump.	5.0E-02	6.3E-02	8.0E-01	4.3E-01
26	PicS	HadGEM2-ES365	8.5	Early	0.04	8.6E-02	Intercept	3.7E+00	3.7E-02	9.8E+01	1.3E-125
							10% disp.	-2.7E-02	5.3E-02	-5.1E-01	6.1E-01
							10% clump.	-1.8E-02	5.3E-02	-3.4E-01	7.4E-01
							30% disp.	-7.0E-02	5.3E-02	-1.3E+00	1.9E-01
							30% clump.	-6.7E-02	5.3E-02	-1.3E+00	2.1E-01
							50% disp.	-8.9E-02	5.3E-02	-1.7E+00	9.6E-02
							50% clump.	-1.5E-01	5.3E-02	-2.9E+00	4.7E-03
27	PicS	CanESM2	4.5	Early	0.01	3.4E-01	Intercept	3.7E+00	4.1E-02	9.0E+01	5.7E-121
							10% disp.	-3.3E-02	5.8E-02	-5.8E-01	5.6E-01
							10% clump.	-3.0E-02	5.8E-02	-5.2E-01	6.0E-01
							30% disp.	1.8E-02	5.8E-02	3.1E-01	7.5E-01
							30% clump.	-3.1E-02	5.8E-02	-5.4E-01	5.9E-01
							50% disp.	-1.1E-01	5.8E-02	-2.0E+00	5.1E-02
							50% clump.	-7.1E-02	5.8E-02	-1.2E+00	2.2E-01
28	PicS	CanESM2	8.5	Early	0.03	1.0E-01	Intercept	3.7E+00	3.8E-02	9.5E+01	2.4E-124
							10% disp.	-3.5E-02	5.4E-02	-6.4E-01	5.2E-01
							10% clump.	-8.7E-02	5.4E-02	-1.6E+00	1.1E-01
							30% disp.	-4.1E-02	5.4E-02	-7.6E-01	4.5E-01
							30% clump.	-5.9E-02	5.4E-02	-1.1E+00	2.8E-01
							50% disp.	-1.3E-01	5.4E-02	-2.4E+00	1.9E-02
							50% clump.	2.5E-02	5.4E-02	4.7E-01	6.4E-01

Model	Species	GCM	RCP	Period	Adj. R^2	Overall *p*	Predictor	Estimate	SE	*t*	*p*
33	Pico	HadGEM2-ES365	4.5	Early	0.44	5.0E-16	Intercept	2.6E+00	2.5E-02	1.0E+02	1.8E-128
							10% disp.	1.8E-01	3.6E-02	5.0E+00	1.8E-06
							10% clump.	1.2E-01	3.6E-02	3.4E+00	9.9E-04
							30% disp.	2.9E-01	3.6E-02	8.0E+00	6.3E-13
							30% clump.	1.6E-01	3.6E-02	4.5E+00	1.5E-05
							50% disp.	3.4E-01	3.6E-02	9.4E+00	2.6E-16
							50% clump.	2.3E-01	3.6E-02	6.5E+00	1.8E-09
34	Pico	HadGEM2-ES365	8.5	Early	0.35	5.1E-12	Intercept	2.7E+00	2.2E-02	1.2E+02	5.8E-139
							10% disp.	6.0E-02	3.2E-02	1.9E+00	5.8E-02
							10% clump.	4.0E-02	3.2E-02	1.3E+00	2.0E-01
							30% disp.	2.1E-01	3.2E-02	6.5E+00	1.4E-09
							30% clump.	1.3E-01	3.2E-02	4.0E+00	9.9E-05
							50% disp.	2.2E-01	3.2E-02	6.9E+00	2.5E-10
							50% clump.	1.4E-01	3.2E-02	4.3E+00	3.4E-05
35	Pico	CanESM2	4.5	Early	0.49	1.5E-18	Intercept	2.8E+00	2.0E-02	1.4E+02	5.7E-146
							10% disp.	8.6E-02	2.8E-02	3.1E+00	2.5E-03
							10% clump.	2.9E-02	2.8E-02	1.0E+00	3.1E-01
							30% disp.	2.0E-01	2.8E-02	7.2E+00	3.2E-11
							30% clump.	1.7E-01	2.8E-02	6.0E+00	1.5E-08
							50% disp.	2.4E-01	2.8E-02	8.7E+00	1.2E-14
							50% clump.	2.1E-01	2.8E-02	7.3E+00	1.9E-11
36	Pico	CanESM2	8.5	Early	0.43	1.4E-15	Intercept	2.8E+00	2.2E-02	1.3E+02	5.9E-140
							10% disp.	7.6E-02	3.1E-02	2.5E+00	1.5E-02
							10% clump.	5.8E-02	3.1E-02	1.9E+00	6.3E-02
							30% disp.	2.2E-01	3.1E-02	7.1E+00	8.2E-11
							30% clump.	1.3E-01	3.1E-02	4.2E+00	5.1E-05
							50% disp.	2.7E-01	3.1E-02	8.7E+00	1.2E-14
							50% clump.	1.6E-01	3.1E-02	5.3E+00	5.8E-07

Table S3. Summary of linear regressions fit to $\log_{10}$(seedling density + 1) five years postfire late (2076 – 2100) in the century with fire exclusion zone scenario as predictor. We used t-values and adjusted R^2 to assess predictor importance, rather than relying on p-values. The effect of each fire exclusion zone scenario is relative to a scenario without fire exclusion zones. Species codes are as follows: Abla = subalpine fir, Pien = Engelmann spruce, Psme = Douglas-fir, PicS = serotinous lodgepole pine, Pico = nonserotinous lodgepole pine.

Model	Species	GCM	RCP	Period	Adj. R^2	Overall *p*	Predictor	Estimate	SE	*t*	*p*
5	Abla	HadGEM2-ES365	4.5	Late	0.59	7.2E-25	Intercept	2.8E+00	4.0E-02	6.9E+01	5.7E-106
							10% disp.	3.9E-01	5.7E-02	6.9E+00	2.4E-10
							10% clump.	2.5E-01	5.7E-02	4.4E+00	1.9E-05
							30% disp.	6.6E-01	5.7E-02	1.2E+01	1.1E-21
							30% clump.	4.1E-01	5.7E-02	7.1E+00	5.8E-11
							50% disp.	7.0E-01	5.7E-02	1.2E+01	1.6E-23
							50% clump.	4.2E-01	5.7E-02	7.3E+00	1.8E-11
6	Abla	HadGEM2-ES365	8.5	Late	0.76	3.0E-40	Intercept	2.5E+00	3.8E-02	6.6E+01	8.1E-104
							10% disp.	5.3E-01	5.4E-02	9.9E+00	9.9E-18
							10% clump.	4.7E-01	5.4E-02	8.8E+00	7.7E-15
							30% disp.	9.3E-01	5.4E-02	1.7E+01	4.3E-36
							30% clump.	5.5E-01	5.4E-02	1.0E+01	1.1E-18
							50% disp.	9.8E-01	5.4E-02	1.8E+01	5.2E-38
							50% clump.	7.0E-01	5.4E-02	1.3E+01	2.3E-25
7	Abla	CanESM2	4.5	Late	0.19	4.6E-06	Intercept	3.1E+00	4.2E-02	7.4E+01	1.2E-109
							10% disp.	2.0E-01	6.0E-02	3.3E+00	1.4E-03
							10% clump.	1.5E-01	6.0E-02	2.6E+00	1.1E-02
							30% disp.	3.2E-01	6.0E-02	5.3E+00	4.4E-07
							30% clump.	1.4E-01	6.0E-02	2.3E+00	2.4E-02
							50% disp.	3.0E-01	6.0E-02	5.0E+00	1.8E-06
							50% clump.	2.3E-01	6.0E-02	3.9E+00	1.7E-04
8	Abla	CanESM2	8.5	Late	0.32	1.1E-10	Intercept	3.0E+00	4.1E-02	7.4E+01	6.2E-110
							10% disp.	1.7E-01	5.8E-02	2.9E+00	4.1E-03
							10% clump.	7.7E-02	5.8E-02	1.3E+00	1.9E-01
							30% disp.	3.8E-01	5.8E-02	6.6E+00	9.5E-10
							30% clump.	1.9E-01	5.8E-02	3.2E+00	1.5E-03
							50% disp.	3.6E-01	5.8E-02	6.2E+00	6.6E-09
							50% clump.	2.8E-01	5.8E-02	4.8E+00	4.9E-06

Model	Species	GCM	RCP	Period	Adj. R^2	Overall *p*	Predictor	Estimate	SE	*t*	*p*
13	Pien	HadGEM2-ES365	4.5	Late	0.06	2.2E-02	Intercept	2.3E+00	9.2E-02	2.5E+01	9.5E-53
							10% disp.	1.4E-01	1.3E-01	1.1E+00	2.9E-01
							10% clump.	1.1E-01	1.3E-01	8.6E-01	3.9E-01
							30% disp.	3.9E-01	1.3E-01	3.0E+00	3.4E-03
							30% clump.	8.4E-02	1.3E-01	6.4E-01	5.2E-01
							50% disp.	3.4E-01	1.3E-01	2.6E+00	9.5E-03
							50% clump.	5.5E-02	1.3E-01	4.2E-01	6.8E-01
14	Pien	HadGEM2-ES365	8.5	Late	0.50	4.0E-19	Intercept	8.6E-01	1.0E-01	8.5E+00	4.3E-14
							10% disp.	6.7E-01	1.4E-01	4.7E+00	7.3E-06
							10% clump.	6.3E-01	1.4E-01	4.4E+00	2.0E-05
							30% disp.	1.3E+00	1.4E-01	9.4E+00	2.3E-16
							30% clump.	6.5E-01	1.4E-01	4.5E+00	1.2E-05
							50% disp.	1.5E+00	1.4E-01	1.0E+01	6.6E-19
							50% clump.	8.3E-01	1.4E-01	5.8E+00	4.9E-08
15	Pien	CanESM2	4.5	Late	-0.04	1.0E+00	Intercept	2.4E+00	1.3E-01	1.9E+01	1.2E-38
							10% disp.	-3.8E-02	1.8E-01	-2.1E-01	8.3E-01
							10% clump.	3.8E-02	1.8E-01	2.1E-01	8.3E-01
							30% disp.	2.3E-02	1.8E-01	1.3E-01	9.0E-01
							30% clump.	8.2E-02	1.8E-01	4.5E-01	6.5E-01
							50% disp.	1.5E-02	1.8E-01	8.2E-02	9.4E-01
							50% clump.	5.5E-02	1.8E-01	3.0E-01	7.6E-01
16	Pien	CanESM2	8.5	Late	0.23	2.1E-07	Intercept	9.5E-01	1.2E-01	7.7E+00	3.3E-12
							10% disp.	6.6E-01	1.7E-01	3.8E+00	2.5E-04
							10% clump.	4.0E-01	1.7E-01	2.3E+00	2.4E-02
							30% disp.	7.0E-01	1.7E-01	4.0E+00	1.0E-04
							30% clump.	5.1E-01	1.7E-01	2.9E+00	3.9E-03
							50% disp.	1.1E+00	1.7E-01	6.5E+00	1.3E-09
							50% clump.	7.5E-01	1.7E-01	4.3E+00	3.4E-05

Model	Species	GCM	RCP	Period	Adj. R^2	Overall *p*	Predictor	Estimate	SE	*t*	*p*
21	Psme	HadGEM2-ES365	4.5	Late	0.06	2.1E-02	Intercept	3.4E+00	3.8E-02	8.9E+01	3.1E-120
							10% disp.	-4.9E-02	5.4E-02	-9.1E-01	3.7E-01
							10% clump.	-6.2E-02	5.4E-02	-1.1E+00	2.6E-01
							30% disp.	-5.5E-02	5.4E-02	-1.0E+00	3.1E-01
							30% clump.	-6.6E-02	5.4E-02	-1.2E+00	2.2E-01
							50% disp.	-1.5E-01	5.4E-02	-2.8E+00	6.2E-03
							50% clump.	-1.8E-01	5.4E-02	-3.2E+00	1.5E-03
22	Psme	HadGEM2-ES365	8.5	Late	0.29	1.9E-09	Intercept	3.6E+00	3.1E-02	1.2E+02	1.8E-136
							10% disp.	-5.3E-02	4.4E-02	-1.2E+00	2.2E-01
							10% clump.	-8.3E-02	4.4E-02	-1.9E+00	6.1E-02
							30% disp.	-1.5E-01	4.4E-02	-3.5E+00	7.2E-04
							30% clump.	-1.7E-01	4.4E-02	-3.8E+00	2.3E-04
							50% disp.	-2.3E-01	4.4E-02	-5.2E+00	6.9E-07
							50% clump.	-2.8E-01	4.4E-02	-6.5E+00	1.5E-09
23	Psme	CanESM2	4.5	Late	0.02	1.6E-01	Intercept	3.0E+00	4.9E-02	6.2E+01	1.1E-99
							10% disp.	-4.3E-02	6.9E-02	-6.2E-01	5.4E-01
							10% clump.	1.4E-02	6.9E-02	2.1E-01	8.3E-01
							30% disp.	7.4E-02	6.9E-02	1.1E+00	2.8E-01
							30% clump.	-5.6E-02	6.9E-02	-8.2E-01	4.1E-01
							50% disp.	-4.3E-02	6.9E-02	-6.2E-01	5.4E-01
							50% clump.	-1.2E-01	6.9E-02	-1.7E+00	9.0E-02
24	Psme	CanESM2	8.5	Late	0.02	1.9E-01	Intercept	3.0E+00	4.1E-02	7.4E+01	5.8E-110
							10% disp.	-4.4E-02	5.8E-02	-7.7E-01	4.4E-01
							10% clump.	-1.2E-02	5.8E-02	-2.1E-01	8.3E-01
							30% disp.	-5.7E-02	5.8E-02	-9.9E-01	3.3E-01
							30% clump.	-8.9E-02	5.8E-02	-1.5E+00	1.3E-01
							50% disp.	-8.1E-02	5.8E-02	-1.4E+00	1.6E-01
							50% clump.	-1.5E-01	5.8E-02	-2.5E+00	1.2E-02

Model	Species	GCM	RCP	Period	Adj. R^2	Overall *p*	Predictor	Estimate	SE	*t*	*p*
29	PicS	HadGEM2-ES365	4.5	Late	0.14	1.5E-04	Intercept	3.7E+00	4.3E-02	8.6E+01	4.0E-118
							10% disp.	-6.4E-02	6.1E-02	-1.0E+00	3.0E-01
							10% clump.	-1.0E-01	6.1E-02	-1.7E+00	8.9E-02
							30% disp.	-2.4E-01	6.1E-02	-4.0E+00	1.0E-04
							30% clump.	-1.8E-01	6.1E-02	-2.9E+00	4.5E-03
							50% disp.	-2.6E-01	6.1E-02	-4.3E+00	3.8E-05
							50% clump.	-1.8E-01	6.1E-02	-3.0E+00	3.3E-03
30	PicS	HadGEM2-ES365	8.5	Late	0.00	4.1E-01	Intercept	3.3E+00	5.0E-02	6.5E+01	7.5E-103
							10% disp.	1.3E-01	7.1E-02	1.8E+00	8.0E-02
							10% clump.	1.3E-01	7.1E-02	1.8E+00	7.2E-02
							30% disp.	1.6E-01	7.1E-02	2.2E+00	3.1E-02
							30% clump.	1.1E-01	7.1E-02	1.6E+00	1.2E-01
							50% disp.	1.3E-01	7.1E-02	1.9E+00	6.0E-02
							50% clump.	1.3E-01	7.1E-02	1.8E+00	7.2E-02
31	PicS	CanESM2	4.5	Late	0.17	1.8E-05	Intercept	3.7E+00	4.6E-02	8.2E+01	1.2E-115
							10% disp.	-9.1E-02	6.5E-02	-1.4E+00	1.6E-01
							10% clump.	-1.5E-01	6.5E-02	-2.3E+00	2.6E-02
							30% disp.	-2.8E-01	6.5E-02	-4.3E+00	2.8E-05
							30% clump.	-1.3E-01	6.5E-02	-1.9E+00	5.4E-02
							50% disp.	-3.3E-01	6.5E-02	-5.0E+00	1.5E-06
							50% clump.	-1.5E-01	6.5E-02	-2.3E+00	2.2E-02
32	PicS	CanESM2	8.5	Late	0.37	1.4E-12	Intercept	3.8E+00	4.6E-02	8.4E+01	4.2E-117
							10% disp.	-1.4E-01	6.4E-02	-2.2E+00	3.3E-02
							10% clump.	-1.5E-01	6.4E-02	-2.3E+00	2.2E-02
							30% disp.	-3.1E-01	6.4E-02	-4.8E+00	4.4E-06
							30% clump.	-1.6E-01	6.4E-02	-2.5E+00	1.2E-02
							50% disp.	-5.5E-01	6.4E-02	-8.5E+00	3.2E-14
							50% clump.	-2.2E-01	6.4E-02	-3.4E+00	8.0E-04

Model	Species	GCM	RCP	Period	Adj. R^2	Overall *p*	Predictor	Estimate	SE	*t*	*p*
37	Pico	HadGEM2-ES365	4.5	Late	0.15	7.3E-05	Intercept	2.6E+00	2.8E-02	9.2E+01	1.9E-122
							10% disp.	9.6E-02	4.0E-02	2.4E+00	1.7E-02
							10% clump.	4.8E-02	4.0E-02	1.2E+00	2.3E-01
							30% disp.	1.4E-01	4.0E-02	3.6E+00	3.9E-04
							30% clump.	1.0E-01	4.0E-02	2.5E+00	1.3E-02
							50% disp.	2.0E-01	4.0E-02	5.0E+00	1.5E-06
							50% clump.	1.1E-01	4.0E-02	2.7E+00	8.4E-03
38	Pico	HadGEM2-ES365	8.5	Late	0.60	9.3E-26	Intercept	2.3E+00	2.8E-02	8.1E+01	2.4E-115
							10% disp.	2.6E-01	4.0E-02	6.6E+00	8.0E-10
							10% clump.	2.3E-01	4.0E-02	5.8E+00	3.7E-08
							30% disp.	4.8E-01	4.0E-02	1.2E+01	7.2E-23
							30% clump.	3.1E-01	4.0E-02	7.9E+00	9.8E-13
							50% disp.	5.1E-01	4.0E-02	1.3E+01	5.3E-25
							50% clump.	3.3E-01	4.0E-02	8.3E+00	9.7E-14
39	Pico	CanESM2	4.5	Late	0.06	3.3E-02	Intercept	2.7E+00	2.7E-02	1.0E+02	8.0E-127
							10% disp.	9.7E-03	3.9E-02	2.5E-01	8.0E-01
							10% clump.	9.0E-03	3.9E-02	2.3E-01	8.2E-01
							30% disp.	5.3E-02	3.9E-02	1.4E+00	1.7E-01
							30% clump.	-4.1E-03	3.9E-02	-1.1E-01	9.1E-01
							50% disp.	5.7E-02	3.9E-02	1.5E+00	1.4E-01
							50% clump.	1.1E-01	3.9E-02	2.9E+00	4.9E-03
40	Pico	CanESM2	8.5	Late	-0.02	7.5E-01	Intercept	2.8E+00	2.5E-02	1.1E+02	2.6E-133
							10% disp.	2.4E-02	3.5E-02	6.7E-01	5.0E-01
							10% clump.	8.6E-03	3.5E-02	2.4E-01	8.1E-01
							30% disp.	4.2E-02	3.5E-02	1.2E+00	2.4E-01
							30% clump.	2.9E-02	3.5E-02	8.2E-01	4.1E-01
							50% disp.	4.3E-02	3.5E-02	1.2E+00	2.3E-01
							50% clump.	-2.3E-03	3.5E-02	-6.5E-02	9.5E-01

Residuals vs Fitted

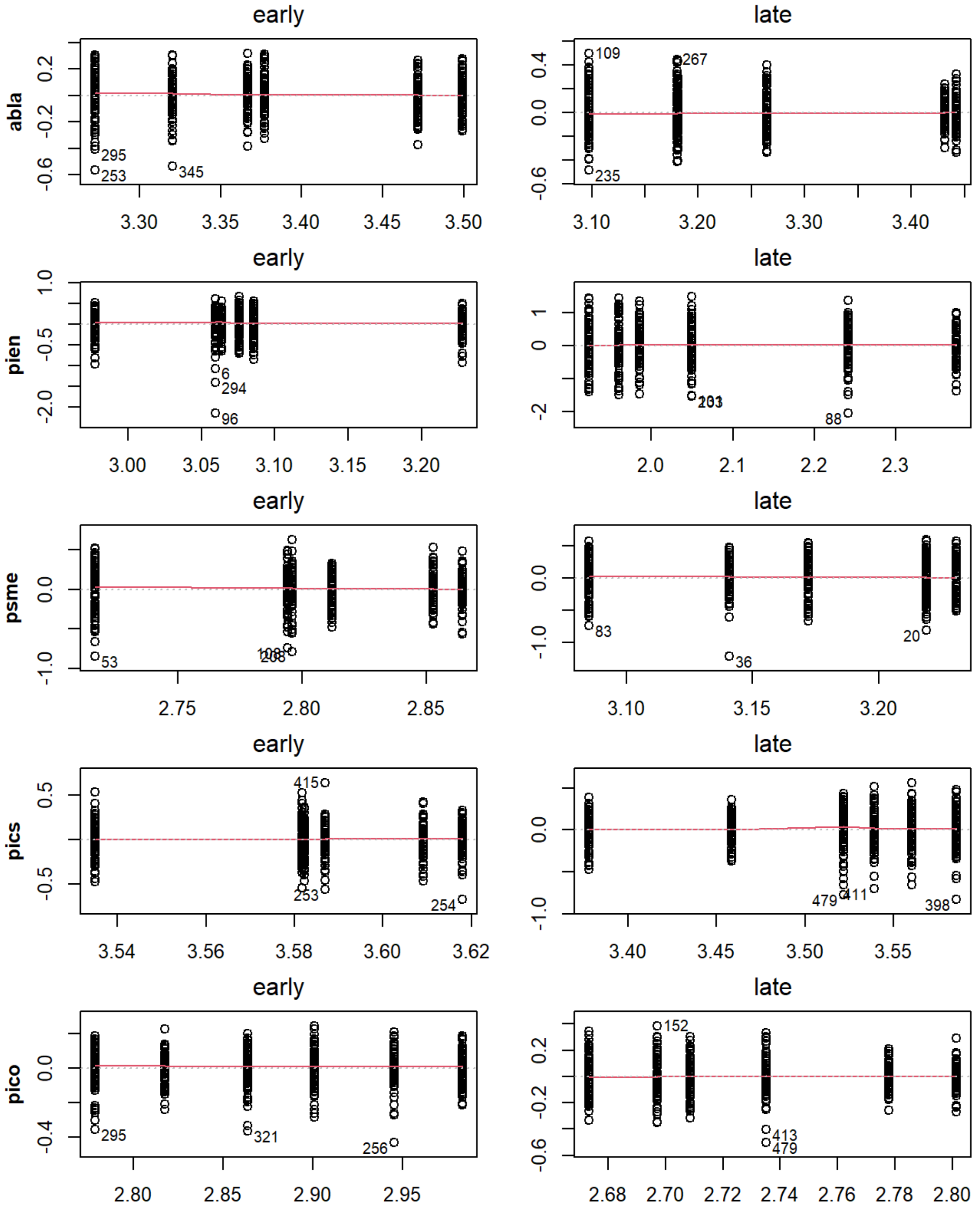

Figure S1: Residuals vs fitted values diagnostic plots for two-way ANOVA fit to $\log_{10}$(seedling density + 1) five years postfire early (2026 – 2050) and late (2076 – 2100) in the century. All models follow the formula $\log_{10}$(mean seedlings ha^{-1} $iteration^{-1}$ + 1) ~ amount × configuration (of fire exclusion zones).

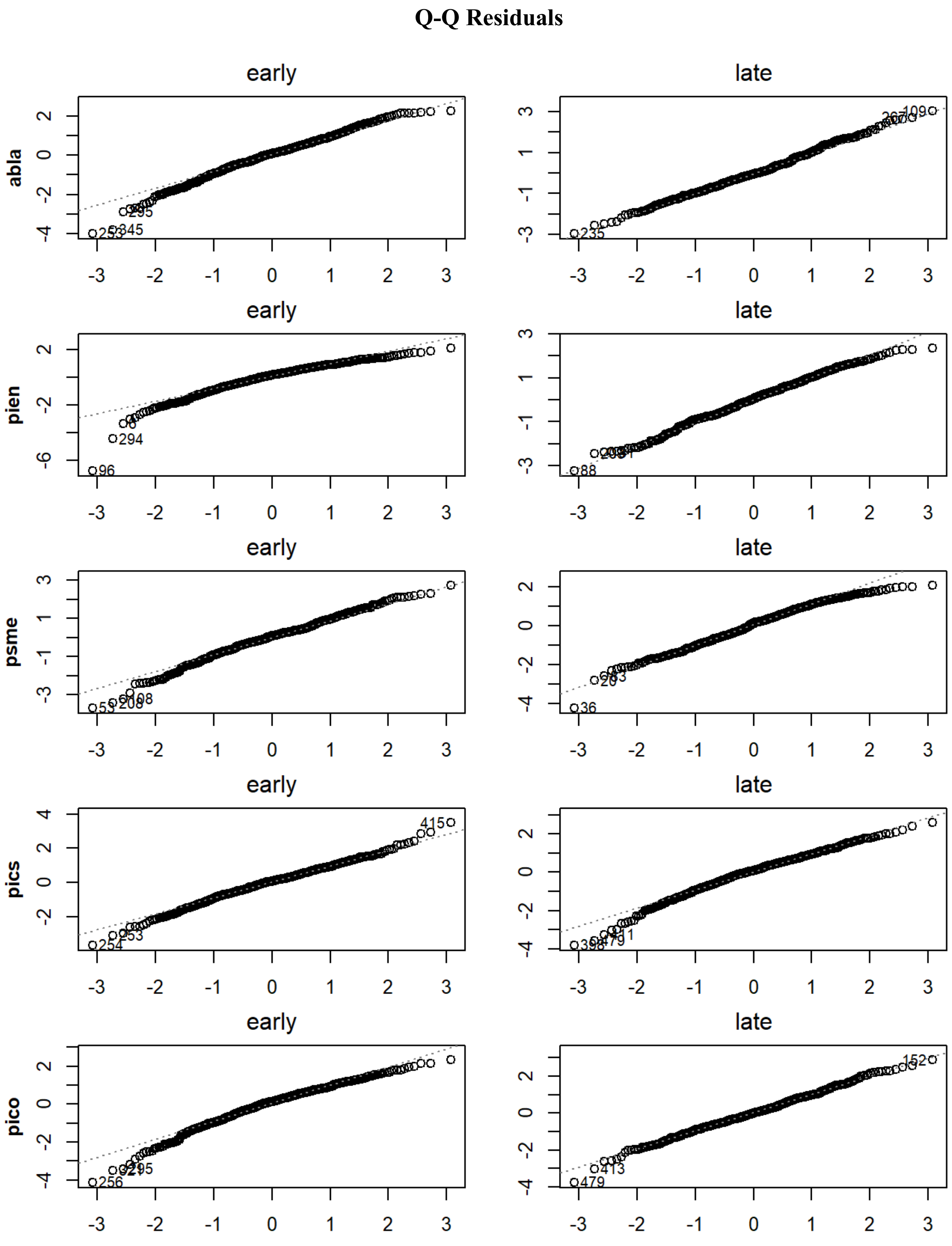

Figure S2: Q-Q Residuals diagnostic plots for two-way ANOVA fit to $\log_{10}$(seedling density + 1) five years postfire early (2026 – 2050) and late (2076 – 2100) in the century. All models follow the formula $\log_{10}$(mean seedlings ha^{-1} iteration^{-1} + 1) ~ amount × configuration (of fire exclusion zones).

Residuals vs Fitted, early (2026-2050) in simulation

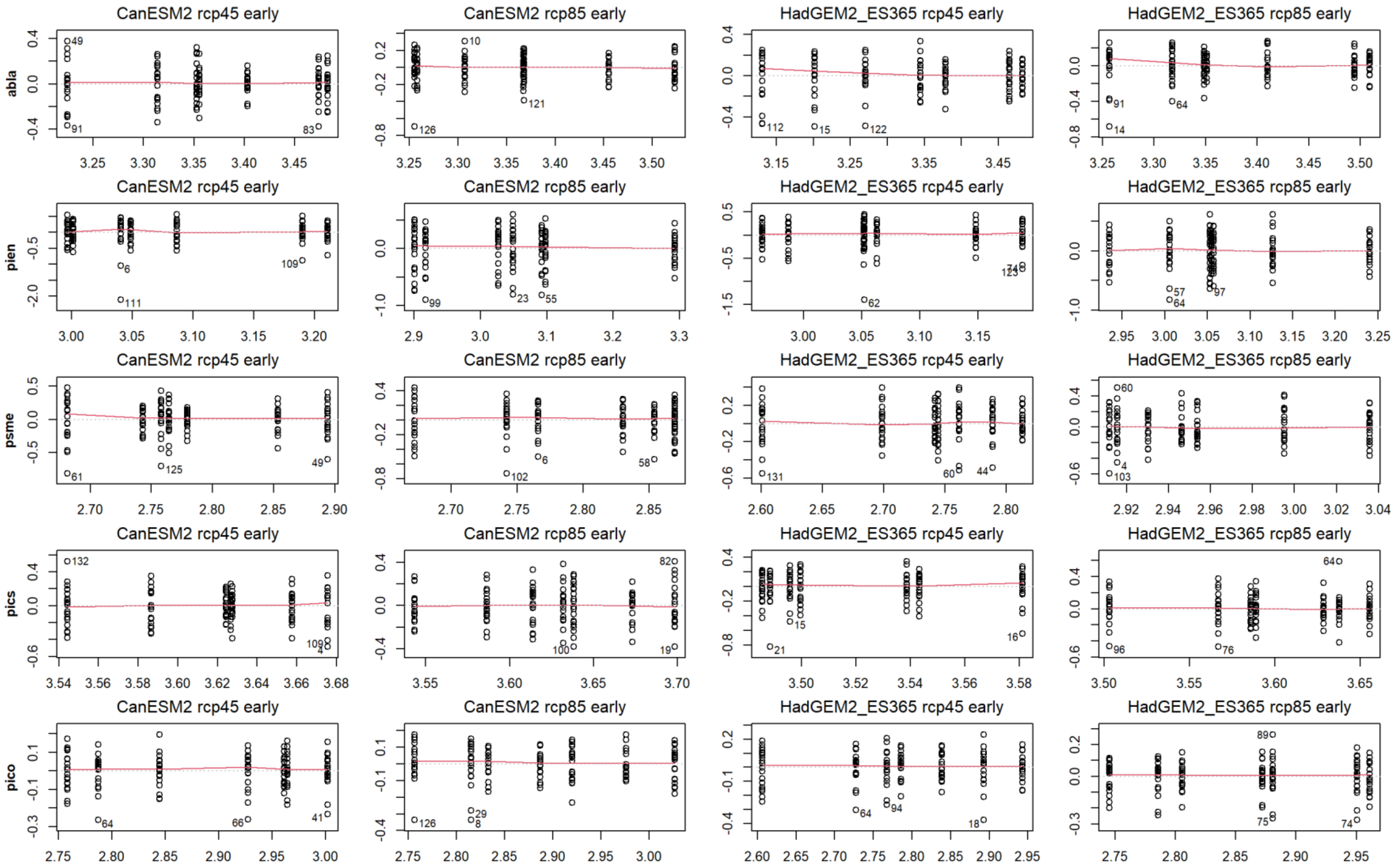

Figure S3. Residuals vs fitted values diagnostic plots of linear regressions fit to $\log_{10}$(seedling density + 1) five years postfire early (2026 – 2050) in the century with fire exclusion zone scenario as predictor. Species codes are as follows: Abla = subalpine fir, Pien = Engelmann spruce, Psme = Douglas-fir, PicS = serotinous lodgepole pine, Pico = nonserotinous lodgepole pine.

Residuals vs Fitted, late (2076-2100) in simulation

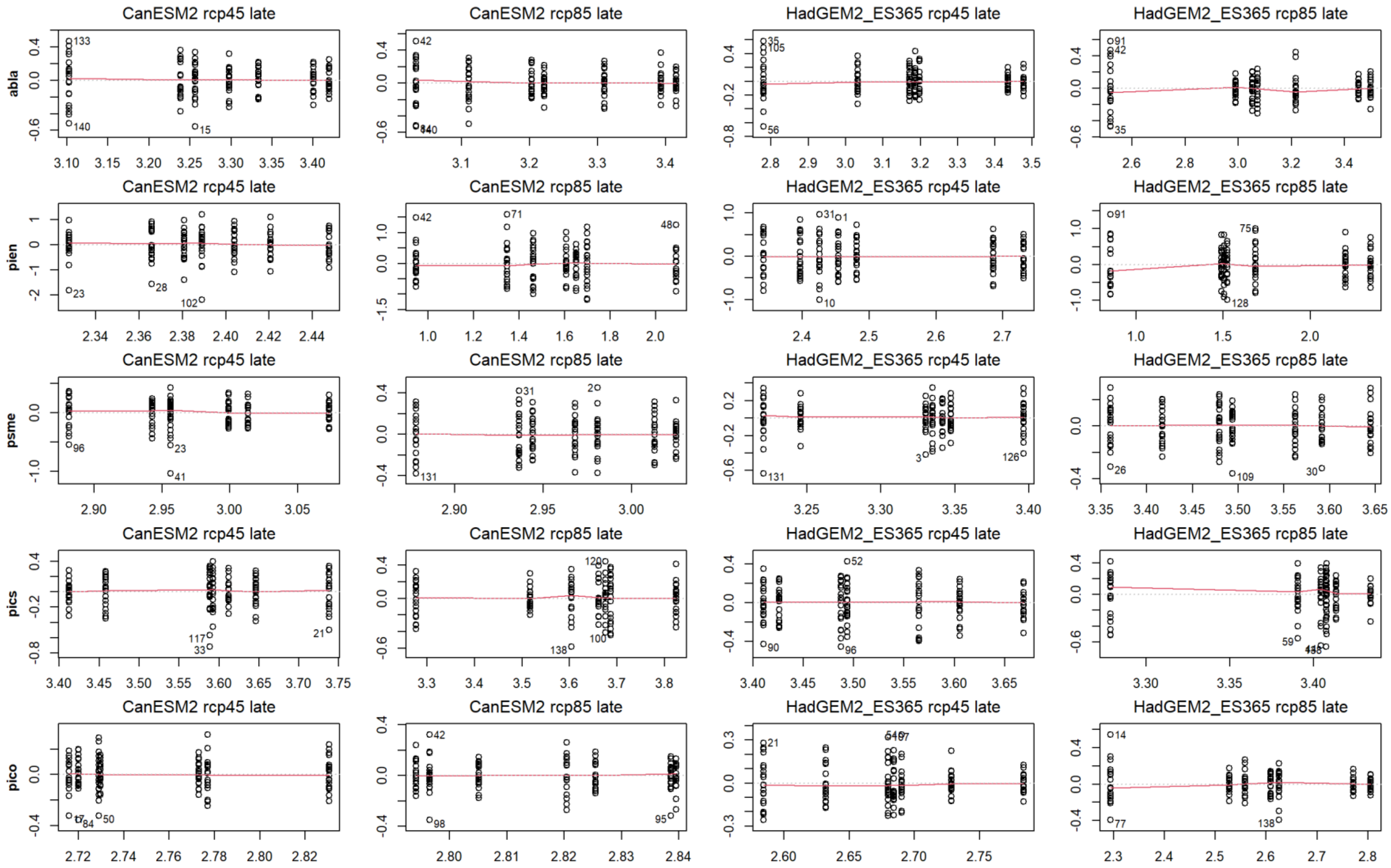

Figure S4. Residuals vs fitted values diagnostic plots of linear regressions fit to log_{10}(seedling density + 1) five years postfire late (2076 – 2100) in the century with fire exclusion zone scenario as predictor. Species codes are as follows: Abla = subalpine fir, Pien = Engelmann spruce, Psme = Douglas-fir, PicS = serotinous lodgepole pine, Pico = nonserotinous lodgepole pine.

Q-Q Residuals, early (2026-2050) in simulation

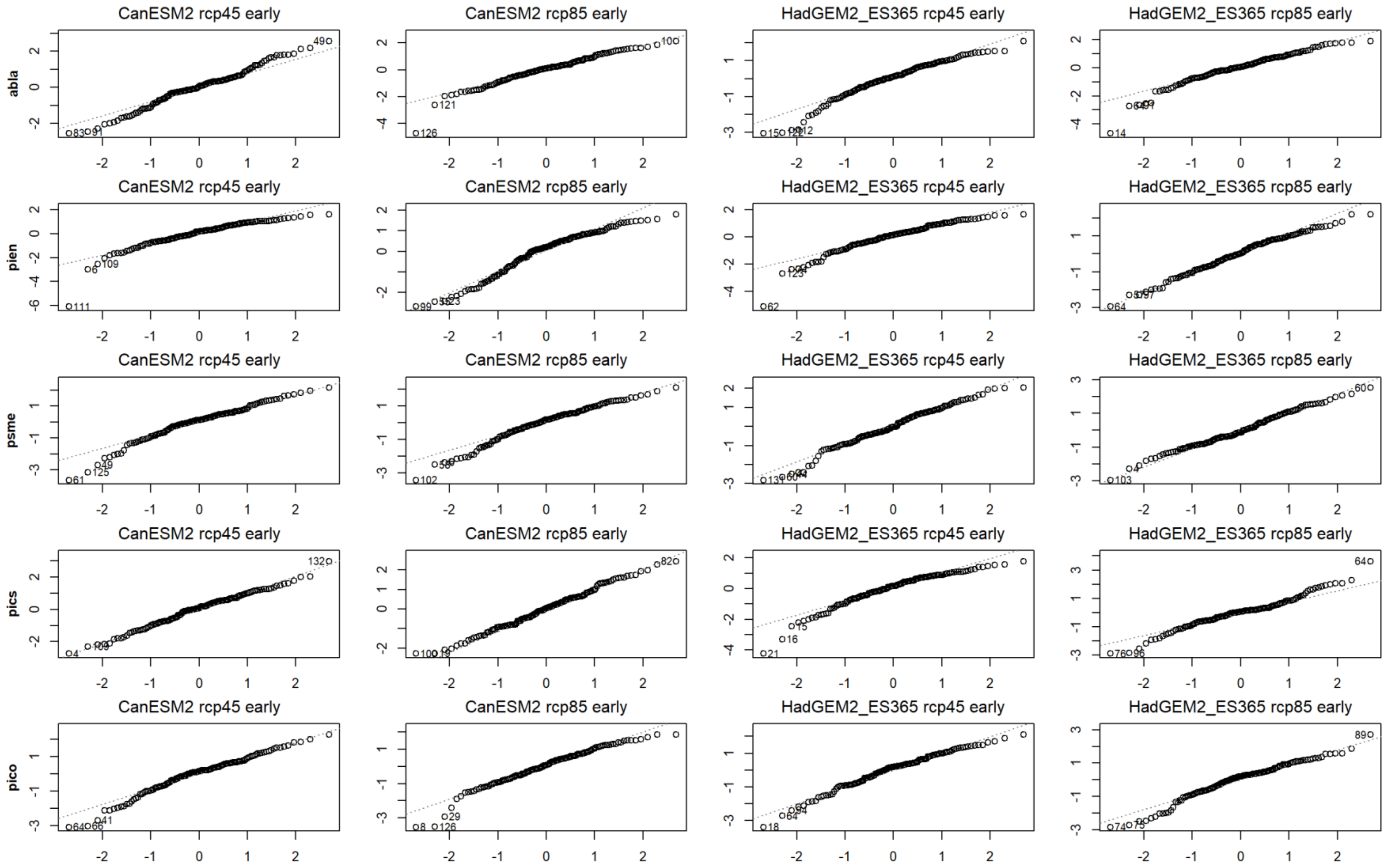

Figure S5. Q-Q Residuals diagnostic plots of linear regressions fit to $\log_{10}$(seedling density + 1) five years postfire early (2026 – 2050) in the century with fire exclusion zone scenario as predictor. Species codes are as follows: Abla = subalpine fir, Pien = Engelmann spruce, Psme = Douglas-fir, PicS = serotinous lodgepole pine, Pico = nonserotinous lodgepole pine.

Q-Q Residuals, late (2076-2100) in simulation

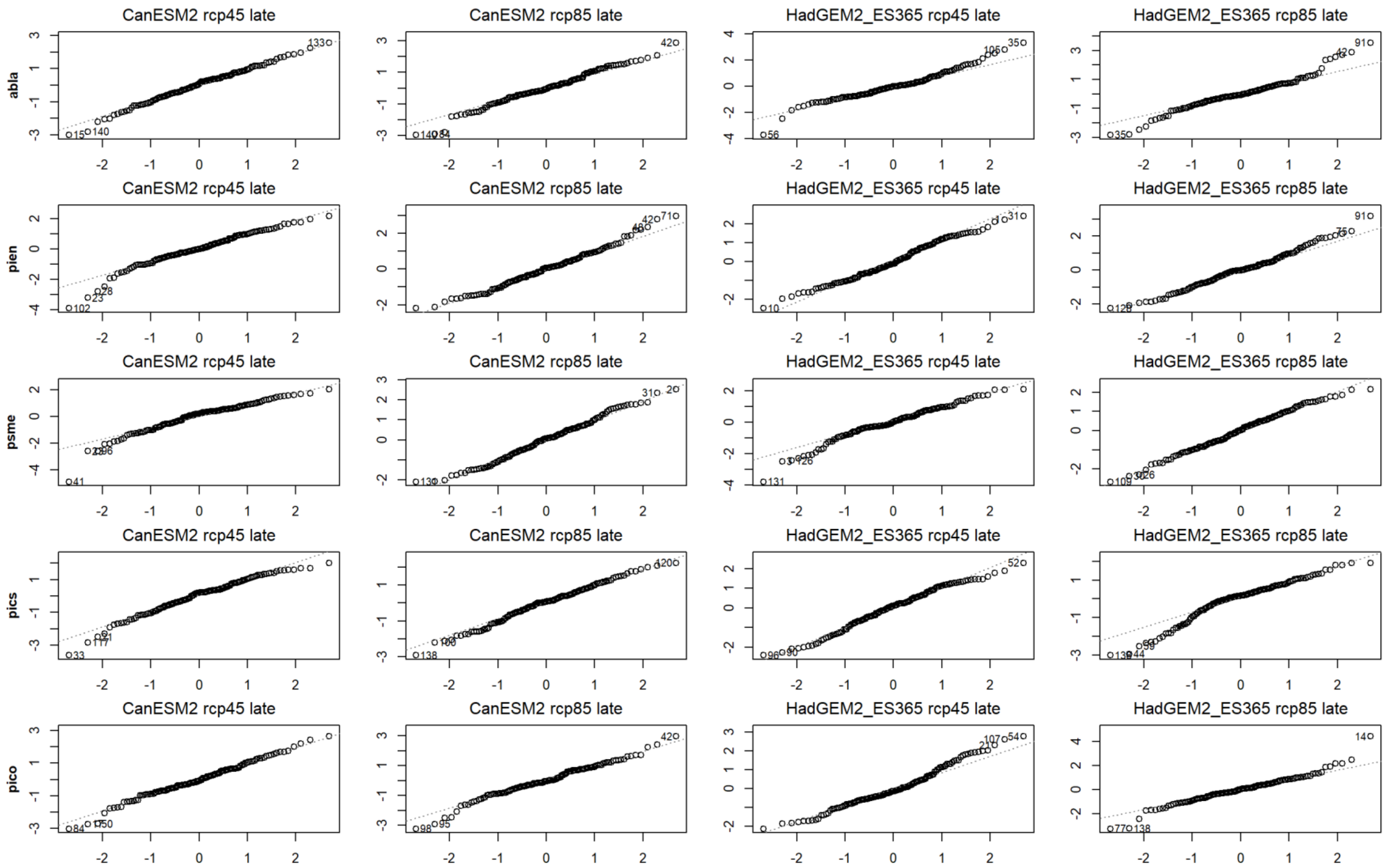

Figure S6. Q-Q Residuals diagnostic plots of linear regressions fit to $\log_{10}$(seedling density + 1) five years postfire late (2076 – 2100) in the century with fire exclusion zone scenario as predictor. Species codes are as follows: Abla = subalpine fir, Pien = Engelmann spruce, Psme = Douglas-fir, PicS = serotinous lodgepole pine, Pico = nonserotinous lodgepole pine.

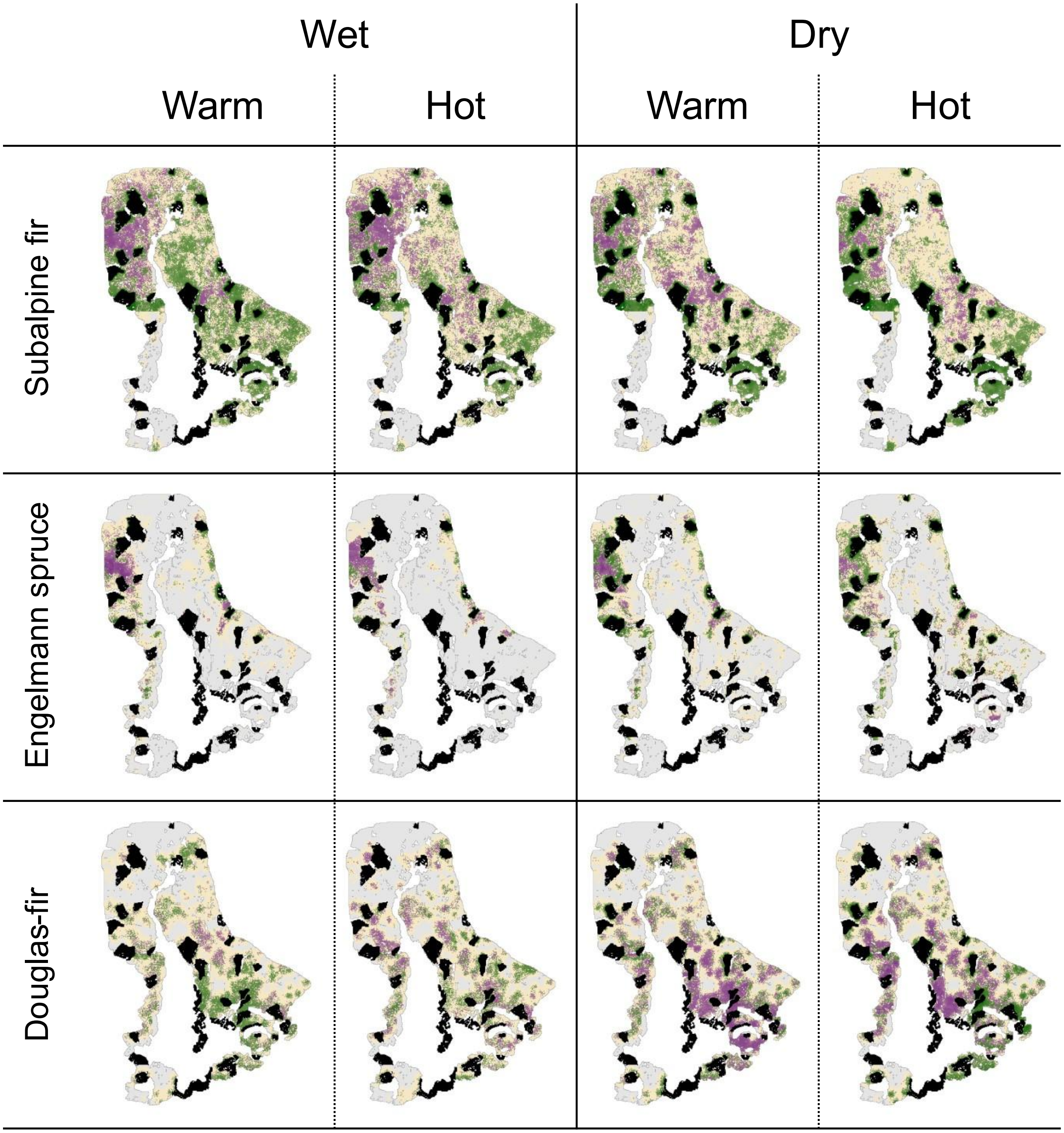

Figure S7: Spatial consequences of simulated operational fire exclusion zones in the four climate scenarios. Areas in purple contained less regeneration in the operational scenario relative to a reference scenario without fire exclusion zones in that cell across 20 iterations, while green areas contained more seedlings in the operational relative to the reference scenario, and beige shows no difference (± 100 stems ha^{-1}) between the two scenarios. Grey is where the species was absent from both operational and reference scenarios, and black areas are operational fire exclusion zones. Regeneration values were $\log_{10}$ transformed before mapping.

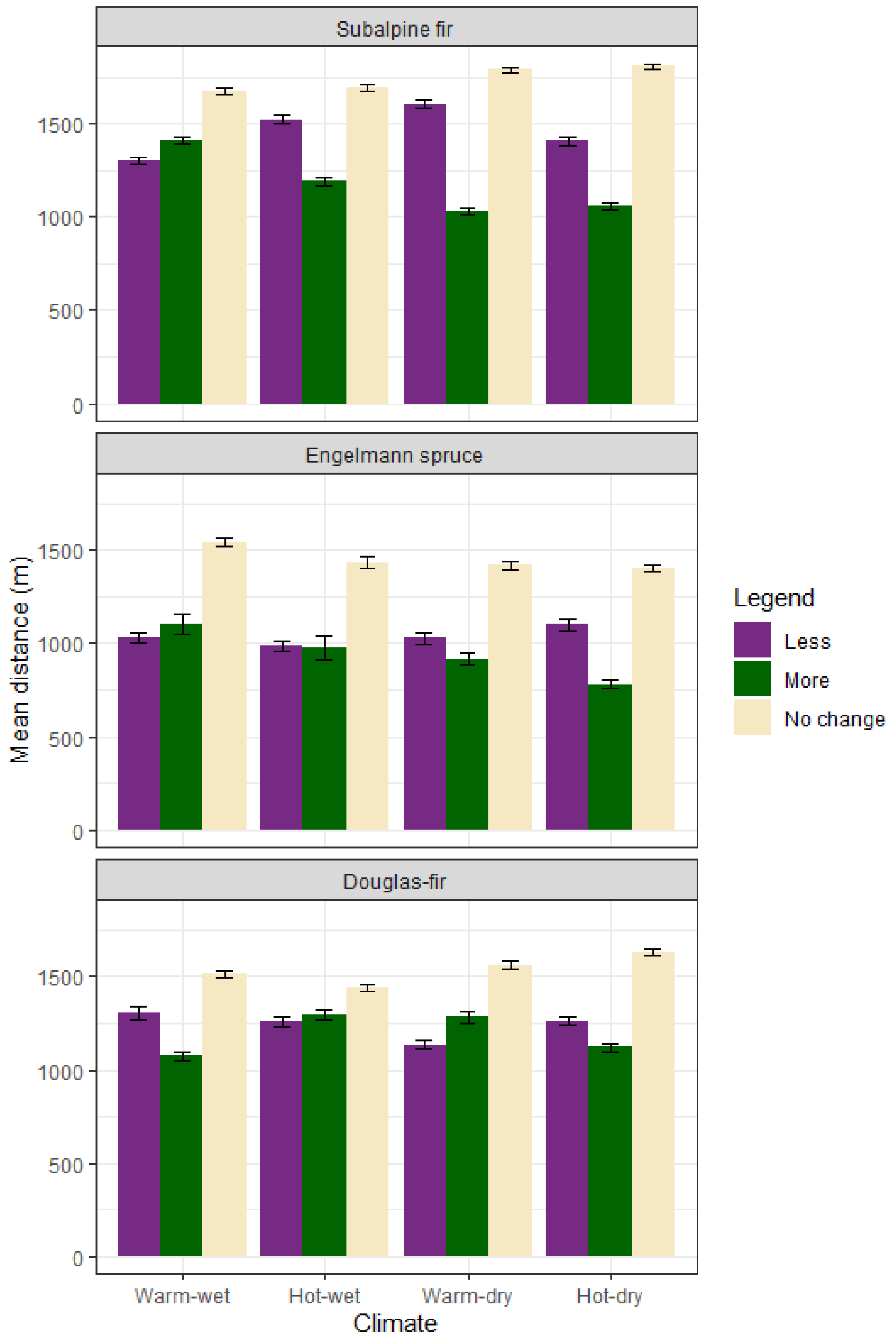

Figure S8: Mean distance to operational fire exclusion zones for cells with more, less, and no difference ($\pm$ 100 stems ha^{-1}) in tree seedling densities in the operational scenario relative to the reference scenario without fire exclusion zones.
